# Understanding chronic inflammation: couplings between cytokines, ROS, NO, Ca_i_^2+^, HIF-1α, Nrf2 and autophagy

**DOI:** 10.3389/fimmu.2025.1558263

**Published:** 2025-04-08

**Authors:** Krzysztof Piotr Michalak, Amelia Zofia Michalak

**Affiliations:** ^1^ Laboratory of Vision Science and Optometry, Physics and Astronomy Faculty, Adam Mickiewicz University in Poznań, Poznań, Poland; ^2^ Faculty of Medicine, Poznań University of Medical Sciences, Poznań, Poland

**Keywords:** cytokines, inflammation, NF-κB, iNOS, nitric oxide, autophagy, HIF-1α, calcium flux

## Abstract

Chronic inflammation is an important component of many diseases, including autoimmune diseases, intracellular infections, dysbiosis and degenerative diseases. An important element of this state is the mainly positive feedback between inflammatory cytokines, reactive oxygen species (ROS), nitric oxide (NO), increased intracellular calcium, hypoxia-inducible factor 1-alpha (HIF-1α) stabilisation and mitochondrial oxidative stress, which, under normal conditions, enhance the response against pathogens. Autophagy and the nuclear factor erythroid 2-related factor 2 (Nrf2)-mediated antioxidant response are mainly negatively coupled with the above-mentioned elements to maintain the defence response at a level appropriate to the severity of the infection. The current review is the first attempt to build a multidimensional model of cellular self-regulation of chronic inflammation. It describes the feedbacks involved in the inflammatory response and explains the possible pathways by which inflammation becomes chronic. The multiplicity of positive feedbacks suggests that symptomatic treatment of chronic inflammation should focus on inhibiting multiple positive feedbacks to effectively suppress all dysregulated elements including inflammation, oxidative stress, calcium stress, mito-stress and other metabolic disturbances.

## 1 Introduction

Chronic inflammation is a major medical problem that poses enormous diagnostic and therapeutic challenges worldwide. Despite major advances in recent years, available therapies are often unsatisfactory. Understanding the molecular changes that occur under this condition is essential for the development of effective, comprehensive therapeutic approaches. The immune system is a highly complex self-regulatory system characterised by numerous self-regulatory couplings that adapt the strength and type of response to the nature of the pathogen. The current work extends this analysis to include other elements of cellular self-regulation that are in predominantly positive feedback with inflammatory mediators and with each other, thereby helping to drive the inflammatory response. These elements are oxidative stress, represented by the activity of NADPH oxidases (NOXs), inducible nitric oxide synthase (iNOS) and mitochondrial reactive oxygen species (mito-ROS) (electron leakage from the cytochrome chain), calcium stress (an increase in intracellular calcium concentration and endoplasmic reticulum stress) and hypoxia-inducible factor 1-alpha (HIF-1α), induced under both anaerobic and aerobic conditions. As these elements are mainly in positive feedback with each other, they are referred to in the current work as the Positive Coupling System (PCS).

The intensity of the inflammation must be high enough to fight the infection but not to the point of self-destruction. The regulatory factors are mainly the transcription factors nuclear factor erythroid 2-related factor 2 (Nrf2)/FOXO, which promote antioxidation and autophagy. The following sections will mainly discuss the negative feedbacks between them and the PCS elements.

HIF-1α is a double-faced factor because it is involved in driving up the inflammatory spiral and also has a protective effect on the mitochondria by protecting them from free radical damage. Nitric oxide (NO) produced by iNOS can also activate and inhibit the inflammatory spiral, depending on the metabolic context.

Increasing knowledge about the mutual feedbacks between the mentioned elements of self-regulation allows us to build generalised models of their common interactions. The construction of generalised models is becoming a new challenge at the current level of knowledge and, in the opinion of the authors, will represent a new direction in the development of molecular biology.

The details of the common relationships between the analysed elements are presented in the following sections of the paper. The first part of the paper discusses the basic signalling pathways involved in the transmission of information between analysed elements. The second part discusses the mainly positive feedbacks between inflammation, reactive oxygen species (ROS), NO, Ca_i_^2+^ and HIF-1α. The third part discusses the controlling role of autophagy and Nrf2/FOXO and their inhibitory effects on the mentioned positively coupled elements.

### 1.1 Chronic inflammation

Chronic inflammation is usually generated in one of four cases: 1) the chronic presence of an intracellular pathogen in the cell (1–6), 2) an autoimmune response induced by immune cells against their own tissues in the absence of the pathogen, 3) pathological gut microbiota inducing the chronic inflammation (7) and 4) another metabolic condition or disease in which the initiating factor is another disturbance coupled with inflammation, e.g. oxidative stress, impaired autophagy and calcium stress, which may occur in the course of certain diseases such as atherosclerosis, neurodegenerative diseases and intoxication (8).

In chronic inflammation, an equilibrium develops between a destructive factor (e.g. an intracellular pathogen) and repair factors that are unable to restore the cell or tissue to a healthy state. To understand the problem of chronic inflammation, it is necessary to know the detailed molecular regulatory mechanisms that control this process, both at the local level, i.e. short self-regulatory loops (e.g. stimulation of calcium efflux from the cell when its intracellular concentration increases), and at the global level, i.e. interactions between functionally distant elements such as HIF-1α, Ca_i_^2+^, O_2_^−^/H_2_O_2_, NO, Nrf2 and autophagy. Such interactions are just the subject of the current work.

When analysing the many feedbacks between the many regulatory elements of the cell, it is often difficult to identify the initiating factor, the so-called first domino, that sets off the cascade of molecular perturbations. Identifying such a factor is very important for restoring balance in the cell, but it may not be enough if the system has drifted far from a healthy state. The underlying cause is different in the four types of chronic inflammation mentioned above. In chronic intracellular infections, it is most often the pathogen itself (1–6). In degenerative diseases such as Alzheimer’s or Parkinson’s, there are abnormal proteins (β-amyloid and tau) that disrupt many metabolic pathways (9). In autoimmune diseases without specific foreign initiating proteins, the question of the initiating factor is more complex. It may be abnormal autoantibodies that react with surface proteins and induce a variety of abnormal intracellular responses (10). In the case of intestinal microbiota dysbiosis, the cause of chronic inflammation is the intestinal pathogens that induce low-level inflammation in the intestinal mucosa, which then spreads to the whole organism (11).

An analysis of the literature shows that in intracellular pathogens and degenerative diseases, the common denominator of molecular pathology is blocked autophagy, which prevents the removal of abnormal proteins and sets in motion the inflammatory-oxidative spiral. Autophagy will therefore be an important point of analysis in the current work. Another important issue of great complexity is the process of resolution of inflammation, which requires specific and individual review, especially in the context of the feedbacks presented, because the entry into a chronic state may also depend on the inability of the regulatory system to activate the resolution process despite the fact that the initiating pathogen has been removed.

## 2 Kinase pathways and inflammation

**Figure 1:**
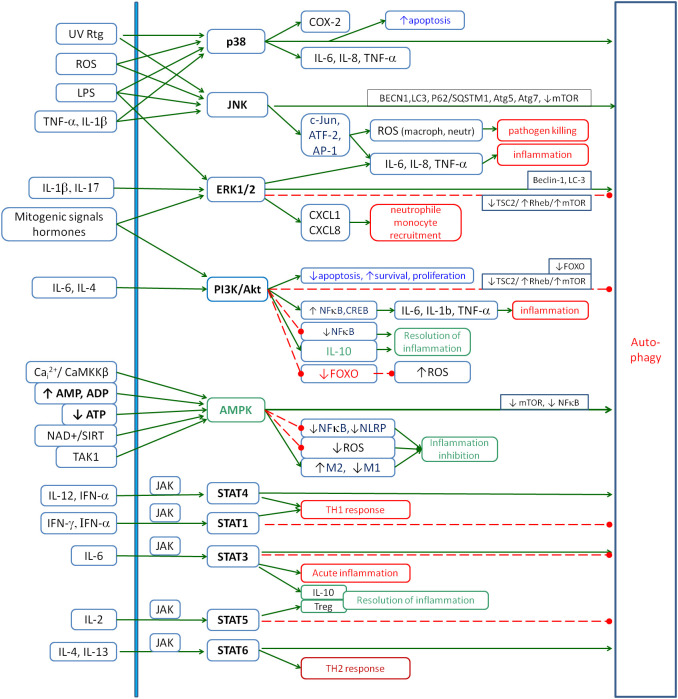
The role of the main signalling pathways involved in the inflammatory response: p38, JNK, ERK1/2, PI3K/Akt, AMPK and JAK/STAT. The figure shows the main factors that activate these pathways and their main inflammatory effects. Special attention is given to their influence on autophagy as one of the key processes involved in chronic inflammation. Solid arrows, activation; red dashed lines with •, inhibition. JNK, c-Jun N-terminal kinase; ERK1/2, extracellular signal-regulated kinases 1 and 2; AMPK, AMP-activated protein kinase; JAK, Janus kinase; STAT, signal transducer and activator of transcription.

Current work focuses on feedback analysis between cytokines, ROS, NO, Ca_i_^2+^, HIF-1α, Nrf2 and autophagy. Signalling pathways such as mitogen-activated protein kinases (MAPKs) [p38, c-Jun N-terminal kinase (JNK) and extracellular signal-regulated kinases 1 and 2 (ERK1/2)], PI3K/Akt, Janus kinase/signal transducer and activator of transcription (JAK/STAT), AMP-activated protein kinase (AMPK) and cAMP/protein kinase A (PKA) are strongly involved in the communication between these elements. These signalling pathways play numerous roles in cellular self-regulation and are already relatively well-understood mechanisms of intracellular communication. These pathways are involved in the transduction of many signals, including those between the elements analysed. Let us summarise the information on these pathways with a focus on inflammation. The second key point is the role of the subsequent pathways in the regulation of autophagy because it seems to be a common feature of very different types of chronic inflammation. If it is impaired, the accumulation of cellular debris and pathogens maintains inflammation, and there is no way to bypass this mechanism of inflammatory induction. A summary of these relationships is shown in **Figure 1**.

### 2.1 P38 MAPK

The p38 MAPK signalling pathway is one of the key regulators of cellular responses to a variety of stimuli, including environmental stresses and inflammatory signals. It is mainly activated by stress factors [oxidative stress, hypoxia, ultraviolet (UV) or ionising radiation, and osmotic disturbances] (12), inflammatory factors [e.g. TNF-α (13, 14), IL-1β (15, 16) and transforming growth factor beta (TGF-β) (17, 18)], pathogens [bacterial lipopolysaccharides (LPS) from bacteria (19) and activating Toll-like receptors (TLRs)] and surface receptor interactions (integrin and Vascular Endothelial Growth Factor receptors in the endothelium) (20). Inhibitors of the p38 MAPK pathway include natural regulatory mechanisms such as MAPK phosphatases (MKPs) and proteins that block kinase interactions with their substrates (12). The activation of the p38 MAPK pathway leads to several biological effects, the most important of which are the activation of genes encoding cytokines such as IL-6, IL-8 or TNF-α (21–23), and the induction of cyclooxygenase-2 (COX-2) expression (24, 25), which increases prostaglandin production. The p38 MAPK pathway also plays a key role in apoptosis by activating proapoptotic proteins in response to cellular stress (26, 27). In addition, p38 MAPK regulates the cell cycle by arresting it in the G1 or G2/M phase in response to DNA damage (28). In the tumour microenvironment, this pathway promotes angiogenesis through VEGF stimulation and promotes tumour cell invasion and survival (29). It is thus a central regulator of the cellular response to environmental and inflammatory stimuli. Its precise regulation is crucial for maintaining cellular homeostasis, and dysregulation of this pathway can lead to inflammatory, cancer and neurodegenerative diseases. p38 MAPK is also involved in activating the autophagy process (30).

### 2.2 ERK1/2

The ERK1/2 signalling pathway plays a key role in the regulation of a variety of biological processes including cell proliferation, differentiation, survival and migration. The activation of the ERK1/2 pathway is associated with responses to mitogenic signals such as growth factors, as well as hormonal stimuli and changes in the extracellular environment. It also plays an important role in the regulation of inflammatory processes, controlling the expression of genes associated with the immune response and the production of cytokines and chemokines (31). Its activation occurs in response to inflammatory stimuli such as pro-inflammatory cytokines (e.g. IL-17A and IL-1β) (16, 32), bacterial LPS (33), growth factors (e.g. VEGF and EGF) (34) and interactions with TLRs and chemokine receptors (31). Receptor tyrosine kinases (RTKs) or G protein-coupled receptors (GPCRs) play a key role in these processes by transducing the signal through the activation of the kinase cascade, leading to the phosphorylation and activation of ERK1/2 by MEK1/2 kinase (31).

The activation of the ERK1/2 pathway promotes the synthesis of pro-inflammatory cytokines, such as IL-6, IL-8, TNF-α and IL-1β, and chemokines, such as CXCL1 and CXCL8, which are responsible for the recruitment of neutrophils and monocytes to the site of inflammation (31, 35–37). The ERK1/2 pathway also promotes phagocytosis (38) and plays a role in stimulating angiogenesis by regulating VEGF expression, which improves blood supply to the inflamed area (39, 40). Excessive or uncontrolled activation of the ERK1/2 pathway leads to chronic inflammatory conditions such as rheumatoid arthritis (41), psoriasis (41) and Crohn’s disease (42), where it can cause abnormal activation of T and B lymphocytes, leading to autoimmunity.

The ERK1/2 signalling pathway is a promising therapeutic target. MEK1/2 inhibitors, such as trametinib, have been used to treat diseases associated with the over-activation of this pathway, including certain cancers (43). In the context of inflammation, ERK1/2 inhibitors reduce the production of pro-inflammatory cytokines and chemokines (31, 44, 45).

This signalling pathway affects autophagy in different ways, depending on the context and the details of the interaction. Under conditions of cellular stress and nutrient deprivation, ERK1/2 promotes autophagy by activating the Beclin-1 protein, but this effect is thought to be at least partly downstream of PI3K/Akt inhibition (46, 47). In contrast, under favourable conditions of cellular growth and proliferation, ERK1/2 can inhibit autophagy by activating mTOR through the TSC2/Rheb/mTORC1 pathway, which is particularly prevalent in cancer (48) and is thought to be also important in neurodegeneration (49).

### 2.3 JNK

The JNK signalling pathway plays an important role in the regulation of inflammatory responses, acting as a mediator of stress signalling, cytokine production and immune cell activity. This pathway is activated by a variety of stimuli, including pro-inflammatory cytokines such as TNF-α and IL-1β (50), ROS (51), LPS and environmental stressors such as UV radiation and osmotic stress (52). Activation occurs via upstream kinases such as MAP kinase kinase 4/7 (MKK4/7), which phosphorylates and activates JNK. JNK, in turn, translocates to the nucleus to regulate the activity of transcription factors such as c-Jun, ATF-2 and AP-1, thereby driving the expression of inflammatory genes.

In the context of inflammation, the JNK pathway is one of the key regulators of cytokine production. By activating the transcription factor AP-1, JNK enhances the expression of pro-inflammatory mediators such as IL-6, IL-8, and TNF-α and chemokines that attract immune cells to the site of inflammation (53–55). JNK also influences processes such as apoptosis and proliferation (56). In macrophages and neutrophils, JNK promotes the production of ROS, which contributes to the destruction of pathogens but can also lead to tissue damage if uncontrolled (57). JNK also plays a role in the resolution of inflammation by promoting apoptosis in damaged or dysfunctional cells, thereby limiting excessive inflammation and maintaining tissue homeostasis (58), and by promoting autophagy, which reduces debris-mediated inflammation (58, 59).

Chronic activation of JNK has been implicated in autoimmune diseases such as rheumatoid arthritis (60), where it contributes to the sustained production of pro-inflammatory cytokines and tissue damage. In metabolic diseases such as obesity and type 2 diabetes, JNK activation in adipose tissue and the liver is associated with insulin resistance and systemic inflammation (61). In addition, in cancer, JNK can promote tumour progression by supporting an inflammatory microenvironment that promotes angiogenesis, invasion and metastasis (62, 63). Targeting the JNK pathway has emerged as a potential therapeutic strategy for inflammatory and autoimmune diseases (52).

### 2.4 PI3K/Akt

The PI3K/Akt signalling pathway plays a role in balancing pro- and anti-inflammatory processes. It is activated in response to a variety of extracellular stimuli, including cytokines, growth factors and pathogen-associated molecular patterns (PAMPs). In the context of inflammation, the PI3K/Akt pathway promotes the survival and activation of macrophages, neutrophils and lymphocytes (64, 65). By activating transcription factors such as NF-κB and cAMP response element-binding protein (CREB), Akt facilitates the production of pro-inflammatory cytokines such as IL-6, IL-1β and TNF-α, which amplifies the inflammatory response (66–70). In addition, Akt promotes the production of chemokines that attract immune cells to the site of inflammation (71).

At the same time, the PI3K/Akt pathway is important for preventing excessive or chronic inflammation by supporting the production of anti-inflammatory IL-10 and other regulatory cytokines (72–74). Akt also negatively regulates inflammation through its inhibitory interactions with downstream molecules such as GSK-3β (glycogen synthase kinase-3β, activator of NF-κB) (75, 76). Akt plays a role in the resolution phase of inflammation by promoting the survival and phagocytic activity of macrophages during the clearance of apoptotic cells and debris, a process known as efferocytosis.The hyperactivation of this pathway can contribute to chronic inflammation under conditions such as rheumatoid arthritis, inflammatory bowel disease and asthma, where it drives sustained immune cell activation and cytokine production (77, 78). Conversely, insufficient PI3K/Akt signalling can impair anti-inflammatory mechanisms and promote uncontrolled inflammation, as seen in certain autoimmune diseases (79). In addition to inflammatory diseases, excessive PI3K/Akt signalling has been implicated in cancer, where it supports an inflammatory tumour microenvironment that promotes angiogenesis, immune evasion and metastasis (80). In metabolic disorders such as obesity and type 2 diabetes, chronic inhibition of the PI3K/Akt pathway in adipose tissue and other organs is associated with insulin resistance and low-grade systemic inflammation (81, 82). In the context of autophagy, PI3K/Akt inhibits it mainly through the TSC2/Rheb/mTORC1 pathway (48, 83). However, inhibition of FOXO by Akt in some cell types leads to inhibition of autophagy, inhibition of antioxidant enzyme production and inhibition of apoptosis, which promotes chronic inflammation (84, 85). In conclusion, the influence of this pathway on inflammation is complex, non-linear, and concentration- and metabolic context-dependent and requires further in-depth analysis.

### 2.5 JAK/STAT

The JAK/STAT signalling pathway plays an important role in inflammation, mediating the effects of cytokines and growth factors that regulate immune responses, cell survival, proliferation and differentiation (86, 87). This pathway is activated by the binding of cytokines, such as interferons (IFNs), interleukins (ILs) and tumour necrosis factor (TNF), to their respective receptors on the cell surface. Upon ligand binding, receptor-associated JAKs are activated by autophosphorylation, creating docking sites for STAT proteins. STAT proteins are then phosphorylated, dimerised and translocated to the nucleus, where they regulate the expression of genes involved in inflammatory and immune responses. The JAK/STAT signalling pathway is central to the regulation of both acute and chronic inflammation. Different STAT proteins are activated by different cytokines. STAT1 is activated by the interferons IFN-α and IFN-γ. It drives the Th1 response important for intracellular pathogen defence and regulates the expression of genes involved in antiviral immunity and macrophage activation (88). STAT3 is activated by IL-6 and promotes the transcription of genes (including through interactions with NF-κB) that sustain the inflammatory process, including acute-phase proteins and chemokines such as CXCL1 and CCL2, which recruit immune cells to sites of inflammation (89, 90). This pathway also promotes cancer progression by activating pro-cancer inflammation. However, STAT3 also drives the production of IL-10 to enter the resolution phase of inflammation. STAT4 is activated by IL-12 and IFN-α and drives the differentiation of Th1 cells, which produce IFN-γ and enhance the pro-inflammatory response (91). STAT5 supports the expansion of regulatory T cells (Tregs) in response to IL-2, contributing to the resolution of inflammation and maintenance of immune tolerance (92). STAT6 is activated by IL-4 and IL-13 and promotes the differentiation of Th2 cells, which are involved in anti-parasite immunity and allergic inflammation (93). The effects of individual JAK/STAT pathways on autophagy vary, depending on the type of pathway and also the metabolic context. A predominantly activating effect is observed for the STAT4 and STAT6 pathways, whereas a predominantly inhibitory effect is observed for the STAT1 and STAT5 pathways. The STAT3 pathway is the most dependent on the metabolic context.

Dysregulation of the JAK/STAT pathway is implicated in many inflammatory and autoimmune diseases. Sustained activation of STAT3 has been implicated in diseases such as rheumatoid arthritis, inflammatory bowel disease and psoriasis, where it drives the sustained production of inflammatory cytokines and tissue damage (94, 95). Inflammatory signals mediated by STAT3 and STAT5 can promote tumourigenesis by supporting angiogenesis, immune evasion and tumour cell survival (96).

### 2.6 AMPK

AMPK is one of the most important pro-regenerative and anti-inflammatory pathways in metabolic self-regulation. It is activated by liver kinase B1 (LKB1) in response to metabolic stress, hypoxia or exercise when cellular ATP levels decrease. AMP or ADP levels increase its activity. A high NAD^+^/NADH ratio also induces this kinase via SIRT1, which deacetylates LKB1 and facilitates its phosphorylation (97). Conversely, AMPK increases the NAD^+^/NADH ratio by several mechanisms, thus closing the positive loop that drives cell regeneration (98, 99). It is noteworthy that calcium/calmodulin-dependent protein kinase kinase β (CaMKKβ) can activate AMPK through LKB1 in response to increased cellular Ca^2+^ levels, and this effect is independent of the AMP-to-ATP ratio (100, 101). The next activator of AMPK is the TAK1 protein (TGF-β-activated kinase 1), which activates AMPK in response to inflammation and stress signals (102). The main role of AMPK is to activate the catabolic and inhibit the anabolic processes. AMPK exerts potent anti-inflammatory effects primarily by inhibiting pro-inflammatory pathways. It suppresses NF-κB signalling, a key regulator of inflammatory cytokines, by phosphorylating and inhibiting IκB kinase (IKK) (103). In addition, AMPK inhibits the activation of the NLRP3 inflammasome, a multi-protein complex responsible for the production of IL-1β (104). It also improves mitochondrial function and reduces levels of ROS (105). Together, these actions collectively reduce inflammation at the molecular level.

Another role of AMPK in inflammation is its ability to promote the anti-inflammatory M2 macrophage phenotype over the pro-inflammatory M1 phenotype (97, 101). M2 macrophages produce cytokines such as IL-10 and TGF-β, which facilitate tissue repair and resolution of inflammation. AMPK by promoting autophagy via mTOR inhibition helps maintain cellular homeostasis and resolve inflammatory signals (106, 107).

Reduced AMPK activity is associated with chronic inflammatory conditions such as obesity, type 2 diabetes, atherosclerosis and non-alcoholic fatty liver disease (NAFLD) where low AMPK activity exacerbates NF-κB signalling, cytokine production and immune cell infiltration (108, 109). Dysregulated AMPK signalling has also been implicated in autoimmune diseases such as rheumatoid arthritis and systemic lupus erythematosus, where it contributes to prolonged pro-inflammatory responses (110, 111). Chronic inflammation driven by low AMPK activity can also create a tumour-promoting environment in cancer (112, 113), while in neuroinflammatory diseases such as Alzheimer’s and Parkinson’s, dysregulated AMPK signalling contributes to neuronal damage and degeneration (114, 115).

### 2.7 cAMP/PKA

Similar to AMPK, the cAMP/PKA pathway has mainly anti-inflammatory and antioxidant properties. The anti-inflammatory mechanism is that the CREB–CREB-binding protein (CBP) complex formed by CREB phosphorylation by PKA can lead to the dissociation of the NF-κB–CBP complex, which blocks the action of NF-κB (116). PKA also inhibits activation of the pro-inflammatory ERK, AKT, STAT3 and NF-κB pathways through phosphorylation and inhibition of the TNFR1 receptor (117), so downregulation of PKA activity increases the strength of the coupling between inflammation and oxidative stress. The other inflammation-resolving pathway is via EPAC1/2 activation, which also inhibits NF-κB and GSK-3β (117). During the inflammatory phase, cAMP levels are reduced by an increase in the activity of PDE4 (which catalyses the breakdown of cAMP to AMP), which contributes to an increase in the severity of inflammation (118).

The cAMP/PKA pathway is a central messenger in the pro-resolving signalling pathways and is induced by, or induces, the production of other pro-resolving mediators as inflammation resolves (117, 118). For this reason, it is also an activator of the maturation of phagosomes, the main mechanism for removing extracellular debris after infection (119). The anti-inflammatory effect of cAMP is also mediated by the reduction of oxidative stress through an increase in the activity of sirtuin 3, which activates the production of antioxidant enzymes (120).

Adequate levels of cAMP are also essential for proper mitochondrial function. PKA phosphorylates complex I, leading to an increase in its activity and a decrease in electron leakage from this complex (121). However, the phosphorylation of complex IV by PKA leads to effective control of ATP production by properly functioning inhibition of ATP production under conditions of high ATP levels, whereas the dephosphorylation of complex IV leads to uncontrolled inhibition of this complex and a subsequent increase in electron leakage (122). Next, the phosphorylation of complex V by PKA stabilises the oligomers of this complex, improving ATP synthesis, whereas the lack of phosphorylation leads to the instability of the enzyme structure, lower ATP levels and inhibition of complex V by AIF1, shifting metabolism towards aerobic glycolysis (123, 124).

The effect of cAMP/PKA on autophagy is complex and depends on the metabolic context (125). On the one hand, autophagy is inhibited by the phosphorylation of Atg1/ULK1, a key initiator of autophagy (126), which limits the formation of autophagosomes; on the other hand, autophagy can be activated indirectly by activating EPAC and AMPK, further inhibiting mTOR, and by inhibiting NF-κB (117), which is an inhibitor of autophagy.

### 2.8 Pathway balance in chronic inflammations

The balance between the activity of the discussed pathways is one of the important elements regulating inflammation and preventing it from becoming chronic. The dominant current view of the development of chronic inflammation focuses on determining the role of individual pro- and anti-inflammatory pathway activities, which is, of course, an important aspect of the analysis. However, the present article focuses on the couplings between cytokines, ROS, NO, Cai^2+^, Nrf2 and autophagy. From the point of view of autophagy, some of the pathways discussed are stimulatory, some are inhibitory, and the action of others depends on the metabolic context. Thus, an imbalance in the ratio of these pathways towards excessive inhibition of autophagy may be one of the important causes of the transition of the system to chronic inflammation. According to the authors, future research on chronic inflammation should focus not only on determining the qualitative change in the activity of individual pathways but also on quantitatively comparing their activity and the overall effect on the elements analysed with a particular focus on autophagy.

## 3 Positive feedbacks regulating the inflammation

In the following subsections, the common relationships between the elements discussed, which drive inflammation mainly through the use of positive feedbacks, will be discussed step by step. From the point of view of control theory, positive feedback allows the equilibrium of the controlled system to move far from the initial state. However, it is a dangerous phenomenon for the controlled system because, if uncontrolled, it leads to the destruction of the system (the values of the positively coupled elements increase to infinity or the destruction of the system). For this reason, control mechanisms must be in place to prevent an uncontrolled and inappropriate increase in the controlled elements. In cellular metabolism, these are mainly the Nrf2/FOXO transcription factors and autophagy. Even small perturbations of such control mechanisms can lead to the formation of a new equilibrium state in which the concentrations and/or activities of the regulated elements are too high, which should be taken into account when analysing the relationships and planning future experiments and therapeutic strategies.

### 3.1 The positive coupling between the inflammation and ROS

The positive feedback between inflammatory cytokines and NOX-mediated ROS appears to be the main axis of the intracellular and extracellular response to various pathogens. The relationship is bidirectional, and many reciprocal relationships between ROS and various cytokines and interleukins have been described in different tissues and research conditions. On the one hand, NOXs are activated by several cytokines, and on the other hand, ROS generated by NOXs activate multiple immunological activators such as IL-6 and TNF-α (127).

#### 3.1.1 Role of NADPH oxidases

There are three main sources of ROS in the cell. The first one is the electron leakage from the cytochrome chain in the mitochondria (mito-ROS and mito-stress) (128), the second is the activity of NOXs (129), and the third is endoplasmic reticulum stress, where H_2_O_2_ is produced during protein folding as sulphur bonds are formed between cysteines. Other enzymes that produce ROS are xanthine oxidase, cytochrome P450, lipoxygenase and cyclooxygenase [103]. Externally, ROS are mainly produced by sources such as ionising radiation, UV light, xenobiotics and environmental pollutants [56].

The NADPH oxidase family is a group of seven enzymes (NOX1–5 and DUOX1–2) that produce O_2_^−^ and H_2_O_2_ to kill microorganisms and also perform various signalling functions. Different types are found in different tissues and parts of the cell and are regulated differently to perform different functions (129, 130). The immune response to pathogens consists largely of the production of H_2_O_2_ and O_2_^−^ by NOXs. The production of these molecules must increase to high levels during infection but must not exceed the limit of cell self-destruction. In healthy people, their activity should be limited to prevent the production of free radicals. In acute and chronic diseases, they are active to varying degrees (131–142).

#### 3.1.2 NOX → inflammation

**Figure 2:**
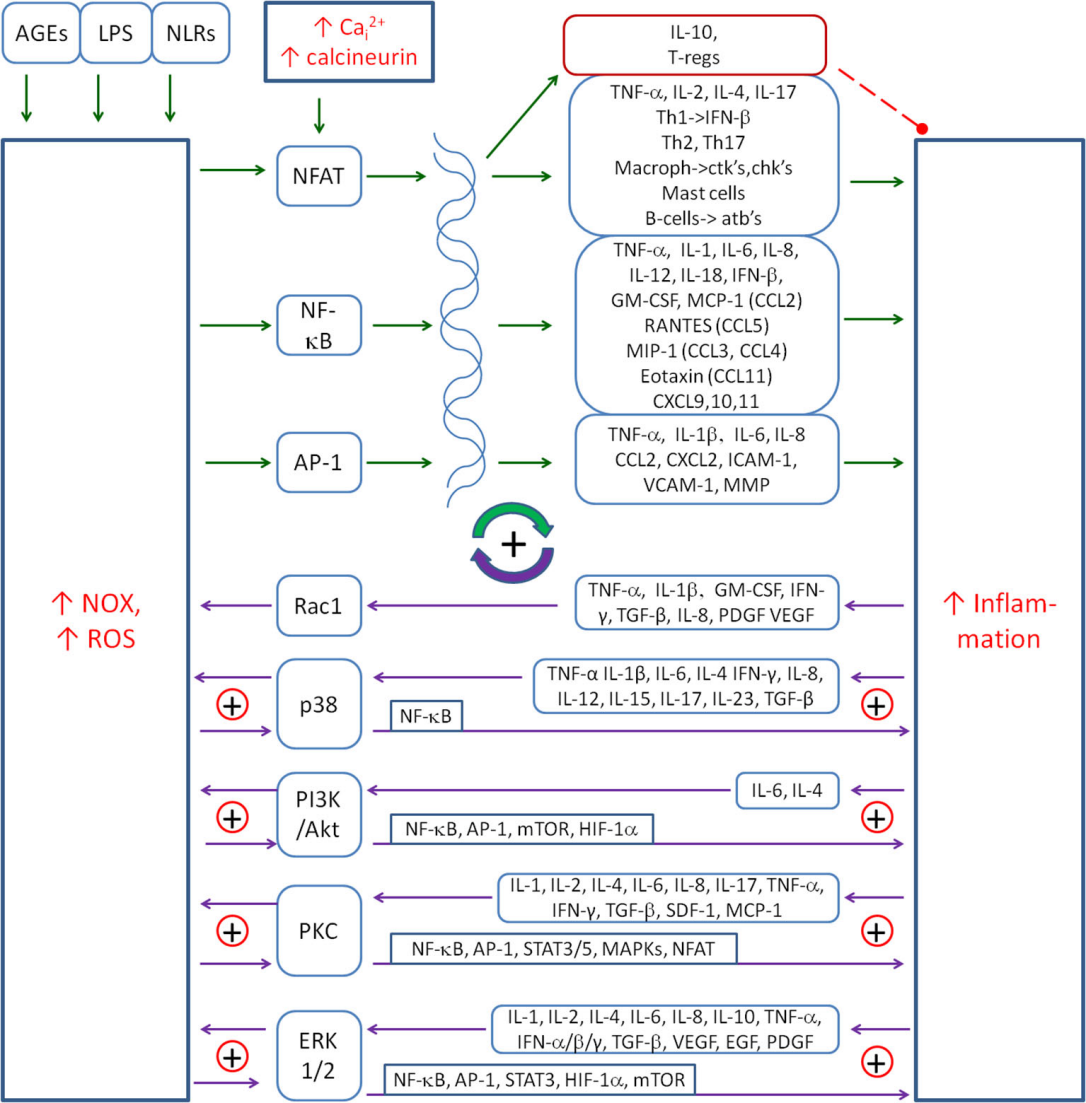
The main positive coupling between the inflammation and NOX-derived ROS that drive and amplify the inflammatory response. The main factors involved in this coupling are the transcription factors (NF-κB, AP-1 and NFAT), signalling pathways (p38, PI3K/Akt and ERK1/2) and the protein kinase C (PKC). Solid arrows, activation; red dashed line with •, inhibition; ⊕, the positive coupling between elements. NOX, NADPH oxidase; ROS, reactive oxygen species; NFAT, nuclear factor of activated T cells.

ROS produced by NOXs are involved in the activation of inflammation by activating pro-inflammatory transcription factors, such as the nuclear factor of activated T cells (NFAT), NF-κB and AP-1. **Figure 2** shows the major cytokines being involved in the inflammatory response upon the activation of these transcription factors. NF-κB is the major ROS-dependent transcription factor, which is responsible for cytokine and chemokine gene expression (143). It is subject to numerous regulations by several factors involved in the regulation of inflammation, such as NO, HIF-1α, Nrf2 and kinase pathways: p38, JNK, ERK1/2, AMPK and PI3K/Akt. It is also involved in multiple regulatory couplings. NFAT is the transcription factor that is mainly activated by increased intracellular calcium and the calcineurin pathway (144), but ROS are also mentioned as an activator of NFAT (145). AP-1 is the transcription factor that can be activated by various cell stress conditions, including ROS (146).

One of the ways that pathogens activate inflammation is through Nod-like receptors (NLRs), a family of intracellular sensors of microbial or danger-associated molecular patterns. Nod-like receptor X-1 (NLRX-1) is capable of activating NF-κB and inflammation, and ROS mediate this activation (147, 148). Overexpression of NLRX-1 can induce ROS production to levels similar to those induced by TNF-α, a well-characterised activator of ROS. In another study, ROS mediated the IL-6 secretion upon advanced glycation end products (AGE) or LPS induction, which was dependent on ROS-induced NF-κB activation (149). A similar mechanism of IL-6 production was presented in abdominal aortic aneurysm inflammation that was stimulated by angiotensin II-activated NOX-derived ROS production (150). In another study, cadmium-induced IL-6 production in trophoblast cells through ROS-dependent activation of ERK1/2 (151).

There is also ample evidence that ROS regulate the expression of many pro-inflammatory genes. For example, NOX-dependent ROS have been shown to induce the expression of transforming growth factor beta 1 (TGF-β1), angiotensin II, MCP-1 and plasminogen activator inhibitor-1 (152).

#### 3.1.3 Inflammation → NOX

Priming of NOXs occurs in response to a variety of cytokines such as TNF-α (153–155), IL-1β (156), IL-6 (157), IL-4 (158), IFN-γ (159), IL-8 (160), IL-12 (161), IL-15 (162), IL-17 (163), IL-23 (164) and TGF-β (165–169). There are several pathways that are used by cytokines to activate NOXs. The first one is Rac, which is the component protein of the NOX complex that is critical for the activation of NOX1 and NOX2. It is involved in many signals that increase NOX activation (129, 170). It is thought to act downstream of ROS production induced by cytokines such as TNF-α or interleukin-1β (171). TNF-α is also able to stimulate the membrane translocation of 47(phox) to activate NOX (143). Other cytokines involved in Rac-induced NOX activation are GM-CSF, TGF-β, PDGF and VEGF (172–176). AngII also activates NOX by Rac1 (170), which closes the positive loop between AngII and NOX.

Other signalling pathways used by cytokines to activate NOX are p38 MAPK, PI3K/Akt and protein kinase C (PKC). The PI3K pathway is used by IL-4 to activate NOX1 and NOX5L (177). IL-4 induced an intracellular calcium flux *via* the insulin receptor substrate (IRS)–PI3K–phospholipase Cγ (PLC-γ) pathway, which in turn induced PKC-dependent activation of NOX5. ROS in turn promoted IL-4 receptor activation through the oxidative inactivation of protein tyrosine phosphatase 1B (PTP1B), which physically associates with and deactivates the IL-4 receptor (158), closing a small positive loop between IL-4 and NOX5.

Many cytokines can activate the p38 pathway and thereby activate NOX (178). The most important are as follows: TNF-α (13), IL-1β (179, 180), IL-6 (181, 182), TGF-β (183), IL-34, IL-17 and GM-CSF. NOX can be activated by the p38 MAPK pathway, but p38 can also be activated by NOX-derived ROS, e.g. as a result of TNF-α (184), which closes the small positive loop between p38 and NOX. p38 MAPK can also activate inflammation by activating the pro-inflammatory NF-κB (185), which creates another positive feedback loop between p38 and inflammation. The whole forms a system of mainly positive feedback loops between cytokines, p38 and NOX. The details of the common couplings between NOX-derived ROS and the inflammatory cytokines are shown in **Figure 2**.

ERK1/2 is another mediator between ROS and inflammation. It activates transcription factors such as NF-κB (186), Elk-1 (186), AP-1 (31), Egr-1 (187), STAT3 (188) and HIF-1α (189), which induce the transcription of cytokines and/or NOXs, thereby further amplifying the inflammation–NOX coupling (190). In addition, ERK1/2 inhibits FOXO, which contributes to the reduction of the antioxidant response (191).

### 3.2 Nitric oxide and its couplings

#### 3.2.1 Role of iNOS

NO produced by iNOS has several functions in the cell, particularly in the context of the immune and inflammatory response. Its activity is important in the destruction of pathogens. NO and its derivatives damage key structures of pathogens, such as cell membranes, proteins, nucleic acids and enzymes, leading to their death (192). At the high concentrations reached during iNOS activity, NO can cause nitrosative stress and damage to cells and tissues, including cytotoxic effects on host cells. Reactive nitrogen species (e.g. peroxynitrite and ONOO^−^), formed when NO reacts with O_2_^−^, have potent oxidative effects and can damage lipids, proteins and DNA, contributing to chronic inflammation and tissue damage (192). It also acts on the mitochondria, contributing to increased free radical production and mitochondrial stress, which reduces energy production, lowers mitochondrial potential and promotes apoptosis (193). Although NO produced by iNOS does not play a major role in the regulation of vascular tone (this is mainly the responsibility of endothelial Nitric Oxide Synthase (eNOS)), its excess can indirectly affect blood vessels (194). High levels of NO can cause vasodilation, which contributes to increased blood flow at sites of inflammation, thereby increasing the access of immune cells to the infected or damaged area (194).

Unlike oxygen radicals, which are removed by anti-free radical enzymes, e.g. SOD, catalase and glutathione peroxidase, there are no specialised pathways to remove NO from the cell. The main pathway for its removal from the cell is diffusion, as NO readily crosses lipid membranes. In the bloodstream (195), NO reacts rapidly with the haemoglobin, which binds NO, converting it to nitrate (NO_3_^−^) (196). However, the peroxynitrite is neutralised by catalase (197), peroxiredoxin-3 (Prx-3) (198) or glutathione (199).

#### 3.2.2 Couplings between NO and inflammation

**Figure 3:**
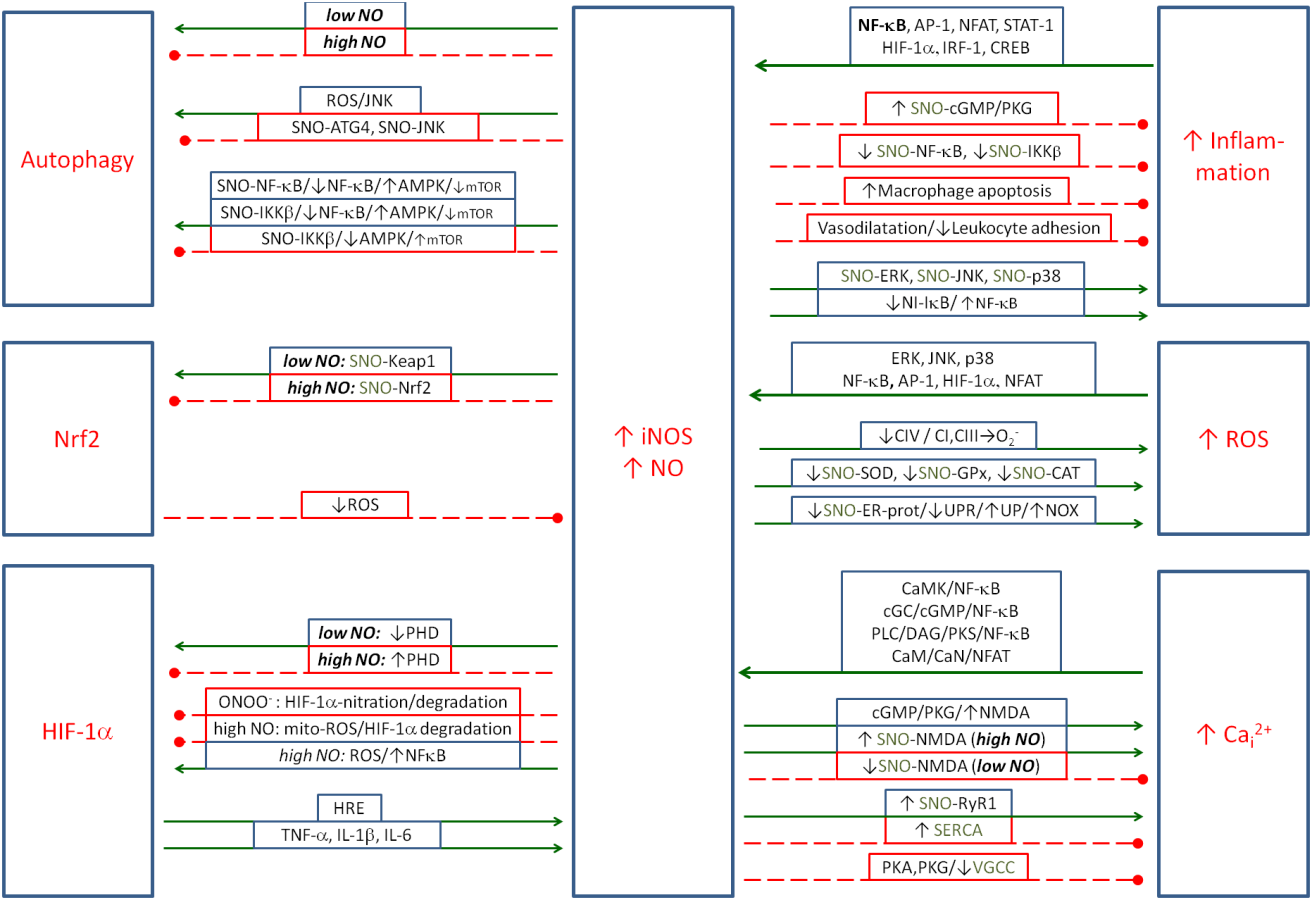
The metabolic couplings between the nitric oxide produced by iNOS and the elements of Positive Coupling System (inflammation/ROS/NO/HIF-1α/Ca_i_^2+^) and with regulatory elements: Nrf2 and autophagy. NO is the double-faced element, as in some cases it amplifies, and in some cases, it controls the inflammatory spiral. Solid arrows, activation; red dashed lines with •, inhibition. iNOS, inducible nitric oxide synthase; ROS, reactive oxygen species; NO, nitric oxide; HIF-1α, hypoxia-inducible factor 1-alpha.

NO is coupled mainly negatively with inflammation; however, some positive couplings have also been described (see **Figure 3**). Pro-inflammatory cytokines activate NO production, while NO inhibits the inflammatory process by several mechanisms. This action appears to be related to the strong oxidative effects of NO and ONOO^−^, which force the precise regulation of NO induction in the presence of intracellular pathogens.

Pro-inflammatory cytokines (such as IL-1, IL-6, IFN-γ and TNF-α) and pro-inflammatory transcription factor NF-κB play a key role in the induction of iNOS. The main mediating pathway is NF-κB, which leads to the transcription of the iNOS gene and an increase in NO production (200). The NF-κB pathway is crucial because it acts as the major transcription factor that activates iNOS expression in response to cytokine stimulation. Other pathways that also contribute to some extent to the activation of iNOS transcription are the AP-1 (activator protein-1) (201), NFAT (202), signal transducer and activator of transcription 1 (STAT-) (203), HIF-1α (204) and interferon regulatory factor 1 (IRF-1) (205). In the case of the cAMP/PKA/CREB pathway, CREB increases the expression of iNOS (206), but the increase in cAMP reduces the increased expression of iNOS and other inflammatory markers such as TNFα, IL-1β, IL-6, NF-κB, MMP-2 and MMP-9 in H9c2 cardiac cells, probably through different intermediate mechanisms (207). The above transcription factors cooperate in the induction of iNOS, and their cooperation allows the fine-tuning of iNOS expression levels to specific physiological conditions, such as inflammation, oxidative stress or hypoxia.

Conversely, NO is known to have mainly anti-inflammatory properties. NO acts mainly through cyclic GMP (cGMP) (208) and also directly through protein modifications (*S*-nitrosylation) (209), affecting the function of enzymes and regulatory proteins. cGMP activates protein kinase G (PKG), which leads to vascular smooth muscle relaxation, resulting in lower blood pressure (210). cGMP also inhibits platelet aggregation, which has an anticoagulant effect (211). cGMP also activates signalling pathways, such as PKG, which may have anti-inflammatory effects. PKG can inhibit leukocyte adhesion and activation, reduce the production of pro-inflammatory cytokines and decrease the reactivity of immune cells (212, 213). This effect is partly related to the inhibition of NF-κB by cGMP (214). NO can also inhibit NF-κB through *S*-nitrosylation of its p65 subunit, which blocks its ability to bind to DNA (215). NO can also nitrosylate and inhibit IKK-β (NF-κB activator) (215, 216). Conversely, nitrosylation of I-κB leads to NF-κB activation (217).

Another mechanism of NO’s anti-inflammatory action is the induction of apoptosis in activated macrophages and other immune cells under certain conditions, leading to a reduction in the inflammatory response. This mechanism prevents chronic inflammation by removing over-activated cells (218, 219). Another mechanism by which NO inhibits the inflammatory response is through its vasodilatory effect on vascular smooth muscle, which reduces leukocyte adhesion to the endothelium. Reduced leukocyte adhesion reduces the influx of inflammatory cells to the site of inflammation, thereby limiting the development of the inflammatory response (220, 221). Conversely, NO can activate inflammation by activating MAPK kinases (ERK, JNK and p38) through the induction of oxidative stress (e.g. through the production of reactive nitrogen species such as peroxynitrite) (222). The activation of MAPK kinases promotes the inflammatory response by affecting the expression of pro-inflammatory cytokines.

#### 3.2.3 NO + O_2_^−^ → ONOO^−^

The coupling between iNOS and NOX is mainly functional (223). NO and O_2_^−^ produced by these two enzymes generate the dangerous radical ONOO^−^, which greatly enhances the destructive effect of both radicals. In the experiment with primary co-cultures of rat cerebellar granule neurons and glia, the increase of NO or O_2_^−^ alone produced a benign toxic effect, but the co-activation of both enzymes produced a strong effect of neuronal death (223). The pro-surviving effect of NMDA (N-Methyl-D-Aspartate Receptor) inhibitor observed in this study suggests an important role of Ca_i_^2+^ in this process.

#### 3.2.4 NO → ROS

NO can enhance the production of oxygen free radicals in several ways. In the mitochondria, NO inhibits complex IV (cytochrome *c* oxidase) in the respiratory chain by competing with oxygen for the active site of this enzyme. Inhibition of complex IV leads to electron accumulation in the mitochondria, which increases electron leakage and O_2_^−^ generation by complexes I and III (224). Next, NO and its derivatives can nitrosylate key antioxidant enzymes such as superoxide dismutase (SOD), glutathione peroxidase (GPx) and catalase, reducing their activities and leading to the accumulation of ROS in cells (225). It can also nitrosylate certain proteins in the endoplasmic reticulum, leading to the accumulation of misfolded proteins, endoplasmic reticulum (ER) stress and subsequent NOX activation (226).

#### 3.2.5 ROS → iNOS

Conversely, ROS also activate iNOS in several ways. The main mechanism is through the activation of key transcription factors such as NF-κB, AP-1 and STAT1, which are essential for iNOS gene transcription (203, 227). ROS activate IKK, which phosphorylates IκB inhibitor, leading to NF-κB activation. ROS also activate MAPK kinases (ERK, JNK and p38), which phosphorylate and activate Jun and Fos proteins, which form the AP-1 complex. AP-1 binds to the iNOS promoter and promotes its expression. ROS can also enhance STAT1 activation by cytokines (e.g. IFN-γ), promoting its binding to the iNOS promoter (228). Pathways leading to the activation of the above-mentioned transcription factors are mainly MAPKs: p38, JNK and ERK1/2, which phosphorylate, among others, NF-κB and AP-1, promoting the transcription of pro-inflammatory cytokines, but also iNOS. Another indirect ROS mechanism is the activation of iNOS by an increase in Ca_i_^2+^.

#### 3.2.6 NO → Ca_i_^2+^

NO can modulate Ca_i_^2+^ levels by a variety of indirect mechanisms, and the final result depends on the physiological context. NO can nitrosylate RyR1 calcium channels, which increases its channel activity at lower O_2_ tension and increases Ca_i_^2+^ levels (229). This mechanism may contribute to the ER stress. To compensate for this, NO activates the sarco/endoplasmic reticulum Ca^2+^-ATPase (SERCA) calcium pump, which increases Ca^2+^ uptake into the ER (230).

NO also nitrosylates NMDA channels, inhibiting Ca^2+^ influx into the cell in physiological concentrations but increasing it in high concentrations (229, 231). NMDA receptor activation and Ca_i_^2+^ increase can also occur via the NO/cGMP/PKG pathway (232). Another way in which NO increases Ca_i_^2+^ is through the activation of transient receptor potential (TRP) channels by nitrosylation, which can increase Ca^2+^ influx into the cell (233). Conversely, NO can directly or indirectly inhibit voltage-gated calcium channels (VGCCs), thereby reducing Ca^2+^ influx into the cell through PKG and PKA signalling (234). It is generally accepted that excess NO and associated changes in Ca^2+^ concentration can lead to excitotoxicity in neurons, which is associated with neurodegenerative diseases (e.g. Alzheimer’s and Parkinson’s) (235), and that dysregulation of NO-Ca^2+^ signalling is implicated in inflammation and tissue damage (236).

#### 3.2.7 Ca_i_^2+^ → iNOS

Ca_i_^2+^ is a known activator of neuronal Nitric Oxide Synthase (nNOS) and eNOS but does not directly activate iNOS. However, Ca_i_^2+^ induces iNOS indirectly mainly through NFAT and NF-κB. High Ca_i_^2+^ activates the calmodulin/calcineurin/NFAT signalling pathway, which contributes to the transcription of iNOS (237). NF-κB is also the transcription factor, which is postulated to mediate the iNOS activation by Ca_i_^2+^ (238). Ca^2+^ ions can activate NF-κB via the calmodulin/calcineurin/CaMK (239, 240), soluble guanylyl cyclase (sGC)/cGMP (241) and PLC/diacylglycerol (DAG)/PKC pathways (242). Another pathway is the activation of ROS production in the mitochondria by mitochondrial Ca^2+^, which further leads to NF-κB activation.

#### 3.2.8 NO → autophagy

Nitric oxide plays a critical role in the regulation of autophagy, exerting both activating and inhibitory effects depending on its concentration, cellular context and signalling pathways involved. At physiological levels, NO generally promotes autophagy, helping to remove damaged organelles and maintain cellular homeostasis. However, under pathological conditions such as chronic inflammation or oxidative stress, excessive NO acts as an inhibitor, exacerbating cellular damage.

NO activates autophagy primarily through the AMPK–mTOR pathway (243). In response to metabolic stress, NO can induce energy stress by increasing the AMP-to-ATP ratio, which activates AMPK. Activated AMPK inhibits mTOR, a key suppressor of autophagy, thereby initiating the autophagosomal process via the activation of the ULK1 complex. In addition, NO can increase the production of ROS, which activate pathways such as JNK, further enhancing autophagy in response to oxidative stress.

Conversely, NO can inhibit autophagy under certain conditions, particularly when present in excess. One important mechanism is the *S*-nitrosylation of key autophagosomal proteins such as ATG4, which impairs their function and blocks the elongation of the autophagosomal membrane (244). Another pathway is the nitrosylation and deactivation of JNK1, which is an important autophagy activator (see **Figure 1**) (245). NO also nitrosylates (inhibits) IKKβ, which reduces AMPK phosphorylation (activation). This leads to the activation of mTORC1 and inhibition of autophagy. *S*-Nitrosylation of IKKβ is thought to be a negative feedback mechanism during inflammation to prevent excessive activation of NF-κB, thereby protecting tissues from chronic inflammation or damage (215, 216). NO can also activate sGC, leading to increased levels of cGMP and potential mTOR activation, thereby suppressing autophagy (246, 247).

Another pathway of NO activity is via TSC1/2 (tuberous sclerosis complex), a known inhibitor of mTOR and activator of autophagy. IKKβ, Akt and ERK1/2 inhibit this complex, and AMPK activates it, contributing to the regulation of autophagy (245). Finally, in the case of chronic inflammation, the chronic NF-κB activation is a factor that inhibits AMPK and activates mTOR, thereby contributing to autophagy inhibition, which seems to be an important element of the overall autophagy inhibition in chronic information observed under several conditions. The dual role of NO in autophagy highlights its dependence on the metabolic context. Excess of NO and over-nitrosylation seems to be an important element driving the entry of inflammation into a chronic state when the summary effect on autophagy is the inhibitory one.

#### 3.2.9 NO and ONOO^−^ → Nrf2

Nrf2 is the central antioxidant transcription factor, which is responsible for inhibiting excessive oxidative stress and inflammation. Its coupling with iNOS/NO is NO concentration dependent. Nrf2 is regulated by Kelch-like ECH-associated protein 1 (Keap1). It contains many reactive cysteine residues (e.g. Cys151, Cys273 and Cys288) that are susceptible to *S*-nitrosylation, leading to a reduction in its ability to bind Nrf2 (248). This leads to Nrf2 release and translocation to the nucleus. In addition, 8-nitro-cGMP-dependent *S*-guanylation of Keap1 leads to Nrf2 activation, with the concomitant expression of the targeted antioxidant enzymes that play a role in signalling under oxidative stress conditions (249, 250).

At low concentrations, NO activates Nrf2 by nitrosylating Keap1 (248). The other pathway of Nrf2 activation is the activation of PKC-α by NO in kidney cells (251) or PKC-ε (252), which phosphorylate Nrf2 and have the same effect (253). Among the PKC isoforms, PKC-δ plays a predominant role in phosphorylating Nrf2, particularly at serine 40, promoting its dissociation from Keap1 (254). ONOO^−^ at physiological concentrations also has the ability to activate Nrf2 (255–257). The intermediate pathway for this effect may be PI3K/Akt (258).

ONOO^−^, as a dangerous radical, is also known for its destructive effects on the activities of various enzymes. In contrast to the activation of Nrf2 by NO and ONOO^−^, inhibitory effects of ONOO^−^ on Nrf2-induced enzymes have been demonstrated, e.g. HO-1 (259), catalase (260), Mn-SOD (261), peroxiredoxin II E (262), glutathione peroxidase (263) and thioredoxin reductase (263). It can therefore be concluded that exceeding a certain level of ONOO^−^ concentration in the cell leads to a breakdown of the antioxidant barrier, which is probably a part of the molecular pathology in various diseases.

#### 3.2.10 NO → mitochondria

NO is a reversible inhibitor of mitochondrial complex IV. This inhibition, although readily reversible, can have profound consequences for the cell (264). Inhibition of the cytochrome chain at the level of complex IV can lead to the production of superoxide due to the electron leakage from complexes I and III, which in turn leads to the production of ONOO^−^. ONOO^−^ is the irreversible inhibitor of complex IV, which enhances the regulatory effect of NO (197). ONOO^−^ can also irreversibly damage many of the mitochondrial enzymes including aconitase, NADH/co-Q reductase, quinol/cytochrome *c* reductase, succinate dehydrogenase and the ATP synthetase (265, 266). The resulting collapse of the mitochondrial membrane potential can open the mitochondrial permeability transition pores (mPTPs), release the cytochrome *c* into the cytoplasm and trigger apoptotic cell death.

#### 3.2.11 NO—summary

The effect of nitric oxide is both activating and inhibitory in all the relationships discussed. It exhibits outstanding non-linear properties, contributing predominantly to the maintenance of homeostasis at low physiological concentrations and leading to metabolic collapse at high concentrations. This implies the need for a detailed in-depth analysis of the role of NO depending on the metabolic context and, above all, NO concentration. 

### 3.3 Calcium stress

**Figure 4:**
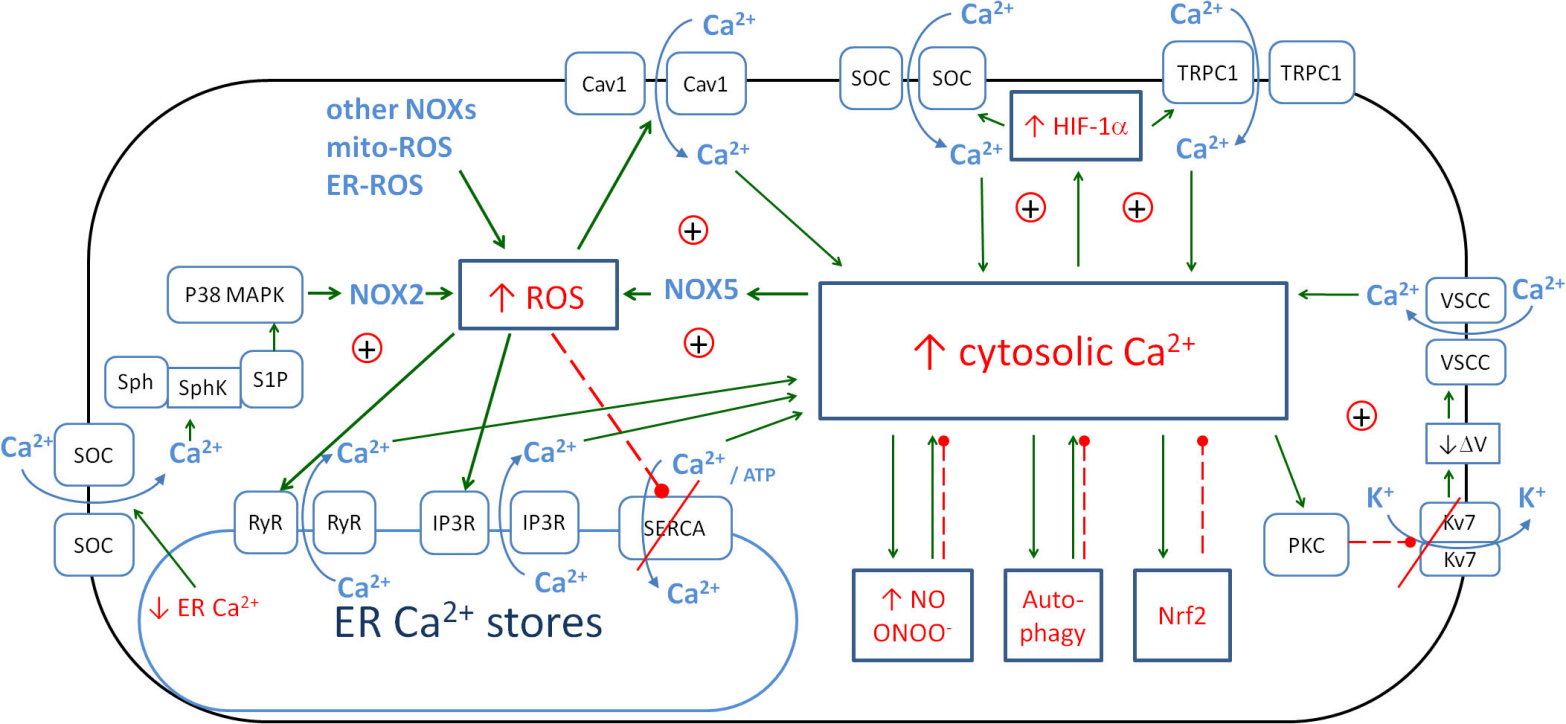
The metabolic links between cytosolic Ca^2+^, endoplasmic Ca^2+^, ROS and HIF-1α create the positive couplings that enhance the anti-pathogen response. The details of the couplings with iNOS/NO are in **Figure 3**, with autophagy, and Nrf2 in **Figure 6**. Solid arrows, activation; red dashed lines with •, inhibition; ⊕, positive coupling between elements; red line crossing out, inhibition of the channel. ROS, reactive oxygen species; HIF-1α, hypoxia-inducible factor 1-alpha; iNOS, inducible nitric oxide synthase; NO, nitric oxide; Nrf2, nuclear factor erythroid 2-related factor 2.

Ca_i_^2+^ is associated with inflammation and oxidative stress through a number of couplings and is also involved in enhancing the immune response. **Figure 4** shows the detailed relationships between intracellular and endoplasmic calcium, ROS and HIF-1α. The main influence of Ca_i_^2+^ is through its positive loop with the NOX-mediated ROS.

Calcium is an important intracellular messenger molecule that is significantly involved in antimicrobial defence. Normal resting Ca_i_^2+^ levels in the cytoplasm of most cells are typically very low, ranging from approximately 50 to 100 nM (267). Calcium ions are the second messengers that cause the activation of multiple downstream proteins. The pumping of calcium ions out of the cell requires a large amount of energy, as the divalent ions must be pumped out against a high electrical (approximately −70 to −90 mV) and high concentration gradient (on the order of 1,000-fold) (268). The pumping out of one Ca_i_^2+^ ion requires one molecule of ATP so that the reduction of energy production in the mitochondria, e.g. by HIF-1α-mediated PDH inhibition, can lead to a further increase of Ca_i_^2+^ in the cytoplasm and mitochondria (269, 270). However, Ca_i_^2+^ ions contribute to the opening of mPTPs, leading to a decrease in the mitochondrial membrane potential Ψ_m_ (271), which worsens the conditions for energy production and facilitates apoptosis.

#### 3.3.1 ER stress and Ca^2+^

The increase in cytoplasmic Ca_i_^2+^ is the main form of calcium stress, but the second important type of calcium stress is associated with the ER. The two are interrelated, as increases in Ca_i_^2+^ are often associated with decreases or increases in Ca_ER_^2+^. The ER is a major store of Ca^2+^ and typically maintains a much higher concentration than the cytosol. The Ca_ER_^2+^ levels are typically in the range of 300 to 800 µM (272, 273), which is essential for proper protein folding and serves as a reservoir that can release Ca^2+^ when needed for signalling purposes. Many of the chaperones and enzymes involved in protein folding in the ER, such as calnexin and calreticulin, require adequate Ca_ER_^2+^ for their structural stability and functional activity (274). During ER stress, calcium homeostasis is often disrupted (275–277). The ER can release Ca^2+^ into the cytosol, leading to an increase in cytosolic Ca^2+^ concentration. This release can activate various signalling pathways, including those that lead to cellular responses such as apoptosis or autophagy if the stress is severe or prolonged. Conversely, prolonged ER stress can lead to depleted ER calcium levels, which can affect protein folding and other ER functions, leading to the accumulation of misfolded proteins (277–280).

Oxidative protein folding refers to the process by which proteins acquire their proper structure through the formation of disulfide bonds between cysteine residues in the proteins. This reaction is mediated by a number of protein disulfide isomerases and oxidoreductases such as ER oxidoreductin 1 (Ero1) and protein disulfide isomerase (PDI) (281, 282). Changes in Ca_ER_^2+^ levels can affect the activity of Ero1 and PDI, which in turn alters the production of H_2_O_2_, a by-product of disulfide bond formation. At low Ca^2+^ concentrations in the ER, Ero1 activity is reduced, which reduces H_2_O_2_ production. This results in reduced oxidative stress in the ER but may also affect protein folding. At high concentrations of Ca^2+^ in the ER, Ero1 activity increases, which increases H_2_O_2_ production (283). The ER contains specific glutathione peroxidases, such as GPx7 and GPx8, which are critical for reducing peroxide levels (284, 285). These enzymes use glutathione (GSH) as a substrate to convert H_2_O_2_ to water, directly neutralising it. Peroxiredoxins, such as Prx-4, are another group of ER-localised antioxidants that help reduce peroxides (286, 287).

Inefficient removal of H_2_O_2_ in the ER leads to oxidative stress, which can damage the calcium pumps (such as SERCA). This impairs its ability to pump Ca^2+^ into the ER, resulting in reduced ER calcium levels. A decrease in ER calcium due to oxidative stress impairs the activity of the calcium-dependent enzymes, leading to the accumulation of misfolded proteins in the ER, which initiates an unfolded protein response (UPR). However, prolonged oxidative stress and impaired calcium levels can compromise the effectiveness of these responses, leading to chronic ER stress and a vicious cycle of low Ca_ER_^2+^ and high H_2_O_2_.

Chronic ER stress has been implicated in a variety of diseases, reflecting its fundamental role in cell function and survival. It has been implicated in neurodegenerative diseases [Alzheimer’s (276), Parkinson’s and Huntington’s (279)], diabetes (both type 1 and 2), cardiovascular disease (atherosclerosis and heart failure) (274), cancer, obesity, inflammatory bowel disease and liver disease (275). In summary, there is a similarity between the ER and mitochondria in that both organelles produce ROS as a by-product and have the mechanisms to reduce the functionally relevant oxidative stress.

#### 3.3.2 Ca_i_^2+^ ↔ NOX

One of the major pathways for Ca_i_^2+^ elevation is oxidative stress and the regulation of Ca_i_^2+^ signalling by NOX enzymes (288). Three pathways for ROS-induced Ca_i_^2+^ elevation have been described. The first is the Ca^2+^ influx via the opening of voltage-gated L-type Ca^2+^ channels (Cav1) (289, 290). The second pathway is Ca^2+^ release from intracellular stores (291, 292), for example, via the ryanodine receptor (RyR) family, which have reactive cysteine residues that are highly sensitive to oxidation by ROS (293). ROS also act on another type of Ca^2+^ release channel, namely, the inositol 1,4,5-trisphosphate receptor (IP3R) family (294, 295). Finally, ROS can modulate the activity of Ca-ATPase pumps (SERCA) that remove Ca_i_^2+^ from the cytoplasm (292, 296, 297) in a bimodal manner. The mechanism of ROS-dependent Ca_i_^2+^ pump activation involves the mechanisms of ROS-dependent *S*-glutathiolation of protein cysteines mediated by the interaction of glutathione and peroxynitrite (296). This activation of the Ca^2+^ pump by *S*-glutathiolation occurs at low ROS concentrations. Increased oxidative stress leads to the irreversible oxidation of thiols and thus to enzyme inhibition (292). In this way, the negative regulatory coupling becomes the positive one contributing to the increase in oxidative stress and inflammation. The restoration of the negative regulatory coupling may be an important part of the treatment of many pathological conditions.

The increase in intracellular calcium concentration is induced by NOX-derived ROS, but the opposite regulation also takes place. An increase in cellular calcium levels is associated with many metabolic effects. One of these is the activation of NADPH oxidases (129, 298, 299). The details of the stimulatory effect of calcium ions on the activity of individual NOX enzymes have been described in reviews (129, 300, 301). In brief, the activating effect of calcium ions on NOX can be direct and indirect. The direct effect is described in relation to NOX5. The expression of NOX5 is restricted to a few tissues, although it is found in human vascular smooth muscle cells (VSMCs), endothelial cells and whole vessels—important tissues in the development of COVID-19 pathology. Calcium induces the binding of the N-terminal domain of NOX5 to its dehydrogenase domain, thereby relieving autoinhibition. In microvascular endothelial cells, NOX5 expression is also increased by endothelin-1 and AngII and mediates the activation of ERK1/2 (302). Another association of NOX5 with endothelial cells was shown by Guzik et al. (303). They showed that NOX5 expression is higher in human coronary arteries with coronary artery disease than in those without the disease. This increased NOX5 expression was accompanied by a sevenfold increase in activity.

In the case of NOX2, the role of Ca_i_^2+^ is indirect. In non-excitable cells, Ca^2+^ influx is essentially mediated by store-operated calcium entry (SOCE), a complex mechanism in which the depletion of intracellular Ca^2+^ stores from the ER leads to Ca^2+^ entry through Ca^2+^ store-operated calcium channels (SOCs) at the plasma membrane. Extracellular Ca^2+^ entry is known to be involved in NOX2 activation. Schenten et al. (304) showed that sphingosine kinase (SphK)-regulated NOX2 activation depends on the depletion of intracellular Ca^2+^ stores. Their results define a pathway leading to NOX2 activation, in which store depletion-dependent SphK activation induces p38 MAPK-mediated S100A8/A9 translocation. S100A8/A9, also known as calprotectin, functions as a damage-associated molecular pattern (DAMP) that activates various signalling pathways, including those involving NOX2 as a defence mechanism against pathogens (304). However, its role in inflammation and oxidative stress is more complex, as some anti-inflammatory properties have also been described (305).

The important pathway for Ca_i_^2+^ elevation is the activation of phospholipase C (PLC), which leads to Ca_i_^2+^ elevation and further stimulation of PKC. PKC can be activated by increases in intracellular calcium, but it can also contribute to increases in Ca_i_^2+^ levels. Haick et al. (306) showed that PKC activation leads to the suppression of Kv7 (family of voltage-gated potassium channels) currents, membrane depolarisation and Ca^2+^ influx through L-type voltage-sensitive calcium channels (VSCCs) (306). Thus, a positive loop between the PKC activity and Ca_i_^2+^ concentration can be observed, which is switched on by PLC activation. Several mechanisms can activate PLC. GPCRs can activate PLC-β, and RTKs can activate PLC-γ. High concentrations of calcium ions can activate the PLC-δ isoform. PLC can also be indirectly activated by ROS and ONOO^−^.

#### 3.3.3 Inflammation → Ca_i_^2+^

The binding of cytokines to receptors activates signalling pathways, such as the MAPK, PLC pathways or NF-κB, which can influence the increase in intracellular calcium ion concentration through various pathways. The activation of PLC leads to the breakdown of phosphatidylinositol-4,5-bisphosphate (PIP2) into inositol-1,4,5-triphosphate (IP3) and DAG. IP3 binds to IP3 receptors in the ER, causing the release of Ca^2+^ ions from the ER into the cytoplasm (307). In turn, DAG activates transient receptor potential canonical (TRPC) channels, leading to an influx of Ca^2+^ ions from the extracellular space (308). Cytokines can also activate SOCE channels for Ca^2+^ to facilitate the influx of ions from the outside after the depletion of calcium stores in the ER (309).

#### 3.3.4 Ca_i_^2+^ → inflammation

Calcium ions induce inflammation mainly by increasing the production of free radicals, which activate the inflammatory cascades described above. However, there are several pathways that activate inflammation without the mediation of ROS. Increased Ca_i_^2+^ concentration is a signal that activates the NLRP3 inflammasome, which catalyses the conversion of pro-IL-1β to active IL-1β (310). The activation of the inflammasome further increases cytokine release and enhances Ca_i_^2+^ mobilisation, creating a positive feedback loop between inflammation and Ca_i_^2+^. Ca_i_^2+^ also enhances the transcription of pro-inflammatory genes through the activation of factors such as NF-κB (311), which then increases the activity of iNOS, leading to NO production.

#### 3.3.5 Ca^2+^ → autophagy

Ca_i_^2+^ is an important regulator of autophagy. Under normal conditions, Ca_i_^2+^ activates the autophagy in healthy cells mainly by activating calcium/calmodulin-dependent kinase kinases (CaMKKs), in particular CaMKKβ (312–315). This kinase activates AMPK (316), which in turn inhibits mTOR, a key negative regulator of autophagy. In addition, calcineurin activated by high intracellular Ca^2+^ levels can dephosphorylate transcription factor EB (TFEB), a master regulator of lysosomal biogenesis and autophagy genes (317–319). Dephosphorylated TFEB translocates to the nucleus and enhances the transcription of autophagy-related genes, thereby promoting autophagy​​.

ER stress is one of the important conditions that increase Ca_i_^2+^ concentration by its release from Ca^2+^ stores and initiates the above-mentioned pathways (320). However, autophagy can be activated by the inter-organelle transfer of Ca^2+^ from the ER to the mitochondria via the mitochondria-associated membrane (MAM) (321). Key proteins located at the MAM include the IP3Rs on the ER side and the voltage-dependent anion channels (VDACs) on the mitochondrial outer membrane, which are connected by the chaperone protein glucose-regulated protein 75 (GRP75)​​ (322). Ca^2+^ is released from the ER via IP3Rs into the MAM space and is then rapidly taken up by the mitochondria via VDACs and the mitochondrial Ca^2+^ uniporter (MCU). This Ca^2+^ transfer is essential for the activation of mitochondrial enzymes involved in energy production, such as those in the Krebs cycle​​. The transfer of Ca^2+^ from the ER to the mitochondria can affect mitochondrial dynamics and promote mitochondrial fission, which is often associated with the initiation of autophagy (323).

However, autophagy can modify Ca_i_^2+^ levels in a bimodal manner. Autophagy can increase Ca_i_^2+^ levels by depleting intracellular Ca^2+^ stores such as the ER or by affecting the membranes of lysosomes where Ca^2+^ channels are located (324). The depletion of these stores may lead to a transient increase in cytosolic Ca^2+^, which may produce feedback to promote further autophagic activity. Conversely, although autophagy can be stimulated by Ca^2+^, the process itself tends to balance Ca^2+^ levels within the cell. Excessive autophagy can lead to the excessive depletion of Ca^2+^ stores, lowering intracellular Ca^2+^ levels to a point where autophagic activity is reduced, thus preventing cellular damage from excessive autophagy (325).

In conclusion, intracellular calcium is mainly positively associated with inflammation and oxidative stress, but some described negative couplings at physiological concentrations act as regulators of excessive Ca_i_^2+^ increase.

### 3.4 Activation of HIF-1α pathway

**Figure 5:**
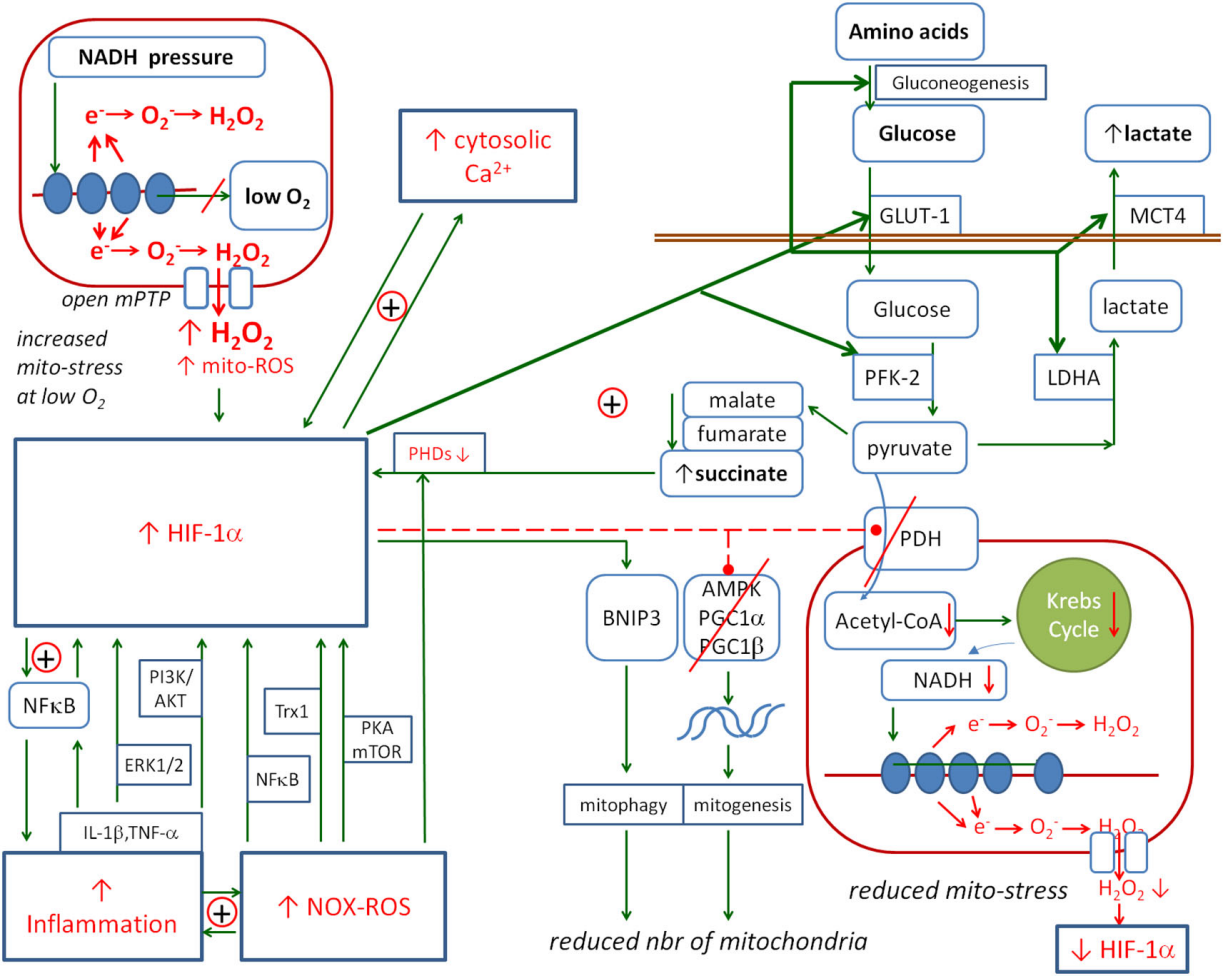
The metabolic links between HIF-1α and mitochondria. HIF-1α is activated by inflammation, ROS and Ca_i_^2+^. Its activation promotes the pathways that prevent mitochondrial ROS production: HIF-1α activates the anaerobic glycolysis and reduces the number of mitochondria. The positive loop between HIF-1α and succinate enhances the protective effect on mitochondria. Solid arrows, activation; red dashed lines with •, inhibition; ⊕, positive coupling between elements; red line crossing out, inhibition of the channel or pathway. HIF-1α, hypoxia-inducible factor 1-alpha; ROS, reactive oxygen species.

The next important player in the inflammatory response is HIF-1α. **Figure 5** shows the detailed relationships between this factor and inflammation, ROS, NO, Ca_i_^2+^ and mitochondria. Under normal conditions, HIF-1α levels increase during the hypoxia state. In this case, it has a protective function against the overproduction of free radicals in the mitochondria during hypoxia or hypoxia/reperfusion by reducing pyruvate entry into the mitochondria and NADH “pressure” on the cytochrome chain, thereby reducing mito-stress (128). Its protective effect has been demonstrated, for example, in myocardial infarction, where it activates the anaerobic glycolysis enzyme 6-phosphofructo-2-kinase/fructose-2,6-bisphosphatase 2 (PFK-2/FBPase-2) and supports anaerobic ATP production (326). In another study, HIF-1α-deficient mice showed greater intestinal barrier disruption and a more severe course of colitis (327).

HIF-1α is degraded by HIF prolyl hydroxylase domains (PHDs), and inhibition of PHDs results in the stabilisation of HIF-1α. However, Factor Inhibiting HIF (FIH) is an oxygen-dependent enzyme that causes the hydroxylation of HIF-1α, which in turn inhibits the interaction of the HIF-1α subunit with CBP/p300 (HIF-1α co-activator) (328–330). This interaction is required for the transcription of HIF-dependent genes. Therefore, FIH provides a mechanism for reducing the transcriptional activity of the HIFs in normoxia.

HIF-1α can be stabilised by the inhibitory effect of succinate (SC) on the HIF prolyl hydroxylases (331–335). Increased levels of Krebs cycle molecules in the cytoplasm such as oxaloacetate, fumarate, malate and succinate may be the effect of pyruvate dehydrogenase (PDH) inhibition. Thus, a possible positive feedback loop may be observed in which HIF-1α inhibits PDH, inhibited PDH increases the concentration of succinate, and increased succinate stabilises HIF-1α.

#### 3.4.1 NOX/ROS → HIF-1α

ROS generated by NOXs are important activators and stabilisers of HIF-1α. They inactivate PHDs, leading to an increase in the stability of the HIF-1α protein. ROS also activate PKA and mTOR, which phosphorylate HIF-1α, increasing its stability and leading to its accumulation in the cell (336, 337). ROS also upregulate the expression of thioredoxin 1 (Trx1), which increases the transcriptional activity of HIF-1α (338). However, ROS-mediated HIF-1α induction also occurs at the transcriptional level, and it is dependent on NF-κB—a major transcription factor for inflammatory cytokines (336). Finally, ROS generated by exogenous H_2_O_2_ or by a NOX4 transcriptionally induce HIF-1α via the NF-κB binding site in the HIF-1α promoter (339).

#### 3.4.2 HIF-1α → NOX

The reverse relationship, i.e. the activation of NOX by HIF-1α under certain conditions, has also been reported, albeit in small numbers. André-Lévigne et al. (340) reported that HIF-1α activates the transcription of NOX4 in the context of wound repair activation. In another article, Diebold et al. reported that the transcription of NOX2 is activated by HIF-1α in the context of the urotensin-II-activated angiogenesis (341, 342).

#### 3.4.3 HIF-1α ↔ NF-κB-positive coupling

HIF-1α activates the inflammation mainly through NF-κB (343), which is crucial for inducing the production of pro-inflammatory cytokines, e.g. TNF-α and IL-6. Conversely, NF-κB has been shown to contribute to increased *Hif1a* mRNA transcription under hypoxic conditions (344, 345). BelAiba et al. (344) showed that the expression of the NF-κB p50 and p65 subunits increased HIF-1α mRNA levels, while blocking NF-κB with the NF-κB inhibitor attenuated the induction of HIF-1α mRNA by hypoxia. Reporter gene assays revealed the presence of an NF-κB site in the HIF-1α promoter, and mutation of this site abolished HIF-1α induction by hypoxia. Gel shift analysis and chromatin immunoprecipitation confirmed the binding of the p50 and p65 subunits of NF-κB to the HIF-1α promoter under hypoxia. In another study, Frede showed that LPS increased HIF-1α mRNA expression through the activation of an NF-κB site in the promoter of the HIF-1α gene, and hypoxia post-translationally stabilised HIF-1α protein (345).

#### 3.4.4 Cytokines → HIF-1α

The previous subsection discussed the coupling between HIF-1α and the pro-inflammatory NF-κB. The direct effect of cytokines on HIF-1α has also been observed. Malkov et al. (346) discussed the influence of two cytokines, TNF-α and IL-1β, on HIF-1α. Both can activate HIF-1α via both the NF-κB and PI3K/Akt pathways (347–349). The activation of these pathways results in increased HIF-1α protein synthesis and stabilisation under normoxic conditions. TNF-α stimulation leads to the activation of NF-κB, which can bind to the HIF-1α promoter and enhance HIF-1α transcription. The PI3K/Akt pathway stabilises HIF-1α through the post-translational modification and inhibits its degradation. This pathway is used by both TNF-α and IL-1β. In addition, IL-1β uses the ERK1/2 pathway to increase HIF-1α activity (350–352). In another paper, Zhang et al. (353) showed that the synthesis of HIF-1α was upregulated by IL-1β in hepatocellular carcinoma cells via cyclooxygenase-2. Their findings revealed a HIF-1α/IL-1β signalling loop between cancer cells and tumour-associated macrophages in a hypoxic microenvironment, leading to epithelial–mesenchymal transition and cancer cell metastasis.

The above observations close a positive loop coupling between HIF-1α and inflammation that contributes to the amplification of inflammation, especially in the case of hypoxia, which is an important element of the local environment in viral or bacterial infections (354).

#### 3.4.5 Ca_i_^2+^ ↔ HIF-1α

HIF-1α is the master regulator of hypoxic transcriptional responses and controls the transcription of several calcium modulators, which can lead to the remodelling of the calcium signalling. Translational regulation of HIF-1α is estimated to account for up to 50% of the increased HIF-1α protein levels under hypoxia, and this process is promoted by calcium signalling (355, 356). The interplay between Ca_i_^2+^ and HIF-1α and their positive feedback in cancer cells has been reviewed by Azimi (357). The Ca_i_^2+^ modulatory proteins being involved in the direct positive feedback with HIF-1α are the transient receptor potential C1 calcium channel (TRPC1) and stroma interaction molecule-1 (STIM1; Ca_ER_^2+^ sensor). HIF-1α activates their transcription. Conversely, TRPC1 regulates the translation of HIF-1α (358), and STIM1 promotes HIF-1α transcription and accumulation (359). In addition to TRPC1 and STIM1, other proteins that have been described to increase the activity of HIF-1α include TRPC5, TRPC6, TRPM8 and TRPM2. TRPC5 regulates the HIF-1α expression and its nuclear translocation in breast cancer cells (360), TRPC6 controls the hydroxylation and stability of HIF-1α in glioma (361), TRPM8 promotes HIF-1α levels by suppressing RAK1-mediated HIF-1α ubiquitination in prostate cancer (362), and TRPM2 increases HIF-1α levels by increasing transcription and decreasing degradation in neuroblastoma (363). However, Vestra et al. showed that HIF-1α expression in LPS-stimulated THP-1 macrophages could be blocked by the CaMKII (calcium/calmodulin-dependent protein kinase II) inhibitor KN93, suggesting a role for this complex in HIF-1α activation (364). Summing up, the interplay between HIF-1α and Ca_i_^2+^ is described mainly in cancer conditions. Further research is required to explain if similar relations take place in the case of chronic inflammation.

#### 3.4.6 HIF-1α vs. mitochondria

The mitochondria are the ATP factories and the elements of the cell that are extremely sensitive to oxygen deprivation, so various metabolic disorders in the mitochondria trigger the activation of HIF-1α to activate protective mechanisms against the effects of these disorders. The main detrimental element associated with energy production is the production of free radicals due to electron leakage from the cytochrome chain, known as mito-stress. In contrast to the positive coupling of HIF-1α with cytokines, NOX and Ca_i_^2+^, the coupling with mito-stress is mainly negative. The mechanisms linking mitochondrial metabolism to HIF-1α are compensatory, preventing mitochondrial damage or facilitating mitochondrial survival under stress conditions. The mechanisms observed are aimed at reducing the flow of electrons through the cytochrome chain in order to reduce their leakage under conditions of oxygen deprivation. The positive feedback between HIF-1α and succinate/fumarate acts as the amplifier of this inhibitory relationship. Succinate and fumarate contribute to the stabilisation of HIF-1α through their inhibitory effect on PHD, while the activation of HIF-1α leads to an increase in their concentration in the mitochondria by blocking PDH, activating glycolysis (GLL), gluconeogenesis (GNG) and the glucose transporter GLUT-1, all of which contribute to an increase in succinate in the cell.

#### 3.4.7 HIF-1α → mitochondria

There are several ways in which HIF-1α affects mitochondrial activity. The general effect is to inhibit their work. HIF-1α inhibits the pyruvate dehydrogenase complex, thereby reducing the entry of pyruvate into the mitochondria and reducing NADH production. At the same time, it positively regulates lactate dehydrogenase A (LDHA) expression and promotes the conversion of pyruvate to lactate (365) and further removal of lactate from the cell by upregulating the monocarboxylate transporter 4 (MCT4) (366). HIF-1α also reduces the number of mitochondria by inhibiting the PPAR co-activator-1 (PGC-1) family members PGC-1α and PGC-1β (peroxisome proliferator-activated receptor gamma co-activator 1-alpha/beta), which are the essential transcription factors for mitochondrial biogenesis (367). Lu et al. (368) showed that in an *in vitro* mouse genioglossus myoblast model, HIF-1α inhibited AMPK (a low-energy sensor that activates ATP production) under hypoxic conditions, which inhibited mitochondrial biogenesis, decreased the PGC-1α levels and increased apoptosis.

In addition, the population of the mitochondria is reduced by the activation of mitophagy. The known HIF-1α target gene is Bcl-2/adenovirus E1B 19 kDa-interacting protein 3 (BNIP3), which is involved in autophagy and is able to reduce the mitochondrial population (369). This mechanism requires the HIF-1-dependent expression of BNIP3 and the constitutive expression of Beclin-1 and Atg5. The effect of this reduction is also to decrease mito-ROS production (370). HIF-1α activation also causes mitochondrial fission in human models of pulmonary arterial hypertension, which is supported by the phosphorylation of dynamin-related protein 1 (DRP1), and this process may also be associated with the ability to reduce the mitochondrial population (371).

#### 3.4.8 Mitochondria → HIF-1α

The main factor activating HIF-1α from the mitochondria is mito-ROS. Brandes et al. presented the detailed locations in cytochromes where the electron leakage takes place (128, 269). The system of cytochromes and electron leakage can be modelled by a pipe with holes in which the NADH concentration represents the input pressure, the O_2_ concentration represents the suction force, and the holes in the pipe represent the sites of electron leakage from cytochromes. They showed that the leakage does not depend on the flow rate but on the “pressure” of electrons inside the cytochromes. Both increased input pressure (NADH concentration) and increased output pressure (low O_2_ concentration) increase electron leakage approximately according to the laws of fluid flow through the tube (128, 269). Thus, blocking both complex I and complex II in different ways resulted in a decrease in OXPHOS activity, a decrease in electron leakage from complex III, an increase in oxygen concentration and a decrease in ROS production, thereby affecting the reduction of HIF-1α activity (372, 373). However, Chandel et al. (374) confirmed that hypoxia increased mitochondrial ROS generation at complex III, leading to the accumulation of HIF-1α protein, demonstrating that mitochondrial-derived ROS are both necessary and sufficient to initiate HIF-1α stabilisation during hypoxia.

The other HIF-1α stabilising factors are, as mentioned above, succinate and fumarate. Downregulation of succinate dehydrogenase (SDH) and fumarate hydratase (FH) activities, which are common hallmarks of cancers, results in the accumulation of succinate, inhibition of PHD activity and induction of HIF-1α (375).

To summarise the role of HIF-1α in generating a response to intracellular pathogens, on the one hand, it has a protective effect on the mitochondria, protecting them from the oxidative stress accompanying the inflammatory response; on the other hand, it participates in the response to pathogens by contributing to the enhancement of the positive feedback between inflammation, ROS and Ca_i_^2+^. Excessive excitation of HIF-1α during such a response can lead to excessive mitochondrial deactivation, resulting in a deficiency of the energy to remove pathogens and free radicals and pump Ca_i_^2+^ out of the cytoplasm.

#### 3.4.9 HIF-1α → NO

HIF-1α is one of the activators of iNOS gene transcription leading to increased NO production (376, 377). At physiological concentrations, this is likely to contribute to the enhanced cytoprotective effect of HIF-1α. Another metabolic element linking HIF-1α to NO is fumarate. It has been postulated in the context of hypertension that when fumarate accumulates in the cell, which may occur when HIF-1α is expressed, there is a reduced availability of l-arginine necessary for NOS action, which is formed during the breakdown of argininosuccinate into fumarate and l-arginine, leading to a reduction in NO production (378, 379).

### 3.5 Autophagy

**Figure 6:**
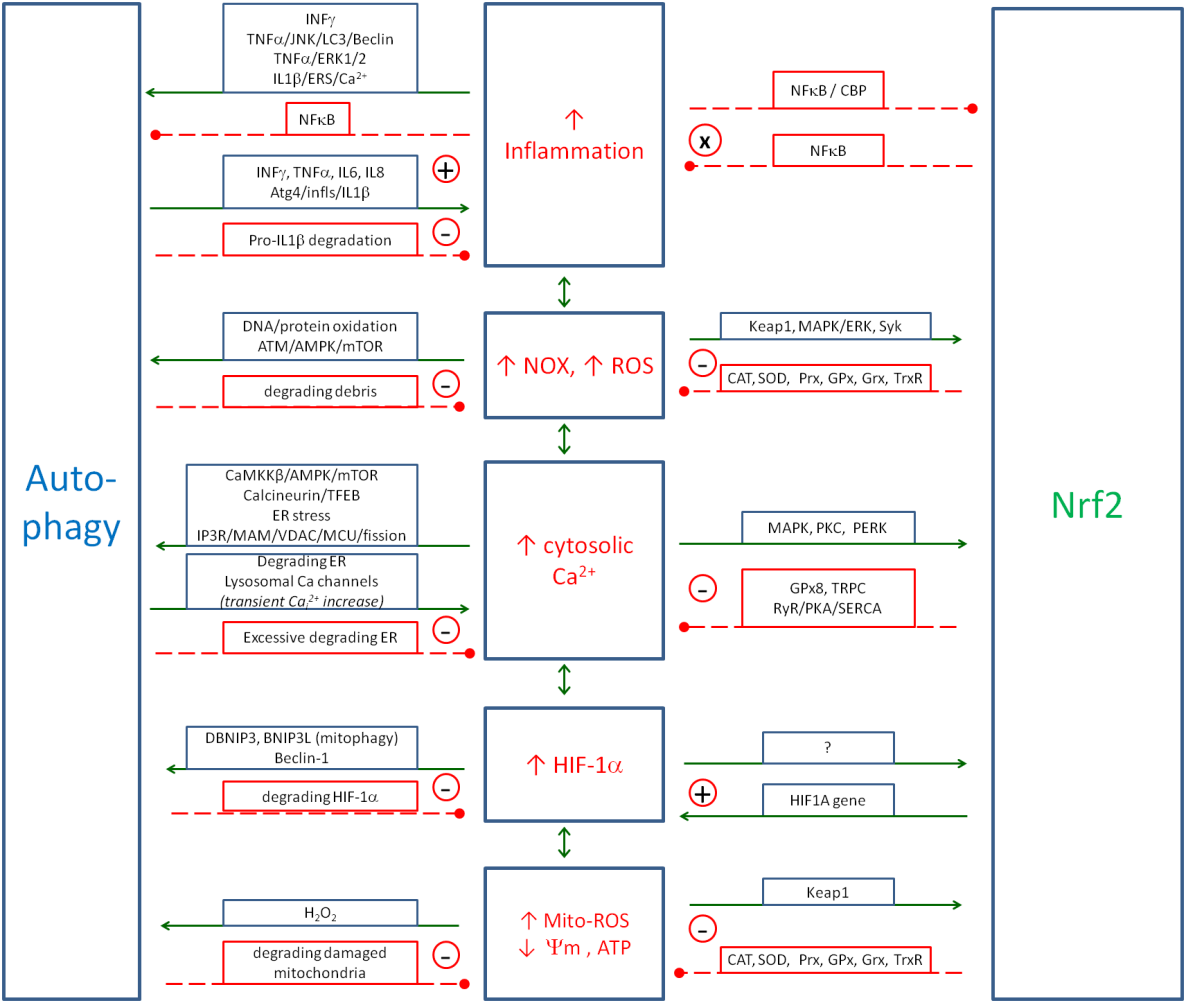
Regulatory role of autophagy and Nrf2 on the Positive Coupling System (PCS; inflammation/ROS/NO/HIF-1α/Ca_i_^2+^). The figure shows the metabolic pathways by which Nrf2 and autophagy control and inhibit the self-excitation of PCS. In two cases, however, the relationship is positive. NF-κB and Nrf2 (marked with **x**) inhibit each other, and such a double inhibitory coupling serves as a switch between the inflammatory and antioxidant states—activation of one inhibits the other and vice versa. In the case of HIF-1α and Nrf2, the coupling is not quite positive. Nrf2 activates HIF-1α to support the mitochondrial protection provided by HIF-1α. In addition, inhibition of one element also inhibits the other and vice versa, which seems to support the termination of the antiviral response. In the case of the relationship between inflammation and autophagy, both positive and negative couplings are observed. The positive ones seem to enhance the antiviral response in the early phase of infection. The couplings with iNOS/NO are shown in **Figure 3**. Solid arrows, activation; red dashed lines with •, inhibition; ⊕, positive coupling; ⊝, negative coupling; ⊗, double-negative coupling. Nrf2, nuclear factor erythroid 2-related factor 2; ROS, reactive oxygen species; NO, nitric oxide; HIF-1α, hypoxia-inducible factor 1-alpha; iNOS, inducible nitric oxide synthase.

An important element of the antiviral defence is the proper functioning of the autophagy system, which is involved in the clearance of pathogen proteins from the cell. The growing interest in autophagy is related to the observation that many intracellular pathogens chronically induce autophagy blockade, thereby preventing their complete clearance from the cell (1–6). Three subcategories of autophagy have been defined—chaperone-mediated autophagy, microautophagy, and macroautophagy—collectively referred to as autophagy (380). The autophagic cascade occurs constitutively at a basal level in various cells and is initiated under stress conditions, such as endoplasmic reticulum stress (ERS), growth factor deprivation, nutrient deprivation, mitochondrial damage and inflammation. Autophagy is also coupled to oxidative stress and inflammation, and the couplings are context-dependent, positive to drive or negative to control the level of antimicrobial metabolic excitation. **Figure 6** shows a summary of the couplings between autophagy and the PCS.

#### 3.5.1 Autophagy ↔ inflammation

Inflammatory cytokines are also involved in the autophagy processes. One of the main functions of autophagy is to eliminate intracellular pathogens, so autophagy must work in concert with the immune system. The interaction between autophagy and inflammation is very complex, and both positive and negative couplings can be observed. Almost all cytokines are coupled to autophagy in different ways. The most important pathways linking inflammation and autophagy are shown in **Figure 6**. The most important cytokines in this process are IFN-γ, TNF-α, IL-1, IL-2, IL-4, IL-6, IL-10 and IL-17 (381–385). In the majority of cases, autophagy activation dominates over inhibition. The main pathways involved in the activation are MAPK—ERK1/2 (386, 387), JNK (388) and p38 (22, 389–392)—which in different ways promote the transcription of key autophagy genes and the production of autophagy proteins including the Atg family, Beclin1, microtubule-associated protein 1 light chain 3 (LC3), DRAM1, TSC1/2 and GBP1/IRGM.

The other signalling pathways involved in autophagy inhibition are JAK1/2-STAT1 (385), PI3K/Akt and NF-κB (390–392). mTOR is the key negative regulator of autophagy, and its inhibition is an important target of several autophagy pathways. The major inhibitors of mTOR are AMPK (106, 107), TSC1/2 (245) and JNK (58, 59). The PI3K/AKT and ERK1/2 signalling pathways activate mTOR (48). The inhibitory effect on autophagy is also observed in the case of the transcription factor NF-κB, and two pathways of this activity are described. It activates mTOR and Bcl-2/BCL-xL proteins, both of which are inhibitors of autophagy (393–396).

#### 3.5.2 p38 ↔ NF-κB coupling

The p38 MAPK and NF-κB are the elements of inflammatory regulation associated with autophagy that interact with each other in a positive loop coupling (397–399). Understanding this coupling appears to be important for understanding the development of the chronic state in some autoimmune diseases (400–404), as well as the severe course of COVID-19.

The p38 MAPK kinase can be activated by stress signals such as cytokines, UV irradiation, heat shock and osmotic shock (22, 389). Once activated, p38 can phosphorylate a number of substrates including transcription factors such as NF-κB (405), which then modulate gene expression in response to stress. NF-κB is typically activated by pro-inflammatory cytokines (such as TNF-α and IL-1), bacterial or viral infections, and other stressors. Its activation leads to the translocation of NF-κB from the cytoplasm to the nucleus, where it influences the expression of genes involved in immune response, cell survival and proliferation. p38 can enhance NF-κB activation by phosphorylating NF-κB itself or its inhibitory protein IκB (inhibitor of kappa B). The phosphorylation of IκB typically leads to its degradation, freeing NF-κB to enter the nucleus and activate transcription. Conversely, components regulated or produced by NF-κB, such as TNF-α, IL-1β, IL-6, IFN-γ, GM-CSF and IL-17, can activate the p38 MAPK pathway (390–392), creating a feedback loop that amplifies the response to stress.

In the context of autophagy, NF-κB generally acts as an inhibitor (393, 394). When activated, NF-κB can suppress the expression of several autophagy-related genes, thereby inhibiting the autophagic process (394–396). It also activates mTORc1, Bcl-2 and Bcl-xL, which are known autophagy inhibitors. The inhibitory effect of NF-κB on autophagy is thought to be a mechanism that favours cell survival and inflammation. However, p38 has been shown to promote autophagy (406, 407). This involves the phosphorylation of several downstream targets that can initiate the autophagic process.

As p38 and NF-κB are positively coupled, both are increased in the inflammatory state, and the overall effect depends on the cellular context. This bidirectional activity of p38/NF-κB coupling on autophagy may be one of the metabolic pathways leading to chronic persistence when autophagy inhibition prevails over stimulation, as autophagy inhibition and NF-κB overactivity are the hallmarks of the chronic infectious or autoimmune state (404, 408, 409). It should be noted that to inhibit autophagy, it is sufficient to inhibit one element of the entire complex pathway of its activation. In contrast, to increase autophagy activity, none of the regulatory elements must be blocked. Only under these conditions can the stimulation of the pathway have a stimulatory effect.

When analysing the impact of autophagy on the inflammatory process, two main effects can be discussed. The first is the reduction of cellular debris that typically activates PCS elements. This cleaning of the cell is important for the return to a healthy state but does not reflect the direct inhibition of the PCS elements. The second effect is the direct activation or inhibition of inflammation by autophagy molecules or by the autophagy process. Autophagy can facilitate the production and release of pro-inflammatory cytokines (410–413). For example, it supports the processing and maturation of IL-1β by delivering cytokine precursors to inflammasomes (414)​​. However, autophagy can reduce inflammation by degrading pro-inflammatory cytokines and the components of inflammasomes that are responsible for their activation. This process helps to control excessive inflammatory responses and maintain cellular homeostasis (415)​​. Multiple connections complicate the analysis; the final effect is context-dependent and requires further research.

#### 3.5.3 Autophagy ↔ oxidative stress

One of the major autophagy regulators is oxidative stress. The relationship between the two is complex. The main link is negative coupling, where ROS mainly activate autophagy but autophagy reduces the tendency to generate ROS. The accumulation of oxidative stress causes oxidation and damage to cellular components, including proteins, DNA and lipids, which turn on the autophagic process (416). Mitochondrial ROS, produced primarily in the mitochondrial electron transport chain, play a critical role in signalling pathways that activate autophagy (417). ROS production increases under stress conditions, such as nutrient deprivation leading to the activation of autophagic processes (418, 419). H_2_O_2_ is relatively stable compared to other ROS and can diffuse between cellular compartments, acting as a signalling molecule to modify proteins involved in autophagosome formation. For example, H_2_O_2_ can oxidise the cysteine residue on Atg4, promoting the lipidation of LC3, a critical step in autophagosome maturation (418). ROS can also activate the ataxia-telangiectasia mutated (ATM) kinase, which then triggers downstream signalling to AMPK (420–422). The activation of AMPK results in the promotion of autophagic processes to maintain cellular homeostasis (423, 424). ROS levels are also associated with reduced energy production, which in turn activates signalling pathways such as the aforementioned AMPK and inhibits the mammalian target of rapamycin (mTOR), a key negative regulator of autophagy (425, 426).

However, autophagy functions to reduce oxidative stress by degrading damaged mitochondria and other cellular debris that would otherwise contribute to increased ROS production (427–429). This protective role is critical in preventing the accumulation of oxidative damage, thereby maintaining cellular integrity and function. Impairment of autophagy increases the oxidative stress (430). Furthermore, antioxidant molecules moderately or completely suppress autophagic execution (431).

In summary, the relationship between ROS and autophagy is not linear but is characterised by a complex feedback mechanism in which ROS induce autophagy, and autophagy can modulate ROS levels. This feedback is essential for adaptation to environmental and metabolic stresses.

#### 3.5.4 Autophagy ↔ HIF-1α

Both autophagy and HIF-1α are essential for cellular survival and homeostasis under hypoxic conditions and are linked by multiple regulatory mechanisms. In general, there is a negative coupling between them. HIF-1α activates autophagy (432, 433), and autophagy reduces the HIF-1α activity by degrading this molecule (434).

HIF-1α can induce the expression of several genes involved in autophagy. For example, HIF-1α has been shown to upregulate BNIP3 and BNIP3-like (BNIP3L), which are involved in mitophagy, the selective autophagy of the mitochondria. By promoting the removal of dysfunctional mitochondria, these proteins help to maintain cellular energy production and reduce ROS levels under hypoxia (435). HIF-1α can also directly enhance autophagy by interacting with the autophagy-related protein Beclin-1 and promoting the formation of autophagosomes. This helps the cell conserve resources and maintain energy production during periods of oxygen deprivation (436).

#### 3.5.5 Autophagy couplings—summary

Looking at **Figure 6**, essentially all of the PCS elements have autophagy-activating activity, and autophagy essentially inhibits all PCS elements. This creates a generalised negative feedback that allows the inflammation and other PCS elements to be silenced, as the amount of pathogen in the cell decreases, and the cell can gradually return to normal function.

There is some complexity in the interactions between autophagy and inflammation, where, especially in the early stages of infection, positive feedback can be observed to enhance inflammation through autophagy. These relationships require more detailed studies, as the overall effect of autophagy on inflammation may be activating or inhibiting at different stages of infection. However, it should be noted that the direct inhibitory effect of autophagy is only observed against inflammation and not against other PCS elements. Therefore, it seems that autophagy cannot be identified as an element that directly controls and inhibits the amplitudes of the PCS spiral.

A separate topic related to the coupling between autophagy and PCS is the issue of chronic infection. Numerous publications have indicated that pathogens with the ability to enter a chronic state within the cell have the ability to block autophagy (1–6, 437–440)​. By removing damaged organelles and intracellular pathogens, autophagy prevents the accumulation of microbial antigens that can trigger inflammatory pathways. This cleaning process is critical for preventing chronic inflammation and has been implicated in diseases such as atherosclerosis and autoimmune disorders (441, 442)​​. When autophagy is blocked, the cell is unable to completely remove the pathogen, leading to continued low levels of activation of the PCS system, but not enough to stimulate autophagy to remove the pathogen completely (443). A dynamic equilibrium is then created between the presence of a small amount of the pathogen in the cell, and non-lethal activation of the PCS system is then produced, which manifests in the patient with symptoms of chronic fatigue associated with mitochondrial uncoupling and possibly mild inflammatory symptoms such as pain, redness, swelling or exudation, depending on the location of the pathogen (444–449). However, more research is needed to determine how much of the chronic inflammation of different organs is related to the presence of chronic pathogens and/or impaired autophagy.

### 3.6 Regulatory role of NRf2

The positive couplings that drive the immune response against the pathogens must be controlled by a number of negative regulators to prevent the response from going beyond the level of self-destruction. One of the key negative regulators that directly control the PCS spiral is the transcription factor Nrf2.

#### 3.6.1 Nrf2 ↔ oxidative stress

Nrf2 is the major nuclear transcription receptor that activates the production of several proteins involved in detoxification and oxidative stress reduction. Oxidative stress induces an antioxidant response as a compensatory mechanism through the activation of the Nrf2 signalling pathway. At low levels of ROS, Nrf2 is associated with the Keap1, which targets Nrf2 for proteasomal degradation. At high levels of ROS, Nrf2 dissociates from Keap1 and translocates to the nucleus, where it binds to the antioxidant response elements (AREs) of target gene promoters (450). The proteins produced include catalase (CAT), SOD, peroxiredoxin (Prx), thioredoxin (Trx), GPx, glutaredoxin (Grx), metallothioneins (MTs), glutathione reductase (GSR), Trx reductase (TrxR) and sulfiredoxin (Srx). Many of the antioxidant enzymes/proteins regulated by Nrf2 are localised to specific compartments within the cell to control redox signalling in the local environment. Nrf2 also regulates the expression of several oxidant signalling proteins, thereby affecting programmed cellular functions. Some regulators, such as p62 and DJ-1, activate Nrf2 and can be triggered by oxidants via Nrf2, forming a positive feedback loop with Nrf2 to increase its activity (451).

#### 3.6.2 Nrf2 ↔ inflammation

Nrf2 is the main factor that reduces the level of ROS in the cell, but it has also been described to reduce the effects of inflammation. The relationship between Nrf2 and inflammation is complex. In particular, there is a double-negative coupling between Nrf2 and NF-κB, which needs to be explained in detail (452). Nrf2 negatively regulates the NF-κB pathway through several mechanisms. First, Nrf2 inhibits oxidative stress-mediated NF-κB activation by reducing the intracellular ROS levels (453). In addition, Nrf2 prevents proteasomal degradation of IκBα and inhibits nuclear translocation of NF-κB (454, 455).

A direct effect of Nrf2 on inflammation has also been observed. According to a study by Jiang et al. (456), Nrf2 mediates anti-inflammatory signalling in macrophages, which plays a critical role in preventing liver ischaemia/reperfusion injury by blocking the transcription of pro-inflammatory cytokines​​. In another study, Nrf2 suppressed the inflammatory response of macrophages by blocking the transcription of pro-inflammatory cytokines, which was independent of ROS levels (457). This study identifies Nrf2 as a negative upstream regulator of cytokine production and provides a molecular basis for an Nrf2-mediated anti-inflammatory approach (458).

The inverse relationship is also negative. NF-κB decreases free CBP, which is a transcriptional co-activator of Nrf2. This inhibitory effect of NF-κB occurs by competing with the CH1-KIX domain of CBP while also promoting the phosphorylation of p65 at Ser276, which in turn prevents CBP from binding to Nrf2 (458, 459). This results in double-negative coupling between Nrf2 and NF-κB, which is actually a type of positive coupling between them. On the one hand, high Nrf2 activity reduces NF-κB activity, and reduced NF-κB activity contributes to increased Nrf2 activity. Conversely, high NF-κB activity reduces Nrf2 activity, and reduced Nrf2 activity further contributes to increased NF-κB activity. This mechanism helps to stabilise the cell in a state of inflammation and fight against some pathology (high NF-κB/low Nrf2) or in a state of health (low NF-κB/high Nrf2). It is important to note that such a regulatory mechanism is an unstable point of self-regulation, and even a small stimulus can tip the balance towards hyperinflammation or recovery. Therefore, targeting the activation of Nrf2 and the inhibition of NF-κB may be a promising therapeutic target against various types of chronic inflammation.

#### 3.6.3 Nrf2 ↔ calcium stress

Calcium plays an important role in several cellular signalling pathways, but its relationship with Nrf2 is indirect rather than direct. Calcium can activate various signalling pathways, such as MAPK pathways, which can modulate Nrf2 activity (460, 461). In particular, certain MAPKs such as ERK1/2 and p38 can phosphorylate Nrf2, increasing its stability and nuclear accumulation. The other pathway is PKC, a family of enzymes that are activated by calcium and DAG. Activated PKC can phosphorylate Nrf2 or its regulatory proteins, thereby affecting Nrf2 activation and the subsequent antioxidant response (462, 463).

The Nrf2 plays a critical role in cellular defence mechanisms against oxidative stress by regulating the expression of several antioxidant and cytoprotective genes. Its influence extends to maintaining intracellular calcium levels and modulating ER stress responses. The main effect of Nrf2 on ER stress is to increase the expression of glutathione peroxidase GPx8, a critical enzyme involved in protein folding and ER homeostasis (464). On the other hand, ER stress activates Nrf2 through a PERK-dependent mechanism (465), as PERK is known to directly phosphorylate Nrf2 and induce its dissociation from Keap1 without the involvement of ROS. Nrf2 is also able to reduce intracellular calcium by suppressing the redox-sensitive TRPC channels, thereby inhibiting calcium influx in renal podocytes (466). Another study showed that Nrf2 plays a protective role in the process of oxidative stress-induced Ca_i_^2+^ increase in skeletal muscle and that Nrf2 inhibition increased RyR and PKA protein expression in C2C12 cells, improved sarcoplasmic reticulum calcium release function, decreased SERCA protein expression and reduced sarcoplasmic reticulum calcium recovery, all of which contributed to the increase in Ca_i_^2+^ (467). In conclusion, Nrf2 and Ca_i_^2+^ work in the classical negative coupling, where Ca_i_^2+^ induces Nrf2 and Nrf2 reduces the calcium stress.

#### 3.6.4 Nrf2 ↔ HIF-1α

The interplay between Nrf2 and HIF-1α is poorly studied. Available publications have suggested a significant synergistic relationship between these factors. The general link between Nrf2 and HIF-1α is through the action of ROS, which induces both these transcription factors (468). Two signalling pathways have been postulated to transduce ROS signals into Nrf2 and HIF-1α activation. Jang et al. showed that the ROS-sensitive spleen tyrosine kinase (Syk) is able to activate both transcription factors in B cells (469). In another study, Wang showed that a similar effect can be observed via the ROS-activated ERK1/2 pathway (470). Lacher et al. (471) showed that the gene for HIF-1α transcription is one of the non-canonical targets of Nrf2 and that Nrf2 activates HIF-1α transcription. This study found that Nrf2 activity is associated with high HIF-1α gene expression in several cellular contexts. In addition, Wang et al. (470) observed that inhibition of Nrf2 reduced HIF-1α activity, which is consistent with Lacher’s findings. They also observed that inhibition of HIF-1α reduced Nrf2 activity. A similar effect of Nrf2 inhibition by HIF-1α inhibitors was observed by Jang (469), but it is not clear whether these effects were direct or indirect, e.g. by reducing Ca_i_^2+^ levels.

This type of coupling qualifies it as a positive one. However, it is necessary to distinguish between positive coupling, in which two elements are mutually stimulated, and coupling, in which inhibition of one element also causes inhibition of the other and vice versa. In this case, there is no mutual excitation leading to the self-destruction of the system. According to previous studies, the excitatory effect is only observed in one direction (Nrf2 → HIF-1α), so the self-activation loop is not closed. Thus, the reciprocal coupling is more related to the mutual silencing of the two factors.

The whole relationship can be interpreted as a synergistic protective effect of both factors under oxidative stress, where Nrf2 activates the production of antioxidant enzymes and HIF-1α reduces the production of free radicals by the mitochondria. From this point of view, HIF-1α appears to be an ambivalent element. On the one hand, it is involved in the enhancement of the PCS spiral, and on the other hand, it helps stabilise it. The sum of its effects depends very much on the metabolic context.

## 4 Summary

A growing number of studies describing the relationships between individual signalling pathways and other pieces of the molecular puzzle are making it possible to construct increasingly complex graphs of interactions to understand the workings of the cell as a whole. The paper presents a fairly universal mechanism of interactions between seven key elements of self-regulation that form the cell’s self-regulatory mechanism in response to various intracellular pathogens and other stressors. A literature review of all the mutual interactions between the seven elements of cellular homeostasis has been analysed, from which a picture emerges of the mutual excitation of the five elements mainly to enhance the response against pathogens and the controlling effect of autophagy, Nrf2 and partially HIF-1α and NO as elements to prevent excessive escalation of this response. To the authors’ knowledge, this is the first such comprehensive analysis of the regulatory interactions. Particular attention should be paid to the Nrf2/HO-1 axis and autophagy. It should be noted that even a slight weakening of the control of these positive feedbacks can result in the new equilibrium state of the system being far beyond the adaptive capacity of the cell. However, at least in the theory, even a small improvement in the control of such mechanisms can significantly shift the equilibrium of regulated elements towards the correct and adequate level for fighting the pathogen. However, it must be remembered that as long as the pathogen is present in the cells, the balance can be shifted towards chronic inflammation, chronic oxidative, nitrosative and calcium stress.

The other general conclusion can be drawn about the strategy for treating chronic inflammation. The multiplicity of reciprocal positive couplings suggests that a one-drug strategy may be doomed to failure. Reducing only one of the five elements involved in the reciprocal positive couplings would not reduce the others, as they remain in the positive loops and continue to drive the system into pathology and chronic inflammation. According to the authors’ opinion, at least three to four upregulated elements should be downregulated simultaneously to achieve the final effect of reducing all the coupled components. The optimal way is to use drugs or herbs that regulate all the elements presented. In this case, an additive or even hyper-additive effect would be expected from the co-application of drugs acting on different dysregulated elements of the overall regulation. Special attention should be paid to herbs, as they usually have a well-defined low toxicity, and there is increasing evidence that many of them influence the elements of metabolism discussed above (472–489).

It is important to note that the relationships presented between the coupled elements are relatively universal and can probably be applied to many diseases in medicine, including acute and chronic infections, and autoimmune and degenerative diseases. However, individual diseases and the tissues involved are likely to differ in the strength of the mutual feedback and the molecular details.

Chronic intracellular infections remain a significant medical problem. Examples of such pathogens include Lyme disease, *Bartonella*, chlamydia, *Mycoplasma*, tuberculosis and viruses such as SARS-CoV-2, Epstein–Barr virus (EBV), herpes group, Coxsackie group, Ebola virus, Zika virus, enteroviruses and measles virus (490–500). SARS-CoV-2 is reported to be chronic, and it is postulated to be one of the causes of the long COVID syndrome (501–510). EBV and cytomegalovirus are also capable of causing chronic inflammation and coagulopathies, including disseminated intravascular coagulation (DIC) (511–513). Current therapies against these pathogens are long and often of limited efficacy. New drugs are constantly being sought to cure cells of chronic pathogens. Understanding the molecular relationships can greatly accelerate the identification of therapeutic strategies. In particular, many studies have indicated that intracellular pathogens block autophagy as a common component of impaired intracellular metabolism in their chronic state (1–6).

Autoimmune and degenerative diseases are different from chronic infectious diseases. In the case of intracellular infections, many pathogen proteins disrupt many physiological regulatory couplings, which seem to require multiple drug therapies in order to regulate the disrupted couplings and clear pathogens from the cell. In the case of autoimmune or degenerative diseases, the number of initial perturbed elements is likely to be limited, and the problem of the optimal therapy is to find the metabolic element that is the first domino to fall, further triggering all other dysregulations.

### 4.1 Limitations of the article

The article outlines the many feedbacks involved in the self-regulation of the inflammatory process, but it has some limitations. The main limitation is that the many molecular interactions described were derived from a large number of experiments based on different cell lines. Further research is needed to determine whether the described interactions are universal or only occur in selected cell types. In particular, cells of the immune system may be subject to significantly different regulatory mechanisms in relation to their function than host cells attacked by pathogens or autoantibodies.

Another limitation is that only a subset of molecular elements was analysed (seven core elements and seven kinase signalling pathways). The analysed transcription factors activate the production of a large number of different proteins, which may also affect the analysed feedback in different ways. Thus, the described feedback system should be treated more as a framework for extending the model, incorporating more elements into it and analysing their effects on elements of the current model. Knowledge within the currently analysed elements is also constantly evolving. In particular, the cytokine system is extremely complex in its operation and knowledge of its effects on other elements of the regulatory system evolves.

Another limitation is that, for some relationships, there are a number of studies indicating opposite effects of one element on another through different pathways, making it difficult or impossible to determine the exact relationship. The opposing pathways of interaction may depend on the concentration/activity of the element, or they may be part of a more complex regulation that allows the activity to be better adapted to the metabolic context. This requires more detailed studies that may significantly modify the current model, in particular metabolic contexts.

### 4.2 Challenges for future research

The present article represents an important turning point in the development of molecular biology because, to the best of the authors’ knowledge, it is the first attempt to build a multidimensional model of cellular self-regulation according to the rules of control theory. In such systems where many elements are mutually coupled, a change in one element causes a change in all the others, and a new equilibrium state is reached. There are important conclusions to be drawn from this article, which are different for molecular biologists and physicians. Molecular biologists focus on the correctness of the relationships between elements, while physicians focus on finding the causes of disease.

From the molecular biologist’s point of view, it is necessary to develop the regulatory model to be analysed. The first conclusion is the need to carefully validate whether the influence of one factor on another is direct, forming an elementary edge in the graph of interrelationships, or indirect, i.e. through other elements in the graph of interrelationships.

The second tip for future experiments is the need to simultaneously examine the influence of a specific factor on many regulatory parameters of the model, preferably all of them. For example, when studying the effect of a particular drug, it is recommended to examine changes in cytokine levels; NOX and iNOS activities; NF-κB, HIF-1α and Nrf2 activities; Ca_i_^2+^ levels, autophagy activation, signalling pathways activity, etc. This will give a more holistic picture of the effect on metabolism.

Next, in the future research perspective, it is necessary to undertake a study to describe each edge of the graph of interrelationships between elements by means of appropriate differential equations. The exemplary ordinary differential equation-based dynamic model describing the response of Nrf2, Keap1, Srxn1 and GSH to oxidative stress was described by Hiemstra et al. (514). This work provides a future perspective for molecular biology. Once the equations for each interaction are mathematically described, it will be possible to create a generalised system of differential equations describing the behaviour of the system, as well as to model the behaviour of the system under the action of deregulatory stimuli (e.g. viral proteins) and/or drugs. This will allow the mathematical optimisation of therapies for various diseases.

In order to build mathematical models, it is necessary to study the variability of concentrations and activities of individual system components in the time domain to observe the magnitude and dynamics of changes in activity under the influence of a specific stimulus. In order to fit equations to time courses, it is necessary to have at least several measurements of parameters at different times after the stimulus. In view of the above, it is necessary in the future to collaborate with computer scientists specialising in the analysis of the self-regulation of multidimensional systems in order to build mathematical models of metabolic self-regulation. This distant goal must be kept in mind when designing experiments so that the data obtained can be of value in building such future mathematical models.

From the perspective of a scientist looking for a cure for a particular disease, building a multidimensional molecular model of the disease will facilitate the search for a cure. The challenge for molecular biology in this case is to build a qualitative and then a mathematical regulatory model of the diseases. The number of diseases in which perturbations in cytokines, calcium stress, mitochondrial stress, reticuloendoplasmic stress, autophagy, HIF-1α or Nrf2 are observed is very large and includes autoimmune diseases (515–517), neurodegenerative diseases (518–520), cardiovascular disease (521–523), type 2 diabetes (524, 525), hypertension (526), obesity (527), metabolic syndrome (528), non-alcoholic steatohepatitis (529–531), chronic obstructive pulmonary disease (532–534), depression (535–537) and schizophrenia (538–540). Particular attention should be paid to sepsis, a metabolic state in which inflammation and oxidative stress are particularly high and directly life-threatening (541). Thus, the above model has a very wide range of potential applications in medicine because, as presented, all these elements are coupled. However, it is important to bear in mind that the current model is likely to be only a part of the larger individual disease models that will be developed in the future.

In a system with many feedback loops, it is often not easy to identify the first domino that triggers a cascade of changes in the cell leading to the development of the disease. The study of parameter variability in the time domain will make it possible to determine the sequence of changes and thus potentially identify the initiating factor. However, the study of the effect of a given drug on all elements of self-regulation will make it possible, at the level of cellular experiments, to better determine whether a given drug has a chance of being effective in treating/curing a specific disease. In the case of diseases with chronic inflammation, it may be necessary to look for combinations of drugs that effectively balance all the dysregulated elements of self-regulation at the same time, as it is generally unlikely that a single drug will balance all the metabolic disorders. This is generally possible if it balances the first element of the metabolic cascade.

A comprehensive understanding of the intricate network of cellular self-regulation not only deepens our knowledge of disease mechanisms but also paves the way for more targeted and effective therapeutic strategies, marking a crucial step towards precision medicine.

## References

[1] Dutta S Ganguly A Ghosh Roy S. An Overview of the Unfolded Protein Response (Upr) and Autophagy Pathways in Human Viral Oncogenesis. Int Rev Cell Mol Biol (2024) 386:81–131. doi: 10.1016/bs.ircmb.2024.01.004 38782502

[2] Liu L-K Jian J-T Jing S-S Gao R-L Chi X-D Tian G. The crustacean DNA virus tegument protein vp26 binds to snap29 to inhibit snare complex assembly and autophagic degradation. J Virol. (2024) 98:e01408–23. doi: 10.1128/jvi.01408-23 PMC10878264 38189252

[3] Wyatt S Glover K Dasanna S Lewison M González-García M Colbert CL. Epstein–barr virus encoded bcl2, bhrf1, downregulates autophagy by noncanonical binding of becn1. Biochemistry. (2023) 62:2934–51. doi: 10.1021/acs.biochem.3c00225 PMC11166532 37776275

[4] Nagdev PK Agnivesh PK Roy A Sau S Kalia NP. Exploring and exploiting the host cell autophagy during mycobacterium tuberculosis infection. Eur J Clin Microbiol Infect Dis. (2023) 42:1297–315. doi: 10.1007/s10096-023-04663-0 37740791

[5] Shi Y Wu Z Zeng P Song J Guo J Yang X. Seneca valley virus 3c protease blocks epha2-mediated mtor activation to facilitate viral replication. Microbial Pathogenesis. (2024) 191:106673. doi: 10.1016/j.micpath.2024.106673 38705218

[6] Chen B Guo G Wang G Zhu Q Wang L Shi W. Atg7/gaplinc/irf3 axis plays a critical role in regulating pathogenesis of influenza a virus. PloS Pathog. (2024) 20:e1011958. doi: 10.1371/journal.ppat.1011958 38227600 PMC10817227

[7] Di Vincenzo F Del Gaudio A Petito V Lopetuso LR Scaldaferri F. Gut microbiota, intestinal permeability, and systemic inflammation: A narrative review. Internal Emergency Med. (2024) 19:275–93. doi: 10.1007/s11739-023-03374-w PMC10954893 37505311

[8] Chen WW Zhang X Huang WJ. Role of neuroinflammation in neurodegenerative diseases (Review). Mol Med Rep. (2016) 13:3391–6. doi: 10.3892/mmr.2016.4948 PMC4805095 26935478

[9] Ferreira IL Resende R Ferreiro E Rego AC Pereira CF. Multiple defects in energy metabolism in alzheimer’s disease. Curr Drug Targets. (2010) 11:1193–206. doi: 10.2174/1389450111007011193 20840064

[10] Burbelo PD Iadarola MJ Keller JM Warner BM. Autoantibodies targeting intracellular and extracellular proteins in autoimmunity. Front Immunol. (2021) 12. doi: 10.3389/fimmu.2021.548469 PMC7982651 33763057

[11] Ferreira CM Vieira AT Vinolo MA Oliveira FA Curi R Martins Fdos S. The central role of the gut microbiota in chronic inflammatory diseases. J Immunol Res. (2014) 2014:689492. doi: 10.1155/2014/689492 25309932 PMC4189530

[12] Corre I Paris F Huot J. The P38 pathway, a major pleiotropic cascade that transduces stress and metastatic signals in endothelial cells. Oncotarget. (2017) 8:55684–714. doi: 10.18632/oncotarget.18264 PMC5589692 28903453

[13] Winston BW Chan ED Johnson GL Riches DW. Activation of P38mapk, mkk3, and mkk4 by tnf-alpha in mouse bone marrow-derived macrophages. J Immunol. (1997) 159:4491–7. doi: 10.4049/jimmunol.159.9.4491 9379049

[14] Chen N-N Wei F Wang L Cui S Wan Y Liu S. Tumor necrosis factor alpha induces neural stem cell apoptosis through activating P38 mapk pathway. Neurochemical Res. (2016) 41:3052–62. doi: 10.1007/s11064-016-2024-8 27528245

[15] Wang XJ Kong KM Qi WL Ye WL Song PS. Interleukin-1 beta induction of neuron apoptosis depends on P38 mitogen-activated protein kinase activity after spinal cord injury. Acta Pharmacologica Sin. (2005) 26:934–42. doi: 10.1111/j.1745-7254.2005.00152.x 16038625

[16] Liu X Ye F Xiong H Hu D Limb G Xie T. Il-1 beta upregulates il-8 production in human muller cells through activation of the P38 mapk and erk1/2 signaling pathways. Inflammation. (2014) 37:1486–95. doi: 10.1007/s10753-014-9874-5 24706000

[17] Chen F Wang Q Li Y Li F Zhang L Gu X. Tgf-β1-induced apoptosis in retinal endothelial cells is implicated in retinal vein occlusion. Exp Eye Res. (2024) 250:110168. doi: 10.1016/j.exer.2024.110168 39577604

[18] Chen H Wang M Zhang Z Lin F Guo B Lu Q. Oxidative stress drives endometrial fibrosis via tgf-β1/mapk signaling pathway in breast cancer. FASEB J. (2024) 38:e70172. doi: 10.1096/fj.202401257RR 39548950

[19] Kang YJ Chen J Otsuka M Mols J Ren S Wang Y. Macrophage deletion of P38α Partially impairs lipopolysaccharide-induced cellular activation1. J Immunol. (2008) 180:5075–82. doi: 10.4049/jimmunol.180.7.5075 18354233

[20] Masson-Gadais B Houle F Laferrière J Huot J. Integrin alphavbeta3, requirement for vegfr2-mediated activation of sapk2/P38 and for hsp90-dependent phosphorylation of focal adhesion kinase in endothelial cells activated by vegf. Cell Stress Chaperones. (2003) 8:37–52. doi: 10.1379/1466-1268(2003)8<37:ivrfva>2.0.co;2 12820653 PMC514852

[21] Jung YD Fan F McConkey DJ Jean ME Liu W Reinmuth N. Role of P38 mapk, ap-1, and nf-κb in interleukin-1β-induced il-8 expression in human vascular smooth muscle cells. Cytokine. (2002) 18:206–13. doi: 10.1006/cyto.2002.1034 12126643

[22] Suzuki M Tetsuka T Yoshida S Watanabe N Kobayashi M Matsui N. The role of P38 mitogen-activated protein kinase in il-6 and il-8 production from the tnf-α- or il-1β-stimulated rheumatoid synovial fibroblasts. FEBS Lett. (2000) 465:23–7. doi: 10.1016/S0014-5793(99)01717-2 10620700

[23] Westra J Doornbos-van-der-Meer B de Boer P van Leeuwen MA van Rijswijk MH Limburg PC. Strong inhibition of tnf-α Production and inhibition of il-8 and cox-2 mrna expression in monocyte-derived macrophages by rwj 67657, a P38 mitogen-activated protein kinase (Mapk) inhibitor. Arthritis Res Ther. (2004) 6:R384. doi: 10.1186/ar1204 15225374 PMC464924

[24] Chen K-H Weng M-S Lin J-K. Tangeretin suppresses il-1β-induced cyclooxygenase (Cox)-2 expression through inhibition of P38 mapk, jnk, and akt activation in human lung carcinoma cells. Biochem Pharmacol. (2007) 73:215–27. doi: 10.1016/j.bcp.2006.09.018 17067555

[25] Beltrán AE Briones AM García-Redondo AB Rodríguez C Miguel M Álvarez Y. P38 mapk contributes to angiotensin ii-induced cox-2 expression in aortic fibroblasts from normotensive and hypertensive rats. J Hypertension. (2009) 27:142–54. doi: 10.1097/HJH.0b013e328317a730 19145780

[26] Whitaker RH Cook JG. Stress relief techniques: P38 mapk determines the balance of cell cycle and apoptosis pathways. Biomolecules. (2021) 11:1444. doi: 10.3390/biom11101444 34680077 PMC8533283

[27] Yue J López JM. Understanding mapk signaling pathways in apoptosis. Int J Mol Sci. (2020) 21:2346. doi: 10.3390/ijms21072346 32231094 PMC7177758

[28] Thornton TM Rincon M. Non-classical P38 map kinase functions: cell cycle checkpoints and survival. Int J Biol Sci. (2009) 5:44–51. doi: 10.7150/ijbs.5.44 19159010 PMC2610339

[29] Leelahavanichkul K Amornphimoltham P Molinolo AA Basile JR Koontongkaew S Gutkind JS. A role for P38 mapk in head and neck cancer cell growth and tumor-induced angiogenesis and lymphangiogenesis. Mol Oncol. (2014) 8:105–18. doi: 10.1016/j.molonc.2013.10.003 PMC3946852 24216180

[30] Sui X Kong N Ye L Han W Zhou J Zhang Q. P38 and jnk mapk pathways control the balance of apoptosis and autophagy in response to chemotherapeutic agents. Cancer Lett. (2014) 344:174–9. doi: 10.1016/j.canlet.2013.11.019 24333738

[31] Lucas RM Luo L Stow JL. Erk1/2 in immune signalling. Biochem Soc Trans. (2022) 50:1341–52. doi: 10.1042/BST20220271 PMC9704528 36281999

[32] Chen J Liao MY Gao X-L Zhong Q Tang T-T Yu X. Il-17a induces pro-inflammatory cytokines production in macrophages via mapkinases, nf-κb and ap-1. Cell Physiol Biochem. (2013) 32:1265–74. doi: 10.1159/000354525 24247374

[33] Wong E Xu F Joffre J Nguyen N Wilhelmsen K Hellman J. Erk1/2 has divergent roles in lps-induced microvascular endothelial cell cytokine production and permeability. Shock. (2021) 55:349–56. doi: 10.1097/SHK.0000000000001639 PMC8139579 32826812

[34] Saryeddine L Zibara K Kassem N Badran B El-Zein N. Egf-induced vegf exerts a pi3k-dependent positive feedback on erk and akt through vegfr2 in hematological *in vitro* models. PloS One. (2016) 11:e0165876. doi: 10.1371/journal.pone.0165876 27806094 PMC5091849

[35] Quément CL Guénon I Gillon J-Y Lagente V Boichot E. mp-12 induces il-8/cxcl8 secretion through egfr and erk1/2 activation in epithelial cells. Am J Physiology-Lung Cell Mol Physiol. (2008) 294:L1076–L84. doi: 10.1152/ajplung.00489.2007 18390828

[36] Zhou J Sun X Zhang J Yang Y Chen D Cao J. Il-34 regulates il-6 and il-8 production in human lung fibroblasts via mapk, pi3k-akt, jak and nf-κb signaling pathways. Int Immunopharmacol. (2018) 61:119–25. doi: 10.1016/j.intimp.2018.05.023 29857241

[37] Shan L Redhu N Saleh A Halayko A Chakir J Gounni A. Thymic stromal lymphopoietin receptor-mediated il-6 and cc/cxc chemokines expression in human airway smooth muscle cells: role of mapks (Erk1/2, P38, and jnk) and stat3 pathways. J Immunol (Baltimore Md: 1950). (2010) 184:7134–43. doi: 10.4049/jimmunol.0902515 20483734

[38] Liu-Bryan R O’Connell M Johnson K Pritzker K Mackman N Terkeltaub R. Extracellular signal–regulated kinase 1/extracellular signal–regulated kinase 2 mitogen-activated protein kinase signaling and activation of activator protein 1 and nuclear factor κb transcription factors play central roles in interleukin-8 expression stimulated by monosodium urate monohydrate and calcium pyrophosphate crystals in monocytic cells. Arthritis Rheumatism. (2000) 43:1145–55. doi: 10.1002/1529-0131(200005)43:5<1145::AID-ANR25>3.0.CO;2-T 10817569

[39] Dai J Peng L Fan K Wang H Wei R Ji G. Osteopontin induces angiogenesis through activation of pi3k/akt and erk1/2 in endothelial cells. Oncogene. (2009) 28:3412–22. doi: 10.1038/onc.2009.189 19597469

[40] Liu L-Z Li C Chen Q Jing Y Carpenter R Jiang Y. Mir-21 induced angiogenesis through akt and erk activation and hif-1α Expression. PloS One. (2011) 6:e19139. doi: 10.1371/journal.pone.0019139 21544242 PMC3081346

[41] Chen J Luo X Liu M Peng L Zhao Z He C. Silencing long non-coding rna neat1 attenuates rheumatoid arthritis via the mapk/erk signalling pathway by downregulating microrna-129 and microrna-204. RNA Biol. (2021) 18:657–68. doi: 10.1080/15476286.2020.1857941 PMC8078539 33258403

[42] Huang Y Xu L Yang Q Xiao X Ye Z Li R. Nlrp12 C.1382dup promotes the development of crohn’s disease through the erk/nlrp3/il-1β Pathway. Gene. (2024) 931:148855. doi: 10.1016/j.gene.2024.148855 39181275

[43] Savoia P Fava P Casoni F Cremona O. Targeting the erk signaling pathway in melanoma. Int J Mol Sci. (2019) 20:1483. doi: 10.3390/ijms20061483 30934534 PMC6472057

[44] Wang Z-Q Wu D-C Huang F-P Yang G-Y. Inhibition of mek/erk 1/2 pathway reduces pro-inflammatory cytokine interleukin-1 expression in focal cerebral ischemia. Brain Res. (2004) 996:55–66. doi: 10.1016/j.brainres.2003.09.074 14670631

[45] Schreiber A Viemann D Schöning J Schloer S Mecate Zambrano A Brunotte L. The mek1/2-inhibitor atr-002 efficiently blocks sars-cov-2 propagation and alleviates pro-inflammatory cytokine/chemokine responses. Cell Mol Life Sci. (2022) 79:65. doi: 10.1007/s00018-021-04085-1 35013790 PMC8747446

[46] Menon MB Dhamija S. Beclin 1 phosphorylation – at the center of autophagy regulation. Front Cell Dev Biol. (2018) 6:137. doi: 10.3389/fcell.2018.00137 30370269 PMC6194997

[47] Ferreira-Marques M Carvalho A Cavadas C Aveleira CA. Pi3k/akt/mtor and erk1/2-mapk signaling pathways are involved in autophagy stimulation induced by caloric restriction or caloric restriction mimetics in cortical neurons. Aging (Albany NY). (2021) 13:7872–82. doi: 10.18632/aging.202805 PMC8034898 33714946

[48] Wu Y Zhou BP. Kinases meet at tsc. Cell Res. (2007) 17:971–3. doi: 10.1038/cr.2007.106 18075516

[49] Ahn EH Park J-B. Molecular mechanisms of alzheimer’s disease induced by amyloid-β and tau phosphorylation along with rhoa activity: perspective of rhoa/rho-associated protein kinase inhibitors for neuronal therapy. Cells. (2025) 14:89. doi: 10.3390/cells14020089 39851517 PMC11764136

[50] Li Z-Y Zhang Y-P Zhang J Zhang S-B Li D Huang Z-Z. The possible involvement of jnk activation in the spinal dorsal horn in bortezomib-induced allodynia: the role of tnf-α and il-1β. J Anesth. (2016) 30:55–63. doi: 10.1007/s00540-015-2077-x 26373954

[51] Son Y Cheong YK Kim NH Chung HT Kang DG Pae HO. Mitogen-activated protein kinases and reactive oxygen species: how can ros activate mapk pathways? J Signal transduction. (2011) 2011:792639. doi: 10.1155/2011/792639 PMC3100083 21637379

[52] Sabapathy K. Role of the Jnk Pathway in Human Diseases. Prog Mol Biol Transl Sci. (2012) 106:145–69. doi: 10.1016/b978-0-12-396456-4.00013-4 22340717

[53] Raivich G Behrens A. Role of the ap-1 transcription factor C-jun in developing, adult and injured brain. Prog Neurobiol. (2006) 78:347–63. doi: 10.1016/j.pneurobio.2006.03.006 16716487

[54] Namba S Nakano R Kitanaka T Kitanaka N Nakayama T Sugiya H. Erk2 and jnk1 contribute to tnf-α-induced il-8 expression in synovial fibroblasts. PloS One. (2017) 12:e0182923. doi: 10.1371/journal.pone.0182923 28806729 PMC5555573

[55] Chen WC Tseng CK Chen YH Lin CK Hsu SH Wang SN. Hcv ns5a up-regulates cox-2 expression via il-8-mediated activation of the erk/jnk mapk pathway. PloS One. (2015) 10:e0133264. doi: 10.1371/journal.pone.0133264 26231035 PMC4521820

[56] Mollereau B Ma D. Rb-mediated apoptosis or proliferation: it’s up to jnk. Cell Cycle. (2016) 15:11–2. doi: 10.1080/15384101.2015.1119492 PMC4825755 26588003

[57] Ventura JJ Cogswell P Flavell RA Baldwin AS Jr. Davis RJ. Jnk potentiates tnf-stimulated necrosis by increasing the production of cytotoxic reactive oxygen species. Genes Dev. (2004) 18:2905–15. doi: 10.1101/gad.1223004 PMC534651 15545623

[58] Tang H-W Liao H-M Peng W-H Lin H-R Chen C-H Chen G-C. Atg9 interacts with dtraf2/traf6 to regulate oxidative stress-induced jnk activation and autophagy induction. Dev Cell. (2013) 27:489–503. doi: 10.1016/j.devcel.2013.10.017 24268699

[59] Zhou Y-Y Li Y Jiang W-Q Zhou L-F. Mapk/jnk signalling: A potential autophagy regulation pathway. Bioscience Rep. (2015) 35:e00199. doi: 10.1042/BSR20140141 PMC4613668 26182361

[60] Denninger K Rasmussen S Larsen JM Ørskov C Seier Poulsen S Sørensen P. Jnk1, but not jnk2, is required in two mechanistically distinct models of inflammatory arthritis. Immunometabolism. (2020) 2(2):e200012. doi: 10.1016/j.ajpath.2011.06.019 PMC3181375 21839715

[61] Yung JHM Giacca A. Role of C-jun N-terminal kinase (Jnk) in obesity and type 2 diabetes. Cells. (2020) 9:706. doi: 10.3390/cells9030706 32183037 PMC7140703

[62] Batlle R Andrés E Gonzalez L Llonch E Igea A Gutierrez-Prat N. Regulation of tumor angiogenesis and mesenchymal–endothelial transition by P38α through tgf-β and jnk signaling. Nat Commun. (2019) 10:3071. doi: 10.1038/s41467-019-10946-y 31296856 PMC6624205

[63] Tam SY Law HK. Jnk in tumor microenvironment: present findings and challenges in clinical translation. Cancers (Basel). (2021) 13:2196. doi: 10.3390/cancers13092196 34063627 PMC8124407

[64] Vergadi E Ieronymaki E Lyroni K Vaporidi K Tsatsanis C. Akt signaling pathway in macrophage activation and M1/M2 polarization. J Immunol. (2017) 198:1006–14. doi: 10.4049/jimmunol.1601515 28115590

[65] Xie S Chen M Yan B He X Chen X Li D. Identification of a role for the pi3k/akt/mtor signaling pathway in innate immune cells. PloS One. (2014) 9:e94496. doi: 10.1371/journal.pone.0094496 24718556 PMC3981814

[66] Hou C-H Tang C-H Hsu C-J Hou S-M Liu J-F. Ccn4 induces il-6 production through αvβ5 receptor, pi3k, akt, and nf-κb singling pathway in human synovial fibroblasts. Arthritis Res Ther. (2013) 15:R19. doi: 10.1186/ar4151 23343403 PMC3672729

[67] Zhao M Zhou A Xu L Zhang X. The role of tlr4-mediated pten/pi3k/akt/nf-κb signaling pathway in neuroinflammation in hippocampal neurons. Neuroscience. (2014) 269:93–101. doi: 10.1016/j.neuroscience.2014.03.039 24680857

[68] Tseng W-P Su C-M Tang C-H. Fak activation is required for tnf-α-induced il-6 production in myoblasts. J Cell Physiol. (2010) 223:389–96. doi: 10.1002/jcp.22047 20082310

[69] Simão F Matté A Pagnussat AS Netto CA Salbego CG. Resveratrol Prevents Ca1 Neurons against Ischemic Injury by Parallel Modulation of Both Gsk-3β and Creb through Pi3-K/Akt Pathways. Eur J Neurosci. (2012) 36:2899–905. doi: 10.1111/j.1460-9568.2012.08229.x 22817531

[70] Wan X Zhou M Huang F Zhao N Chen X Wu Y. Akt1-creb stimulation of pdgfrα Expression is pivotal for pten deficient tumor development. Cell Death Dis. (2021) 12:172. doi: 10.1038/s41419-021-03433-0 33568640 PMC7876135

[71] Callahan V Hawks S Crawford MA Lehman CW Morrison HA Ivester HM. The pro-inflammatory chemokines cxcl9, cxcl10 and cxcl11 are upregulated following sars-cov-2 infection in an akt-dependent manner. Viruses. (2021) 13:1062. doi: 10.3390/v13061062 34205098 PMC8226769

[72] Günzl P Bauer K Hainzl E Matt U Dillinger B Mahr B. Anti-inflammatory properties of the pi3k pathway are mediated by il-10/dusp regulation. J Leukocyte Biol. (2010) 88:1259–69. doi: 10.1189/jlb.0110001 20884649

[73] Zhang X Li N Shao H Meng Y Wang L Wu Q. Methane limit lps-induced nf-κb/mapks signal in macrophages and suppress immune response in mice by enhancing pi3k/akt/gsk-3β-mediated il-10 expression. Sci Rep-Uk. (2016) 6:29359. doi: 10.1038/srep29359 PMC4942692 27405597

[74] Lin L Chen H Zhang Y Lin W Liu Y Li T. Il-10 protects neurites in oxygen-glucose-deprived cortical neurons through the pi3k/akt pathway. PloS One. (2015) 10:e0136959. doi: 10.1371/journal.pone.0136959 26366999 PMC4569574

[75] Dugo L Collin M Thiemermann C. Glycogen synthase kinase 3β as a target for the therapy of shock and inflammation. Shock. (2007) 27:113–23. doi: 10.1097/01.shk.0000238059.23837.68 17224784

[76] Hay N. Interplay between foxo, tor, and akt. Biochim Biophys Acta (BBA) - Mol Cell Res. (2011) 1813:1965–70. doi: 10.1016/j.bbamcr.2011.03.013 PMC3427795 21440577

[77] Banham-Hall E Clatworthy MR Okkenhaug K. The therapeutic potential for pi3k inhibitors in autoimmune rheumatic diseases. Open Rheumatol J. (2012) 6:245–58. doi: 10.2174/1874312901206010245 PMC3460535 23028409

[78] Mercurio L Albanesi C Madonna S. Recent updates on the involvement of pi3k/akt/mtor molecular cascade in the pathogenesis of hyperproliferative skin disorders. Front Med-Lausanne. (2021) 8. doi: 10.3389/fmed.2021.665647 PMC8119789 33996865

[79] DuBois JC Ray AK Gruber RC Zhang Y Aflakpui R Macian-Juan F. Akt3-mediated protection against inflammatory demyelinating disease. Front Immunol. (2019) 10. doi: 10.3389/fimmu.2019.01738 PMC6669559 31404142

[80] Jiang N Dai Q Su X Fu J Feng X Peng J. Role of pi3k/akt pathway in cancer: the framework of Malignant behavior. Mol Biol Rep. (2020) 47:4587–629. doi: 10.1007/s11033-020-05435-1 PMC7295848 32333246

[81] Huang X Liu G Guo J Su Z. The pi3k/akt pathway in obesity and type 2 diabetes. Int J Biol Sci. (2018) 14:1483–96. doi: 10.7150/ijbs.27173 PMC6158718 30263000

[82] Savova MS Mihaylova LV Tews D Wabitsch M Georgiev MI. Targeting pi3k/akt signaling pathway in obesity. Biomedicine Pharmacotherapy. (2023) 159:114244. doi: 10.1016/j.biopha.2023.114244 36638594

[83] Dibble CC Cantley LC. Regulation of mtorc1 by pi3k signaling. Trends Cell Biol. (2015) 25:545–55. doi: 10.1016/j.tcb.2015.06.002 PMC4734635 26159692

[84] Kim DH Bang E Ha S Jung HJ Choi YJ Yu BP. Organ-differential roles of akt/foxos axis as a key metabolic modulator during aging. Aging Dis. (2021) 12:1713–28. doi: 10.14336/ad.2021.0225 PMC8460295 34631216

[85] Cao G Lin M Gu W Su Z Duan Y Song W. The rules and regulatory mechanisms of foxo3 on inflammation, metabolism, cell death and aging in hosts. Life Sci. (2023) 328:121877. doi: 10.1016/j.lfs.2023.121877 37352918

[86] Yan Z Gibson SA Buckley JA Qin H Benveniste EN. Role of the jak/stat signaling pathway in regulation of innate immunity in neuroinflammatory diseases. Clin Immunol. (2018) 189:4–13. doi: 10.1016/j.clim.2016.09.014 27713030 PMC5573639

[87] Hu X li J Fu M Zhao X Wang W. The jak/stat signaling pathway: from bench to clinic. Signal Transduction Targeted Ther. (2021) 6:402. doi: 10.1038/s41392-021-00791-1 PMC8617206 34824210

[88] Gough DJ Messina NL Hii L Gould JA Sabapathy K Robertson AP. Functional crosstalk between type I and ii interferon through the regulated expression of stat1. PloS Biol. (2010) 8:e1000361. doi: 10.1371/journal.pbio.1000361 20436908 PMC2860501

[89] Johnson DE O’Keefe RA Grandis JR. Targeting the il-6/jak/stat3 signalling axis in cancer. Nat Rev Clin Oncol. (2018) 15:234–48. doi: 10.1038/nrclinonc.2018.8 PMC5858971 29405201

[90] Zhang D Sun M Samols D Kushner I. Stat3 participates in transcriptional activation of the C-reactive protein gene by interleukin-6. J Biol Chem. (1996) 271:9503–9. doi: 10.1074/jbc.271.16.9503 8621622

[91] Cho SS Bacon CM Sudarshan C Rees RC Finbloom D Pine R. Activation of stat4 by il-12 and ifn-alpha: evidence for the involvement of ligand-induced tyrosine and serine phosphorylation. J Immunol. (1996) 157:4781–9. doi: 10.4049/jimmunol.157.11.4781 8943379

[92] Jones DM Read KA Oestreich KJ. Dynamic roles for il-2-stat5 signaling in effector and regulatory cd4(+) T cell populations. J Immunol. (2020) 205:1721–30. doi: 10.4049/jimmunol.2000612 PMC7513451 32958706

[93] Perez GM Melo M Keegan AD Zamorano J. Aspirin and salicylates inhibit the il-4- and il-13-induced activation of stat6. J Immunol. (2002) 168:1428–34. doi: 10.4049/jimmunol.168.3.1428 11801685

[94] Calautti E Avalle L Poli V. Psoriasis: A stat3-centric view. Int J Mol Sci. (2018) 19:171. doi: 10.3390/ijms19010171 29316631 PMC5796120

[95] Nguyen PM Putoczki TL Ernst M. Stat3-activating cytokines: A therapeutic opportunity for inflammatory bowel disease? J Interferon Cytokine Res. (2015) 35:340–50. doi: 10.1089/jir.2014.0225 PMC4426323 25760898

[96] Yu H Pardoll D Jove R. Stats in cancer inflammation and immunity: A leading role for stat3. Nat Rev Cancer. (2009) 9:798–809. doi: 10.1038/nrc2734 19851315 PMC4856025

[97] Park SY Lee SW Lee SY Hong KW Bae SS Kim K. Sirt1/adenosine monophosphate-activated protein kinase α Signaling enhances macrophage polarization to an anti-inflammatory phenotype in rheumatoid arthritis. Front Immunol. (2017) 8. doi: 10.3389/fimmu.2017.01135 PMC5605563 28966618

[98] Gong H Chen H Xiao P Huang N Han X Zhang J. Mir-146a impedes the anti-aging effect of ampk via nampt suppression and nad(+)/sirt inactivation. Signal Transduct Target Ther. (2022) 7:66. doi: 10.1038/s41392-022-00886-3 35241643 PMC8894495

[99] Han X Tai H Wang X Wang Z Zhou J Wei X. Ampk activation protects cells from oxidative stress-induced senescence via autophagic flux restoration and intracellular nad(+) elevation. Aging Cell. (2016) 15:416–27. doi: 10.1111/acel.12446 PMC4854918 26890602

[100] Kim J Yang G Kim Y Kim J Ha J. Ampk activators: mechanisms of action and physiological activities. Exp Mol Med. (2016) 48:e224. doi: 10.1038/emm.2016.16 27034026 PMC4855276

[101] Cui Y Chen J Zhang Z Shi H Sun W Yi Q. The role of ampk in macrophage metabolism, function and polarisation. J Trans Med. (2023) 21:892. doi: 10.1186/s12967-023-04772-6 PMC10709986 38066566

[102] Ran D Ma Y Liu W Luo T Zheng J Wang D. Tgf-β-activated kinase 1 (Tak1) mediates cadmium-induced autophagy in osteoblasts via the ampk/mtorc1/ulk1 pathway. Toxicology. (2020) 442:152538. doi: 10.1016/j.tox.2020.152538 32693121

[103] Chen X Li X Zhang W He J Xu B Lei B. Activation of ampk inhibits inflammatory response during hypoxia and reoxygenation through modulating jnk-mediated nf-κb pathway. Metabolism. (2018) 83:256–70. doi: 10.1016/j.metabol.2018.03.004 PMC5960613 29526538

[104] Bullón P Alcocer-Gómez E Carrión AM Marín-Aguilar F Garrido-Maraver J Román-Malo L. Ampk phosphorylation modulates pain by activation of nlrp3 inflammasome. Antioxid Redox Signal. (2016) 24:157–70. doi: 10.1089/ars.2014.6120 PMC4742979 26132721

[105] Rabinovitch RC Samborska B Faubert B Ma EH Gravel S-P Andrzejewski S. Ampk maintains cellular metabolic homeostasis through regulation of mitochondrial reactive oxygen species. Cell Rep. (2017) 21:1–9. doi: 10.1016/j.celrep.2017.09.026 28978464

[106] Kim J Kundu M Viollet B Guan K-L. Ampk and mtor regulate autophagy through direct phosphorylation of ulk1. Nat Cell Biol. (2011) 13:132–41. doi: 10.1038/ncb2152 PMC3987946 21258367

[107] Sanduja S Feng Y Mathis RA Sokol ES Reinhardt F Halaban R. Ampk promotes tolerance to ras pathway inhibition by activating autophagy. Oncogene. (2016) 35:5295–303. doi: 10.1038/onc.2016.70 PMC6086350 27041569

[108] Smith BK Marcinko K Desjardins EM Lally JS Ford RJ Steinberg GR. Treatment of nonalcoholic fatty liver disease: role of ampk. Am J Physiology-Endocrinology Metab. (2016) 311:E730–E40. doi: 10.1152/ajpendo.00225.2016 27577854

[109] Ruderman NB Carling D Prentki M Cacicedo JM. Ampk, insulin resistance, and the metabolic syndrome. J Clin Invest. (2013) 123:2764–72. doi: 10.1172/JCI67227 PMC3696539 23863634

[110] He J Ma J Ren B Liu A. Advances in systemic lupus erythematosus pathogenesis via mtor signaling pathway. Semin Arthritis Rheumatism. (2020) 50:314–20. doi: 10.1016/j.semarthrit.2019.09.022 31796213

[111] Piranavan P Bhamra M Perl A. Metabolic targets for treatment of autoimmune diseases. Immunometabolism. (2020) 2:e200012. doi: 10.20900/immunometab20200012 32341806 PMC7184931

[112] Li W Saud SM Young MR Chen G Hua B. Targeting ampk for cancer prevention and treatment. Oncotarget. (2015) 6:7365–78. doi: 10.18632/oncotarget.3629 PMC4480686 25812084

[113] Hsu CC Peng D Cai Z Lin HK. Ampk signaling and its targeting in cancer progression and treatment. Semin Cancer Biol. (2022) 85:52–68. doi: 10.1016/j.semcancer.2021.04.006 33862221 PMC9768867

[114] Minchev D Kazakova M Sarafian V. Neuroinflammation and autophagy in parkinson’s disease—Novel perspectives. Int J Mol Sci. (2022) 23:14997. doi: 10.3390/ijms232314997 36499325 PMC9735607

[115] Rakshe PS Dutta BJ Chib S Maurya N Singh S. Unveiling the interplay of ampk/sirt1/pgc-1α Axis in brain health: promising targets against aging and ndds. Ageing Res Rev. (2024) 96:102255. doi: 10.1016/j.arr.2024.102255 38490497

[116] Li C Chen T Zhou H Feng Y Hoi MPM Ma D. Bhdpc is a novel neuroprotectant that provides anti-neuroinflammatory and neuroprotective effects by inactivating nf-κb and activating pka/creb. Front Pharmacol. (2018) 9:614. doi: 10.3389/fphar.2018.00614 29988625 PMC6027181

[117] Tavares LP Negreiros-Lima GL Lima KM E Silva PMR Pinho V Teixeira MM. Blame the signaling: role of camp for the resolution of inflammation. Pharmacol Res. (2020) 159:105030. doi: 10.1016/j.phrs.2020.105030 32562817

[118] Cherpokova D Jouvene CC Libreros S DeRoo EP Chu L de la Rosa X. Resolvin D4 attenuates the severity of pathological thrombosis in mice. Blood. (2019) 134:1458–68. doi: 10.1182/blood.2018886317 PMC6839959 31300403

[119] Lee H-J Ko H-J Song D-K Jung Y-J. Lysophosphatidylcholine Promotes Phagosome Maturation and Regulates Inflammatory Mediator Production through the Protein Kinase a–Phosphatidylinositol 3 Kinase–P38 Mitogen-Activated Protein Kinase Signaling Pathway During Mycobacterium Tuberculosis Infection in Mouse Macrophages. Front Immunol. (2018) 9:920. doi: 10.3389/fimmu.2018.00920 29755479 PMC5934435

[120] Wang Z Zhang L Liang Y Zhang C Xu Z Zhang L. Cyclic amp mimics the anti-ageing effects of calorie restriction by up-regulating sirtuin. Sci Rep-Uk. (2015) 5:12012. doi: 10.1038/srep12012 PMC4648391 26153625

[121] Signorile A De Rasmo D. Mitochondrial complex I, a possible sensible site of camp pathway in aging. Antioxidants-Basel. (2023) 12:221. doi: 10.3390/antiox12020221 36829783 PMC9951957

[122] Kadenbach B. Regulation of cytochrome C oxidase contributes to health and optimal life. World J Biol Chem. (2020) 11:52–61. doi: 10.4331/wjbc.v11.i2.52 33024517 PMC7520645

[123] García-Bermúdez J Sánchez-Aragó M Soldevilla B del Arco A Nuevo-Tapioles C Cuezva José M. Pka phosphorylates the atpase inhibitory factor 1 and inactivates its capacity to bind and inhibit the mitochondrial H+-atp synthase. Cell Rep. (2015) 12:2143–55. doi: 10.1016/j.celrep.2015.08.052 26387949

[124] Ould Amer Y Hebert-Chatelain E. Mitochondrial camp-pka signaling: what do we really know? Biochim Biophys Acta (BBA) - Bioenergetics. (2018) 1859:868–77. doi: 10.1016/j.bbabio.2018.04.005 29694829

[125] Stephan JS Yeh YY Ramachandran V Deminoff SJ Herman PK. The tor and pka signaling pathways independently target the atg1/atg13 protein kinase complex to control autophagy. Proc Natl Acad Sci U.S.A. (2009) 106:17049–54. doi: 10.1073/pnas.0903316106 PMC2761351 19805182

[126] Papinski D Kraft C. Regulation of autophagy by signaling through the atg1/ulk1 complex. J Mol Biol. (2016) 428:1725–41. doi: 10.1016/j.jmb.2016.03.030 27059781

[127] Bordt EA Polster BM. Nadph oxidase- and mitochondria-derived reactive oxygen species in proinflammatory microglial activation: A bipartisan affair? Free Radical Bio Med. (2014) 76:34–46. doi: 10.1016/j.freeradbiomed.2014.07.033 25091898 PMC4252610

[128] Brand MD. Mitochondrial generation of superoxide and hydrogen peroxide as the source of mitochondrial redox signaling. Free Radic Biol Med. (2016) 100:14–31. doi: 10.1016/j.freeradbiomed.2016.04.001 27085844

[129] Brandes RP Weissmann N Schroder K. Nox family nadph oxidases: molecular mechanisms of activation. Free Radic Biol Med. (2014) 76:208–26. doi: 10.1016/j.freeradbiomed.2014.07.046 25157786

[130] Brandes RP Weissmann N Schroder K. Nox family nadph oxidases in mechano-transduction: mechanisms and consequences. Antioxid Redox Signal. (2014) 20:887–98. doi: 10.1089/ars.2013.5414 PMC3924808 23682993

[131] Kim Y Park SY Jung H Noh YS Lee JJ Hong JY. Inhibition of nadph oxidase 4 (Nox4) signaling attenuates tuberculous pleural fibrosis. J Clin Med. (2019) 8:116. doi: 10.3390/jcm8010116 30669315 PMC6351931

[132] Li Y Li Y Zheng S. Inhibition of nadph oxidase 5 (Nox5) suppresses high glucose-induced oxidative stress, inflammation and extracellular matrix accumulation in human glomerular mesangial cells. Med Sci Monit. (2020) 26:e919399. doi: 10.12659/MSM.919399 32012145 PMC7020764

[133] Yu T Wan P Zhu XD Ren YP Wang C Yan RW. Inhibition of nadph oxidase activities ameliorates dss-induced colitis. Biochem Pharmacol. (2018) 158:126–33. doi: 10.1016/j.bcp.2018.10.010 30321511

[134] Hou L Sun F Huang R Sun W Zhang D Wang Q. Inhibition of nadph oxidase by apocynin prevents learning and memory deficits in a mouse parkinson’s disease model. Redox Biol. (2019) 22:101134. doi: 10.1016/j.redox.2019.101134 30798073 PMC6389731

[135] Zhang Y Deng B Li Z. Inhibition of nadph oxidase increases defense enzyme activities and improves maize seed germination under pb stress. Ecotoxicol Environ Saf. (2018) 158:187–92. doi: 10.1016/j.ecoenv.2018.04.028 29702459

[136] Kovacevic S Ivanov M Miloradovic Z Brkic P Vajic UJ Zivotic M. Hyperbaric oxygen preconditioning and the role of nadph oxidase inhibition in postischemic acute kidney injury induced in spontaneously hypertensive rats. PloS One. (2020) 15:e0226974. doi: 10.1371/journal.pone.0226974 31914135 PMC6948727

[137] Liu H Wang L Pan Y Wang X Ding Y Zhou C. Celastrol alleviates aortic valve calcification via inhibition of nadph oxidase 2 in valvular interstitial cells. JACC Basic Transl Sci. (2020) 5:35–49. doi: 10.1016/j.jacbts.2019.10.004 32043019 PMC7000868

[138] Manea SA Vlad ML Fenyo IM Lazar AG Raicu M Muresian H. Pharmacological inhibition of histone deacetylase reduces nadph oxidase expression, oxidative stress and the progression of atherosclerotic lesions in hypercholesterolemic apolipoprotein E-deficient mice; potential implications for human atherosclerosis. Redox Biol. (2020) 28:101338. doi: 10.1016/j.redox.2019.101338 31634818 PMC6807290

[139] Shin SK Kim KO Kim SH Kwon OS Choi CS Jeong SH. Exogenous 8-hydroxydeoxyguanosine ameliorates liver fibrosis through the inhibition of rac1-nadph oxidase signaling. J Gastroenterol Hepatol. (2020) 35:1078–87. doi: 10.1111/jgh.14979 31907970

[140] Tannich F Tlili A Pintard C Chniguir A Eto B Dang PM. Activation of the phagocyte nadph oxidase/nox2 and myeloperoxidase in the mouse brain during pilocarpine-induced temporal lobe epilepsy and inhibition by ketamine. Inflammopharmacology. (2020) 28:487–97. doi: 10.1007/s10787-019-00655-9 31667656

[141] Tian R Peng R Yang Z Peng YY Lu N. Supplementation of dietary nitrate attenuated oxidative stress and endothelial dysfunction in diabetic vasculature through inhibition of nadph oxidase. Nitric Oxide. (2020) 96:54–63. doi: 10.1016/j.niox.2020.01.007 31972252

[142] Urner S Ho F Jha JC Ziegler D Jandeleit-Dahm K. Nadph oxidase inhibition: preclinical and clinical studies in diabetic complications. Antioxid Redox Signal. (2020) 33:415–34. doi: 10.1089/ars.2020.8047 32008354

[143] Yuan M Meng W Liao W Lian S. Andrographolide antagonizes tnf-alpha-induced il-8 via inhibition of nadph oxidase/ros/nf-kappab and src/mapks/ap-1 axis in human colorectal cancer hct116 cells. J Agric Food Chem. (2018) 66:5139–48. doi: 10.1021/acs.jafc.8b00810 29672044

[144] Wei X Li H Zhang Y Li C Li K Ai K. Ca(2+)-calcineurin axis-controlled nfat nuclear translocation is crucial for optimal T cell immunity in an early vertebrate. J Immunol. (2020) 204:569–85. doi: 10.4049/jimmunol.1901065 31871019

[145] Li J Huang B Shi X Castranova V Vallyathan V Huang C. Involvement of hydrogen peroxide in asbestos-induced nfat activation. Mol Cell Biochem. (2002) 234-235:161–8. doi: 10.1023/A:1015962916195 12162429

[146] Lavrovsky Y Chatterjee B Clark RA Roy AK. Role of redox-regulated transcription factors in inflammation, aging and age-related diseases. Exp Gerontology. (2000) 35:521–32. doi: 10.1016/S0531-5565(00)00118-2 10978675

[147] Unger Benjamin L Ganesan S Comstock Adam T Faris Andrea N Hershenson Marc B Sajjan Uma S. Nod-like receptor X-1 is required for rhinovirus-induced barrier dysfunction in airway epithelial cells. J Virol. (2014) 88:3705–18. doi: 10.1128/jvi.03039-13 PMC3993547 24429360

[148] Tattoli I Carneiro LA Jéhanno M Magalhaes JG Shu Y Philpott DJ. Nlrx1 is a mitochondrial nod-like receptor that amplifies nf-κb and jnk pathways by inducing reactive oxygen species production. EMBO Rep. (2008) 9:293–300. doi: 10.1038/sj.embor.7401161 18219313 PMC2267388

[149] Ohtsu A Shibutani Y Seno K Iwata H Kuwayama T Shirasuna K. Advanced glycation end products and lipopolysaccharides stimulate interleukin-6 secretion via the rage/tlr4-nf-kappab-ros pathways and resveratrol attenuates these inflammatory responses in mouse macrophages. Exp Ther Med. (2017) 14:4363–70. doi: 10.3892/etm.2017.5045 PMC5647727 29067115

[150] Hsu CY Vo TTT Lee CW Chen YL Lin WN Cheng HC. Carbon monoxide releasing molecule-2 attenuates angiotensin ii-induced il-6/jak2/stat3-associated inflammation by inhibiting nadph oxidase- and mitochondria-derived ros in human aortic smooth muscle cells. Biochem Pharmacol. (2022) 198:114978. doi: 10.1016/j.bcp.2022.114978 35218740

[151] Paniagua L Diaz-Cueto L Huerta-Reyes M Arechavaleta-Velasco F. Cadmium exposure induces interleukin-6 production via ros-dependent activation of the erk1/2 but independent of jnk signaling pathway in human placental jeg-3 trophoblast cells. Reprod Toxicol. (2019) 89:28–34. doi: 10.1016/j.reprotox.2019.06.008 31252067

[152] Ha H Lee HB. Reactive oxygen species amplify glucose signalling in renal cells cultured under high glucose and in diabetic kidney. Nephrol (Carlton). (2005) 10 Suppl:S7–10. doi: 10.1111/j.1440-1797.2005.00448.x 16174288

[153] Elbim C Guichard C Dang PMC Fay M Pedruzzi E Demur H. Interleukin-18 primes the oxidative burst of neutrophils in response to formyl-peptides: role of cytochrome B558 translocation and N-formyl peptide receptor endocytosis. Clin Diagn Lab Immunol. (2005) 12:436–46. doi: 10.1128/CDLI.12.3.436-446.2005 PMC1065204 15753257

[154] Vermot A Petit-Härtlein I Smith SME Fieschi F. Nadph oxidases (Nox): an overview from discovery, molecular mechanisms to physiology and pathology. Antioxidants-Basel. (2021) 10:890. doi: 10.3390/antiox10060890 34205998 PMC8228183

[155] Ladik M Valenta H Erard M Vandenabeele P Riquet FB. From tnf-induced signaling to nadph oxidase enzyme activity: methods to investigate protein complexes involved in regulated cell death modalities. Front Cell Death. (2023) 2. doi: 10.3389/fceld.2023.1127330

[156] Tesoriere L Attanzio A Allegra M Gentile C Livrea MA. Indicaxanthin inhibits nadph oxidase (Nox)-1 activation and nf-κb-dependent release of inflammatory mediators and prevents the increase of epithelial permeability in il-1β-exposed caco-2 cells. Br J Nutr. (2013) 111:415–23. doi: 10.1017/S0007114513002663 23931157

[157] Li J Lan T Zhang C Zeng C Hou J Yang Z. Reciprocal activation between il-6/stat3 and nox4/akt signalings promotes proliferation and survival of non-small cell lung cancer cells. Oncotarget. (2015) 6:1031–48. doi: 10.18632/oncotarget.2671 PMC4359215 25504436

[158] Dwivedi G Gran MA Bagchi P Kemp ML. Dynamic redox regulation of il-4 signaling. PloS Comput Biol. (2015) 11:e1004582. doi: 10.1371/journal.pcbi.1004582 26562652 PMC4642971

[159] Saha B Jyothi Prasanna S Chandrasekar B Nandi D. Gene modulation and immunoregulatory roles of interferonγ. Cytokine. (2010) 50:1–14. doi: 10.1016/j.cyto.2009.11.021 20036577

[160] Ha H Debnath B Neamati N. Role of the cxcl8-cxcr1/2 axis in cancer and inflammatory diseases. Theranostics. (2017) 7:1543–88. doi: 10.7150/thno.15625 PMC5436513 28529637

[161] Jendrysik MA Vasilevsky S Yi L Wood A Zhu N Zhao Y. Nadph oxidase-2 derived ros dictates murine dc cytokine-mediated cell fate decisions during cd4 T helper-cell commitment. PloS One. (2011) 6:e28198. doi: 10.1371/journal.pone.0028198 22145029 PMC3228756

[162] Kaur N Naga OS Norell H Al-Khami AA Scheffel MJ Chakraborty NG. T cells expanded in presence of il-15 exhibit increased antioxidant capacity and innate effector molecules. Cytokine. (2011) 55:307–17. doi: 10.1016/j.cyto.2011.04.014 PMC3595556 21602054

[163] Clemente TM Augusto L Angara RK Gilk SD. Coxiella burnetii actively blocks il-17-induced oxidative stress in macrophages. bioRxiv. (2023). doi: 10.1101/2023.03.15.532774

[164] Meka RR Venkatesha SH Dudics S Acharya B Moudgil KD. Il-27-induced modulation of autoimmunity and its therapeutic potential. Autoimmun Rev. (2015) 14:1131–41. doi: 10.1016/j.autrev.2015.08.001 PMC4628569 26253381

[165] Rocic P Lucchesi PA. Nad(P)H oxidases and tgf-β–induced cardiac fibroblast differentiation. Circ Res. (2005) 97:850–2. doi: 10.1161/01.RES.0000190403.87462.bf 16254216

[166] Yang D Elner SG Bian ZM Till GO Petty HR Elner VM. Pro-inflammatory cytokines increase reactive oxygen species through mitochondria and nadph oxidase in cultured rpe cells. Exp Eye Res. (2007) 85:462–72. doi: 10.1016/j.exer.2007.06.013 PMC2094037 17765224

[167] Didion SP. Cellular and oxidative mechanisms associated with interleukin-6 signaling in the vasculature. Int J Mol Sci. (2017) 18:2563. doi: 10.3390/ijms18122563 29186034 PMC5751166

[168] Ali MI Chen X Didion SP. Heterozygous enos deficiency is associated with oxidative stress and endothelial dysfunction in diet-induced obesity. Physiol Rep. (2015) 3:e12630. doi: 10.14814/phy2.12630 26660551 PMC4760452

[169] Kuwano Y Kawahara T Yamamoto H Teshima-Kondo S Tominaga K Masuda K. Interferon-Γ Activates transcription of nadph oxidase 1 gene and upregulates production of superoxide anion by human large intestinal epithelial cells. Am J Physiology-Cell Physiol. (2006) 290:C433–C43. doi: 10.1152/ajpcell.00135.2005 16162660

[170] Hordijk PL. Regulation of nadph oxidases: the role of rac proteins. Circ Res. (2006) 98:453–62. doi: 10.1161/01.RES.0000204727.46710.5e 16514078

[171] Sundaresan M Yu ZX Ferrans VJ Sulciner DJ Gutkind JS Irani K. Regulation of reactive-oxygen-species generation in fibroblasts by rac1. Biochem J. (1996) 318 :379–82. doi: 10.1042/bj3180379 PMC1217632 8809022

[172] Bekhite MM Müller V Tröger SH Müller JP Figulla H-R Sauer H. Involvement of phosphoinositide 3-kinase class ia (Pi3k 110α) and nadph oxidase 1 (Nox1) in regulation of vascular differentiation induced by vascular endothelial growth factor (Vegf) in mouse embryonic stem cells. Cell Tissue Res. (2016) 364:159–74. doi: 10.1007/s00441-015-2303-8 26553657

[173] Mittal M Siddiqui MR Tran K Reddy SP Malik AB. Reactive oxygen species in inflammation and tissue injury. Antioxidants Redox Signaling. (2013) 20:1126–67. doi: 10.1089/ars.2012.5149 PMC3929010 23991888

[174] Bäumer AT ten Freyhaus H Sauer H Wartenberg M Kappert K Schnabel P. Phosphatidylinositol 3-kinase-dependent membrane recruitment of rac-1 and P47phox is critical for α-platelet-derived growth factor receptor-induced production of reactive oxygen species*. J Biol Chem. (2008) 283:7864–76. doi: 10.1074/jbc.M704997200 18070887

[175] Binker MG Binker-Cosen AA Gaisano HY de Cosen RH Cosen-Binker LI. Tgf-β1 increases invasiveness of sw1990 cells through rac1/ros/nf-κb/il-6/mmp-2. Biochem Biophys Res Commun. (2011) 405:140–5. doi: 10.1016/j.bbrc.2011.01.023 21219858

[176] Boussetta T Raad H Bedouhene S Arabi Derkawi R Gougerot-Pocidalo M-A Hayem G. The peptidyl-prolyl isomerase pin1 controls gm-csf-induced priming of nadph oxidase in human neutrophils and priming at inflammatory sites. Int Immunopharmacol. (2024) 137:112425. doi: 10.1016/j.intimp.2024.112425 38851160

[177] Sharma P Chakraborty R Wang L Min B Tremblay ML Kawahara T. Redox regulation of interleukin-4 signaling. Immunity. (2008) 29:551–64. doi: 10.1016/j.immuni.2008.07.019 PMC2631209 18957266

[178] Yamamori T Inanami O Nagahata H Cui Y-D Kuwabara M. Roles of P38 mapk, pkc and pi3-K in the signaling pathways of nadph oxidase activation and phagocytosis in bovine polymorphonuclear leukocytes. FEBS Lett. (2000) 467:253–8. doi: 10.1016/S0014-5793(00)01167-4 10675549

[179] Suzuki K Hino M Kutsuna H Hato F Sakamoto C Takahashi T. Selective activation of P38 mitogen-activated protein kinase cascade in human neutrophils stimulated by il-1β1. J Immunol. (2001) 167:5940–7. doi: 10.4049/jimmunol.167.10.5940 11698472

[180] Ridley SH Sarsfield SJ Lee JC Bigg HF Cawston TE Taylor DJ. Actions of il-1 are selectively controlled by P38 mitogen-activated protein kinase: regulation of prostaglandin H synthase-2, metalloproteinases, and il-6 at different levels. J Immunol. (1997) 158:3165–73. doi: 10.4049/jimmunol.158.7.3165 9120270

[181] Zauberman A Zipori D Krupsky M Ben-Levy R. Stress activated protein kinase P38 is involved in il-6 induced transcriptional activation of stat3. Oncogene. (1999) 18:3886–93. doi: 10.1038/sj.onc.1202738 10445852

[182] Meng F Yamagiwa Y Taffetani S Han J Patel T. Il-6 activates serum and glucocorticoid kinase via P38α Mitogen-activated protein kinase pathway. Am J Physiology-Cell Physiol. (2005) 289:C971–C81. doi: 10.1152/ajpcell.00081.2005 PMC1513290 15917303

[183] Chin BY Mohsenin A Li SX Choi AMK Choi ME. Stimulation of pro-α1(I) collagen by tgf-β1 in mesangial cells: role of the P38 mapk pathway. Am J Physiology-Renal Physiol. (2001) 280:F495–504. doi: 10.1152/ajprenal.2001.280.3.F495 11181412

[184] Lee C-W Lin C-C Lee IT Lee H-C Yang C-M. Activation and induction of cytosolic phospholipase A2 by tnf-α Mediated through nox2, mapks, nf-κb, and P300 in human tracheal smooth muscle cells. J Cell Physiol. (2011) 226:2103–14. doi: 10.1002/jcp.22537 21520062

[185] Ryan S McNicholas WT Taylor CT. A critical role for P38 map kinase in nf-κb signaling during intermittent hypoxia/reoxygenation. Biochem Biophys Res Commun. (2007) 355:728–33. doi: 10.1016/j.bbrc.2007.02.015 17316568

[186] Yu C-G Yezierski RP. Activation of the erk1/2 signaling cascade by excitotoxic spinal cord injury. Mol Brain Res. (2005) 138:244–55. doi: 10.1016/j.molbrainres.2005.04.013 15922485

[187] Hasan RN Schafer AI. Hemin upregulates egr-1 expression in vascular smooth muscle cells via reactive oxygen species erk-1/2–elk-1 and nf-κb. Circ Res. (2008) 102:42–50. doi: 10.1161/CIRCRESAHA.107.155143 17967787

[188] Frias MA James RW Gerber-Wicht C Lang U. Native and reconstituted hdl activate stat3 in ventricular cardiomyocytes via erk1/2: role of sphingosine-1-phosphate. Cardiovasc Res. (2009) 82:313–23. doi: 10.1093/cvr/cvp024 19151362

[189] Lamberti MJ Pansa MF Vera RE Fernández-Zapico ME Rumie Vittar NB Rivarola VA. Transcriptional activation of hif-1 by a ros-erk axis underlies the resistance to photodynamic therapy. PloS One. (2017) 12:e0177801. doi: 10.1371/journal.pone.0177801 28545088 PMC5435305

[190] Qu X Zhang Z Hu W Lou M Zhai B Mei S. Attenuation of the na/K−Atpase/src/ros amplification signaling pathway by astaxanthin ameliorates myocardial cell oxidative stress injury. Mol Med Rep. (2020) 22:5125–34. doi: 10.3892/mmr.2020.11613 PMC7646965 33173978

[191] Balmanno K Cook SJ. Tumour cell survival signalling by the erk1/2 pathway. Cell Death Differentiation. (2009) 16:368–77. doi: 10.1038/cdd.2008.148 18846109

[192] Pacher P Beckman JS Liaudet L. Nitric oxide and peroxynitrite in health and disease. Physiol Rev. (2007) 87:315–424. doi: 10.1152/physrev.00029.2006 17237348 PMC2248324

[193] Yang J Wu L-J Tashino S-I Onodera S Ikejima T. Reactive oxygen species and nitric oxide regulate mitochondria-dependent apoptosis and autophagy in evodiamine-treated human cervix carcinoma hela cells. Free Radical Res. (2008) 42:492–504. doi: 10.1080/10715760802112791 18484413

[194] Toda N Ayajiki K Okamura T. Cerebral blood flow regulation by nitric oxide: recent advances. Pharmacol Rev. (2009) 61:62–97. doi: 10.1124/pr.108.000547 19293146

[195] Lancaster JR. A tutorial on the diffusibility and reactivity of free nitric oxide. Nitric Oxide. (1997) 1:18–30. doi: 10.1006/niox.1996.0112 9701041

[196] Helms C Kim-Shapiro DB. Hemoglobin-mediated nitric oxide signaling. Free Radic Biol Med. (2013) 61:464–72. doi: 10.1016/j.freeradbiomed.2013.04.028 PMC3849136 23624304

[197] Gebicka L Didik J. Catalytic scavenging of peroxynitrite by catalase. J Inorganic Biochem. (2009) 103:1375–9. doi: 10.1016/j.jinorgbio.2009.07.011 19709751

[198] Bryk R Griffin P Nathan C. Peroxynitrite reductase activity of bacterial peroxiredoxins. Nature. (2000) 407:211–5. doi: 10.1038/35025109 11001062

[199] Cheung P-Y Wang W Schulz R. Glutathione protects against myocardial ischemia–reperfusion injury by detoxifying peroxynitrite. J Mol Cell Cardiol. (2000) 32:1669–78. doi: 10.1006/jmcc.2000.1203 10966829

[200] Aktan F. Inos-mediated nitric oxide production and its regulation. Life Sci. (2004) 75:639–53. doi: 10.1016/j.lfs.2003.10.042 15172174

[201] Mendes AF Carvalho AP Caramona MM Lopes MC. Role of nitric oxide in the activation of nf-κb, ap-1 and nos ii expression in articular chondrocytes. Inflammation Res. (2002) 51:369–75. doi: 10.1007/PL00000317 12146729

[202] Obasanjo-Blackshire K Mesquita R Jabr RI Molkentin JD Hart SL Marber MS. Calcineurin regulates nfat-dependent inos expression and protection of cardiomyocytes: co-operation with src tyrosine kinase. Cardiovasc Res. (2006) 71:672–83. doi: 10.1016/j.cardiores.2006.05.026 16828070

[203] Ganster RW Taylor BS Shao L Geller DA. Complex regulation of human inducible nitric oxide synthase gene transcription by stat 1 and nf-κb. Proc Natl Acad Sci. (2001) 98:8638–43. doi: 10.1073/pnas.151239498 PMC37488 11438703

[204] Lu D-Y Liou H-C Tang C-H Fu W-M. Hypoxia-induced inos expression in microglia is regulated by the pi3-kinase/akt/mtor signaling pathway and activation of hypoxia inducible factor-1α. Biochem Pharmacol. (2006) 72:992–1000. doi: 10.1016/j.bcp.2006.06.038 16919605

[205] Kamijo R Harada H Matsuyama T Bosland M Gerecitano J Shapiro D. Requirement for transcription factor irf-1 in no synthase induction in macrophages. Science. (1994) 263:1612–5. doi: 10.1126/science.7510419 7510419

[206] Hecker M Preiß C Schini-Kerth VB. Induction by staurosporine of nitric oxide synthase expression in vascular smooth muscle cells: role of nf-κb, creb and C/ebpβ. Brit J Pharmacol. (1997) 120:1067–74. doi: 10.1038/sj.bjp.0701026 PMC1564581 9134219

[207] Stoicovy RA Cora N Perez A Nagliya D Del Calvo G Lopez TB. Cyclic adenosine monophosphate critically modulates cardiac glp-1 receptor’s anti-inflammatory effects. Inflammation Res. (2024) 73:2043–56. doi: 10.1007/s00011-024-01950-0 39305297

[208] Li D-Y Gao S-J Sun J Zhang L-Q Wu J-Y Song F-H. Targeting the nitric oxide/cgmp signaling pathway to treat chronic pain. Neural Regeneration Res. (2023) 18:996–1003. doi: 10.4103/1673-5374.355748 PMC9827765 36254980

[209] Corti A Franzini M Scataglini I Pompella A. Mechanisms and targets of the modulatory action of S-nitrosoglutathione (Gsno) on inflammatory cytokines expression. Archives of Biochemistry and Biophysics. (2014) 562:80–91. doi: 10.1016/j.abb.2014.08.002 25135357

[210] Surks HK. Cgmp-dependent protein kinase I and smooth muscle relaxation. Circ Res. (2007) 101:1078–80. doi: 10.1161/CIRCRESAHA.107.165779 18040024

[211] Degjoni A Campolo F Stefanini L Venneri MA. The no/cgmp/pkg pathway in platelets: the therapeutic potential of pde5 inhibitors in platelet disorders. J Thromb Haemostasis. (2022) 20:2465–74. doi: 10.1111/jth.15844 PMC9805178 35950928

[212] Rossaneis AC Longhi-Balbinot DT Bertozzi MM Fattori V Segato-Vendrameto CZ Badaro-Garcia S. Ru(Bpy)2(No)So3](Pf6), a nitric oxide donating ruthenium complex, reduces gout arthritis in mice. Front Pharmacol. (2019) 10:229. doi: 10.3389/fphar.2019.00229 30914954 PMC6423075

[213] Manchope MF Calixto-Campos C Coelho-Silva L Zarpelon AC Pinho-Ribeiro FA Georgetti SR. Naringenin inhibits superoxide anion-induced inflammatory pain: role of oxidative stress, cytokines, nrf-2 and the no–cgmp–pkg–katpchannel signaling pathway. PloS One. (2016) 11:e0153015. doi: 10.1371/journal.pone.0153015 27045367 PMC4821586

[214] Nguyen TH Axell A Turek I Wright B Meehan-Andrews T Irving HR. Modulation of inflammatory cytokine production in human monocytes by cgmp and irak3. Int J Mol Sci. (2022) 23:2552. doi: 10.3390/ijms23052552 35269704 PMC8909980

[215] Caviedes A Maturana B Corvalán K Engler A Gordillo F Varas-Godoy M. Enos-dependent S-nitrosylation of the nf-κb subunit P65 has neuroprotective effects. Cell Death Dis. (2021) 12:4. doi: 10.1038/s41419-020-03338-4 33414434 PMC7790835

[216] Reynaert NL Ckless K Korn SH Vos N Guala AS Wouters EFM. Nitric oxide represses inhibitory κb kinase through S-nitrosylation. Proc Natl Acad Sci. (2004) 101:8945–50. doi: 10.1073/pnas.0400588101 PMC428452 15184672

[217] Yee YH Chong SJF Kong LR Goh BC Pervaiz S. Sustained ikkβ Phosphorylation and nf-κb activation by superoxide-induced peroxynitrite-mediated nitrotyrosine modification of B56γ3 and pp2a inactivation. Redox Biol. (2021) 41:101834. doi: 10.1016/j.redox.2020.101834 33838472 PMC8056462

[218] Fürnrohr BG Sheriff A Munoz L von Briesen H Urbonaviciute V Neubert K. Signals, receptors, and cytokines involved in the immunomodulatory and anti-inflammatory properties of apoptotic cells. Signal Transduction. (2005) 5:356–65. doi: 10.1002/sita.200500071

[219] Choi K-S Song E-K Yim C-Y. Cytokines secreted by il-2-activated lymphocytes induce endogenous nitric oxide synthesis and apoptosis in macrophages. J Leukocyte Biol. (2008) 83:1440–50. doi: 10.1189/jlb.1007701 18339892

[220] Hermann M. Cyclooxygenase-2 and nitric oxide. J Cardiovasc Pharmacol. (2006) 47:S21–5. doi: 10.1097/00005344-200605001-00005 16785825

[221] Hortelano S López-Fontal R Través PG Villa N Grashoff C Boscá L. Ilk mediates lps-induced vascular adhesion receptor expression and subsequent leucocyte trans-endothelial migration†. Cardiovasc Res. (2010) 86:283–92. doi: 10.1093/cvr/cvq050 20164118

[222] Schieke SM Briviba K Klotz L-O Sies H. Activation pattern of mitogen-activated protein kinases elicited by peroxynitrite: attenuation by selenite supplementation. FEBS Lett. (1999) 448:301–3. doi: 10.1016/S0014-5793(99)00372-5 10218497

[223] Mander P Brown GC. Activation of microglial nadph oxidase is synergistic with glial inos expression in inducing neuronal death: A dual-key mechanism of inflammatory neurodegeneration. J Neuroinflamm. (2005) 2:20. doi: 10.1186/1742-2094-2-20 PMC1232863 16156895

[224] Sarti P Forte E Mastronicola D Giuffrè A Arese M. Cytochrome C oxidase and nitric oxide in action: molecular mechanisms and pathophysiological implications. Biochim Biophys Acta (BBA) - Bioenergetics. (2012) 1817:610–9. doi: 10.1016/j.bbabio.2011.09.002 21939634

[225] Arora D Jain P Singh N Kaur H Bhatla SC. Mechanisms of nitric oxide crosstalk with reactive oxygen species scavenging enzymes during abiotic stress tolerance in plants. Free Radic Res. (2016) 50:291–303. doi: 10.3109/10715762.2015.1118473 26554526

[226] Nakato R Ohkubo Y Konishi A Shibata M Kaneko Y Iwawaki T. Regulation of the unfolded protein response via S-nitrosylation of sensors of endoplasmic reticulum stress. Sci Rep-Uk. (2015) 5:14812. doi: 10.1038/srep14812 PMC4597200 26446798

[227] Cho IJ Lee AK Lee SJ Lee MG Kim SG. Repression by oxidative stress of inos and cytokine gene induction in macrophages results from ap-1 and nf-κb inhibition mediated by B cell translocation gene-1 activation. Free Radical Bio Med. (2005) 39:1523–36. doi: 10.1016/j.freeradbiomed.2005.07.017 16274887

[228] Lee HJ Oh YK Rhee M Lim J-Y Hwang J-Y Park YS. The role of stat1/irf-1 on synergistic ros production and loss of mitochondrial transmembrane potential during hepatic cell death induced by lps/D-galn. J Mol Biol. (2007) 369:967–84. doi: 10.1016/j.jmb.2007.03.072 17475277

[229] Matalon S Hardiman KM Jain L Eaton DC Kotlikoff M Eu JP. Regulation of ion channel structure and function by reactive oxygen-nitrogen species. Am J Physiology-Lung Cell Mol Physiol. (2003) 285:L1184–L9. doi: 10.1152/ajplung.00281.2003 14604848

[230] Cohen RA Weisbrod RM Gericke M Yaghoubi M Bierl C Bolotina VM. Mechanism of nitric oxide–induced vasodilatation. Circ Res. (1999) 84:210–9. doi: 10.1161/01.RES.84.2.210 9933253

[231] Lipton SA Choi YB Pan ZH Lei SZ Chen HS Sucher NJ. A redox-based mechanism for the neuroprotective and neurodestructive effects of nitric oxide and related nitroso-compounds. Nature. (1993) 364:626–32. doi: 10.1038/364626a0 8394509

[232] Shen K-Z Johnson SW. Ca&Lt;Sup<2+&Lt;/Sup< Influx through Nmda-Gated Channels Activates Atp-Sensitive K&Lt;Sup<+&Lt;/Sup< Currents through a Nitric Oxide–Cgmp Pathway in Subthalamic Neurons. J Neurosci. (2010) 30:1882. doi: 10.1523/JNEUROSCI.3200-09.2010 20130197 PMC2824890

[233] Yoshida T Inoue R Morii T Takahashi N Yamamoto S Hara Y. Nitric oxide activates trp channels by cysteine S-nitrosylation. Nat Chem Biol. (2006) 2:596–607. doi: 10.1038/nchembio821 16998480

[234] Bae H Kim T Lim I. Effects of nitric oxide on apoptosis and voltage-gated calcium channels in human cardiac myofibroblasts. Clin Exp Pharmacol P. (2020) 47:16–26. doi: 10.1111/1440-1681.13178 31519057

[235] Nakamura T Lipton SA. Preventing ca2+-mediated nitrosative stress in neurodegenerative diseases: possible pharmacological strategies. Cell Calcium. (2010) 47:190–7. doi: 10.1016/j.ceca.2009.12.009 PMC2875138 20060165

[236] Weilue H Maria Paula K Eyerusalem G Sijia L Marcelo. Nitric oxide and oxidative stress-mediated cardiovascular functionality: from molecular mechanism to cardiovascular disease. In: Vascular biology. IntechOpen, Rijeka (2019).

[237] Zhang P Huang C Liu H Zhang M Liu L Zhai Y. The mechanism of the nfat transcription factor family involved in oxidative stress response. J Cardiol. (2024) 83:30–6. doi: 10.1016/j.jjcc.2023.04.017 37149283

[238] Altamirano F López JR Henríquez C Molinski T Allen PD Jaimovich E. Increased resting intracellular calcium modulates nf-&X3ba;B-dependent inducible nitric-oxide synthase gene expression in dystrophic <Em>Mdx</em> skeletal myotubes *<Sup> </sup>. J Biol Chem. (2012) 287:20876–87. doi: 10.1074/jbc.M112.344929 PMC3375511 22549782

[239] Di Pietro N Di Tomo P Di Silvestre S Giardinelli A Pipino C Morabito C. Increased inos activity in vascular smooth muscle cells from diabetic rats: potential role of ca2+/calmodulin-dependent protein kinase ii delta 2 (Camkiiδ2). Atherosclerosis. (2013) 226:88–94. doi: 10.1016/j.atherosclerosis.2012.10.062 23177014

[240] Sbardella D Tundo GR Mecchia A Palumbo C Atzori MG Levati L. A novel and atypical nf-kb pro-inflammatory program regulated by a camkii-proteasome axis is involved in the early activation of muller glia by high glucose. Cell Bioscience. (2022) 12:108. doi: 10.1186/s13578-022-00839-x 35842713 PMC9287993

[241] He B F Weber G. Phosphorylation of nf-κb proteins by cyclic gmp-dependent kinase. Eur J Biochem. (2003) 270:2174–85. doi: 10.1046/j.1432-1033.2003.03574.x 12752437

[242] Altura BM Shah NC Shah GJ Zhang A Li W Zheng T. Short-term mg deficiency upregulates protein kinase C isoforms in cardiovascular tissues and cells; relation to nf-kb, cytokines, ceramide salvage sphingolipid pathway and pkc-zeta: hypothesis and review. Int J Clin Exp Med. (2014) 7:1–21. 24482684 PMC3902236

[243] Yang JY Park MY Park SY Yoo HI Kim MS Kim JH. Nitric oxide-induced autophagy in mc3t3-E1 cells is associated with cytoprotection via ampk activation. kjpp. (2015) 19:507–14. doi: 10.4196/kjpp.2015.19.6.507 PMC4637353 26557017

[244] Li Y Zhang Y Wang L Wang P Xue Y Li X. Autophagy impairment mediated by S-nitrosation of atg4b leads to neurotoxicity in response to hyperglycemia. Autophagy. (2017) 13:1145–60. doi: 10.1080/15548627.2017.1320467 PMC5529069 28633005

[245] Sarkar S Korolchuk Viktor I Renna M Imarisio S Fleming A Williams A. Complex inhibitory effects of nitric oxide on autophagy. Mol Cell. (2011) 43:19–32. doi: 10.1016/j.molcel.2011.04.029 21726807 PMC3149661

[246] Liu D Ceddia RP Collins S. Cardiac natriuretic peptides promote adipose ‘Browning’ through mtor complex-1. Mol Metab. (2018) 9:192–8. doi: 10.1016/j.molmet.2017.12.017 PMC5870104 29396369

[247] Shi F Collins S. Regulation of mtor signaling: emerging role of cyclic nucleotide-dependent protein kinases and implications for cardiometabolic disease. Int J Mol Sci. (2023) 24:11497. doi: 10.3390/ijms241411497 37511253 PMC10380887

[248] Um H-C Jang J-H Kim D-H Lee C Surh Y-J. Nitric oxide activates nrf2 through S-nitrosylation of keap1 in pc12 cells. Nitric Oxide. (2011) 25:161–8. doi: 10.1016/j.niox.2011.06.001 21703357

[249] Sawa T Zaki MH Okamoto T Akuta T Tokutomi Y Kim-Mitsuyama S. Protein S-guanylation by the biological signal 8-nitroguanosine 3′,5′-cyclic monophosphate. Nat Chem Biol. (2007) 3:727–35. doi: 10.1038/nchembio.2007.33 17906641

[250] Ihara H Sawa T Nakabeppu Y Akaike T. Nucleotides function as endogenous chemical sensors for oxidative stress signaling. J Clin Biochem Nutr. (2010) 48:33–9. doi: 10.3164/jcbn.11-003FR PMC3022061 21297909

[251] Liang M Knox FG. Nitric oxide activates pkcα and inhibits na+-K+-atpase in opossum kidney cells. Am J Physiology-Renal Physiol. (1999) 277:F859–F65. doi: 10.1152/ajprenal.1999.277.6.F859 10600932

[252] Balafanova Z Bolli R Zhang J Zheng Y Pass JM Bhatnagar A. Nitric oxide (No) induces nitration of protein kinase Cϵ (Pkcϵ), facilitating pkcϵ Translocation via enhanced pkcϵ-rack2 interactions: A novel mechanism of no-triggered activation of pkcϵ*. J Biol Chem. (2002) 277:15021–7. doi: 10.1074/jbc.M112451200 11839754

[253] Huang HC Nguyen T Pickett C. Regulation of the antioxidant response element by protein kinase C-mediated phosphorylation of nf-E2-related factor 2. P Natl Acad Sci USA. (2000) 97 23:12475–80. doi: 10.1073/PNAS.220418997 PMC18788 11035812

[254] Zeng H Wang L Zhang J Pan T Yu Y Lu J. Activated pkb/gsk-3β Synergizes with pkc-Δ Signaling in attenuating myocardial ischemia/reperfusion injury via potentiation of nrf2 activity: therapeutic efficacy of dihydrotanshinone-I. Acta Pharm Sin B. (2021) 11:71–88. doi: 10.1016/j.apsb.2020.09.006 33532181 PMC7838031

[255] Mattart L Calay D Simon D Roebroeck L Caesens-Koenig L Van Steenbrugge M. The peroxynitrite donor 3-morpholinosydnonimine activates nrf2 and the upr leading to a cytoprotective response in endothelial cells. Cell Signalling. (2012) 24:199–213. doi: 10.1016/j.cellsig.2011.09.002 21945407

[256] Kim SG Kim SO. Pkc downstream of pl3-kinase regulates peroxynitrite formation for nrf2-mediated gsta2 induction. Arch Pharm Res. (2004) 27:757–62. doi: 10.1007/bf02980145 15357004

[257] Kang KW Choi SH Kim SG. Peroxynitrite activates nf-E2-related factor 2/antioxidant response element through the pathway of phosphatidylinositol 3-kinase: the role of nitric oxide synthase in rat glutathione S-transferase A2 induction. Nitric Oxide. (2002) 7:244–53. doi: 10.1016/s1089-8603(02)00117-9 12446173

[258] Li M-H Cha Y Surh Y. Peroxynitrite induces ho-1 expression via pi3k/akt-dependent activation of nf-E2-related factor 2 in pc12 cells. Free Radical Biol Med. (2006) 41 7:1079–91. doi: 10.1016/J.FREERADBIOMED.2006.06.010 16962933

[259] Kinobe R Ji Y Nakatsu K. Peroxynitrite-mediated inactivation of heme oxygenases. BMC Pharmacol. (2004) 4:26. doi: 10.1186/1471-2210-4-26 15498099 PMC529254

[260] Sivrikaya A Kolayli S Kucuk M Aliyazicioglu R. *In vitro* effects of peroxynitrite treatment on fish liver catalase activity. J Enzyme Inhibition Medicinal Chem. (2009) 24:432–6. doi: 10.1080/14756360802188313 18825555

[261] MacMillan-Crow LA Thompson JA. Tyrosine modifications and inactivation of active site manganese superoxide dismutase mutant (Y34f) by peroxynitrite. Arch Biochem Biophysics. (1999) 366:82–8. doi: 10.1006/abbi.1999.1202 10334867

[262] Romero-Puertas MC Laxa M Mattè A Zaninotto F Finkemeier I Jones AME. S-nitrosylation of peroxiredoxin ii E promotes peroxynitrite-mediated tyrosine nitration. Plant Cell. (2007) 19:4120–30. doi: 10.1105/tpc.107.055061 PMC2217656 18165327

[263] Benhar M. Roles of mammalian glutathione peroxidase and thioredoxin reductase enzymes in the cellular response to nitrosative stress. Free Radical Bio Med. (2018) 127:160–4. doi: 10.1016/j.freeradbiomed.2018.01.028 29378334

[264] Cleeter MW Cooper JM Darley-Usmar VM Moncada S Schapira AH. Reversible inhibition of cytochrome C oxidase, the terminal enzyme of the mitochondrial respiratory chain, by nitric oxide. Implications Neurodegenerative Diseases. FEBS Lett. (1994) 345:50–4. doi: 10.1016/0014-5793(94)00424-2 8194600

[265] Hausladen A Fridovich I. Superoxide and peroxynitrite inactivate aconitases, but nitric oxide does not. J Biol Chem. (1994) 269:29405–8. doi: 10.1016/S0021-9258(18)43893-8 7961919

[266] Radi R Rodriguez M Castro L Telleri R. Inhibition of mitochondrial electron transport by peroxynitrite. Arch Biochem Biophys. (1994) 308:89–95. doi: 10.1006/abbi.1994.1013 8311480

[267] Verma M Lizama BN Chu CT. Excitotoxicity, calcium and mitochondria: A triad in synaptic neurodegeneration. Trans Neurodegeneration. (2022) 11:3. doi: 10.1186/s40035-021-00278-7 PMC8788129 35078537

[268] Xiao K Jiang L Antonietti M. Ion transport in nanofluidic devices for energy harvesting. Joule. (2019) 3:2364–80. doi: 10.1016/j.joule.2019.09.005

[269] Michalak K Sobolewska-Wlodarczyk A Wlodarczyk M Sobolewska J Wozniak P Sobolewski B. Treatment of the fluoroquinolone-associated disability: the pathobiochemical implications. Oxid Med Cell Longev. (2017) 2017:8023935. doi: 10.1155/2017/8023935 29147464 PMC5632915

[270] Delierneux C Kouba S Shanmughapriya S Potier-Cartereau M Trebak M Hempel N. Mitochondrial calcium regulation of redox signaling in cancer. Cells. (2020) 9:432. doi: 10.3390/cells9020432 32059571 PMC7072435

[271] Shoshan-Barmatz V De Pinto V Zweckstetter M Raviv Z Keinan N Arbel N. Vdac, a multi-functional mitochondrial protein regulating cell life and death. Mol Aspects Med. (2010) 31:227–85. doi: 10.1016/j.mam.2010.03.002 20346371

[272] Hajnóczky G Thomas AP. Minimal requirements for calcium oscillations driven by the ip^3^ receptor. EMBO J. (1997) 16:3533–43-43. doi: 10.1093/emboj/16.12.3533 9218795 PMC1169978

[273] Li J Wang L Chen Y Yang Y Liu J Liu K. Visible light excited ratiometric-gecis for long-term in-cellulo monitoring of calcium signals. Cell Calcium. (2020) 87:102165. doi: 10.1016/j.ceca.2020.102165 32004817

[274] Groenendyk J Wang W-A Robinson A Michalak M. Calreticulin and the heart. Cells. (2022) 11:1722. doi: 10.3390/cells11111722 35681417 PMC9179554

[275] Liu X Green RM. Endoplasmic reticulum stress and liver diseases. Liver Res. (2019) 3:55–64. doi: 10.1016/j.livres.2019.01.002 32670671 PMC7363397

[276] Cascella R Cecchi C. Calcium dyshomeostasis in alzheimer’s disease pathogenesis. Int J Mol Sci. (2021) 22:4914. doi: 10.3390/ijms22094914 34066371 PMC8124842

[277] Lemmer IL Willemsen N Hilal N Bartelt A. A guide to understanding endoplasmic reticulum stress in metabolic disorders. Mol Metab. (2021) 47:101169. doi: 10.1016/j.molmet.2021.101169 33484951 PMC7887651

[278] Melo EP Konno T Farace I Awadelkareem MA Skov LR Teodoro F. Stress-induced protein disaggregation in the endoplasmic reticulum catalysed by bip. Nat Commun. (2022) 13:2501. doi: 10.1038/s41467-022-30238-2 35523806 PMC9076838

[279] Shacham T Sharma N Lederkremer GZ. Protein misfolding and er stress in huntington’s disease. Front Mol Biosci. (2019) 6:20. doi: 10.3389/fmolb.2019.00020 31001537 PMC6456712

[280] Bhattarai KR Riaz TA Kim H-R Chae H-J. The aftermath of the interplay between the endoplasmic reticulum stress response and redox signaling. Exp Mol Med. (2021) 53:151–67. doi: 10.1038/s12276-021-00560-8 PMC8080639 33558590

[281] Uegaki K Tokunaga Y Inoue M Takashima S Inaba K Takeuchi K. The oxidative folding of nascent polypeptides provides electrons for reductive reactions in the er. Cell Rep. (2023) 42:112742. doi: 10.1016/j.celrep.2023.112742 37421625

[282] Fu J Gao J Liang Z Yang D. Pdi-regulated disulfide bond formation in protein folding and biomolecular assembly. Molecules. (2021) 26:171. doi: 10.3390/molecules26010171 PMC7794689 33396541

[283] Konno T Melo EP Chambers JE Avezov E. Intracellular sources of ros/H2o2 in health and neurodegeneration: spotlight on endoplasmic reticulum. Cells. (2021) 10:233. doi: 10.3390/cells10020233 33504070 PMC7912550

[284] Pei J Pan X Wei G Hua Y. Research progress of glutathione peroxidase family (Gpx) in redoxidation. Front Pharmacol. (2023) 14:1147414. doi: 10.3389/fphar.2023.1147414 36937839 PMC10017475

[285] Kanemura S Sofia EF Hirai N Okumura M Kadokura H Inaba K. Characterization of the endoplasmic reticulum-resident peroxidases gpx7 and gpx8 shows the higher oxidative activity of gpx7 and its linkage to oxidative protein folding. J Biol Chem. (2020) 295:12772–85. doi: 10.1074/jbc.RA120.013607 PMC7476714 32719007

[286] Lennicke C Cochemé HM. Redox metabolism: ros as specific molecular regulators of cell signaling and function. Mol Cell. (2021) 81:3691–707. doi: 10.1016/j.molcel.2021.08.018 34547234

[287] Park J Lee S Lee S Kang SW. 2-cys peroxiredoxins: emerging hubs determining redox dependency of mammalian signaling networks. Int J Cell Biol. (2014) 2014:715867. doi: 10.1155/2014/715867 24672551 PMC3932224

[288] Bedard K Krause K-H. The nox family of ros-generating nadph oxidases: physiology and pathophysiology. Physiol Rev. (2007) 87:245–313. doi: 10.1152/physrev.00044.2005 17237347

[289] Wang G Anrather J Huang J Speth RC Pickel VM Iadecola C. Nadph oxidase contributes to angiotensin ii signaling in the nucleus tractus solitarius. J Neurosci. (2004) 24:5516–24. doi: 10.1523/jneurosci.1176-04.2004 PMC6729325 15201324

[290] Zimmerman MC Sharma RV Davisson RL. Superoxide mediates angiotensin ii-induced influx of extracellular calcium in neural cells. Hypertension. (2005) 45:717–23. doi: 10.1161/01.HYP.0000153463.22621.5e 15699459

[291] Wang X Takeda S Mochizuki S Jindal R Dhalla NS. Mechanisms of hydrogen peroxide-induced increase in intracellular calcium in cardiomyocytes. J Cardiovasc Pharmacol Ther. (1999) 4:41–8. doi: 10.1177/107424849900400107 10684523

[292] Granados MP Salido GM González A Pariente JA. Dose-dependent effect of hydrogen peroxide on calcium mobilization in mouse pancreatic acinar cells. Biochem Cell Biol. (2006) 84:39–48. doi: 10.1139/o05-150 16462888

[293] Liu G Pessah IN. Molecular interaction between ryanodine receptor and glycoprotein triadin involves redox cycling of functionally important hyperreactive sulfhydryls. J Biol Chem. (1994) 269:33028–34. doi: 10.1016/S0021-9258(20)30093-4 7806531

[294] Hu Q Yu ZX Ferrans VJ Takeda K Irani K Ziegelstein RC. Critical role of nadph oxidase-derived reactive oxygen species in generating ca2+ Oscillations in human aortic endothelial cells stimulated by histamine. J Biol Chem. (2002) 277:32546–51. doi: 10.1074/jbc.M201550200 12093794

[295] Hu Q Zheng G Zweier JL Deshpande S Irani K Ziegelstein RC. Nadph oxidase activation increases the sensitivity of intracellular ca2+ Stores to inositol 1,4,5-trisphosphate in human endothelial cells. J Biol Chem. (2000) 275:15749–57. doi: 10.1074/jbc.M000381200 10747906

[296] Adachi T Weisbrod RM Pimentel DR Ying J Sharov VS Schöneich C. S-glutathiolation by peroxynitrite activates serca during arterial relaxation by nitric oxide. Nat Med. (2004) 10:1200–7. doi: 10.1038/nm1119 15489859

[297] Redondo PC Salido GM Rosado JA Pariente JA. Effect of hydrogen peroxide on ca2+ Mobilisation in human platelets through sulphydryl oxidation dependent and independent mechanisms. Biochem Pharmacol. (2004) 67:491–502. doi: 10.1016/j.bcp.2003.09.031 15037201

[298] Joseph LC Kokkinaki D Valenti MC Kim GJ Barca E Tomar D. Inhibition of nadph oxidase 2 (Nox2) prevents sepsis-induced cardiomyopathy by improving calcium handling and mitochondrial function. JCI Insight. (2017) 2:e94248. doi: 10.1172/jci.insight.94248 28878116 PMC5621873

[299] Eid BG Abu-Sharib AT El-Bassossy HM Balamash K Smirnov SV. Enhanced calcium entry via activation of nox/pkc underlies increased vasoconstriction induced by methylglyoxal. Biochem Biophys Res Commun. (2018) 506:1013–8. doi: 10.1016/j.bbrc.2018.10.171 30404736

[300] Sumimoto H Minakami R Miyano K. Soluble regulatory proteins for activation of nox family nadph oxidases. Methods Mol Biol. (2019) 1982:121–37. doi: 10.1007/978-1-4939-9424-3_8 31172470

[301] Lassegue B San Martin A Griendling KK. Biochemistry, physiology, and pathophysiology of nadph oxidases in the cardiovascular system. Circ Res. (2012) 110:1364–90. doi: 10.1161/CIRCRESAHA.111.243972 PMC3365576 22581922

[302] Montezano AC Burger D Paravicini TM Chignalia AZ Yusuf H Almasri M. Nicotinamide adenine dinucleotide phosphate reduced oxidase 5 (Nox5) regulation by angiotensin ii and endothelin-1 is mediated via calcium/calmodulin-dependent, rac-1-independent pathways in human endothelial cells. Circ Res. (2010) 106:1363–73. doi: 10.1161/CIRCRESAHA.109.216036 PMC3119893 20339118

[303] Guzik TJ Chen W Gongora MC Guzik B Lob HE Mangalat D. Calcium-dependent nox5 nicotinamide adenine dinucleotide phosphate oxidase contributes to vascular oxidative stress in human coronary artery disease. J Am Coll Cardiol. (2008) 52:1803–9. doi: 10.1016/j.jacc.2008.07.063 PMC2593790 19022160

[304] Schenten V Melchior C Steinckwich N Tschirhart EJ Bréchard S. Sphingosine kinases regulate nox2 activity via P38 mapk-dependent translocation of S100a8/A9. J Leukocyte Biol. (2011) 89:587–96. doi: 10.1189/jlb.0510304 21233411

[305] Xu Y Wang Y Ning K Bao Y. Unraveling the mechanisms of S100a8/A9 in myocardial injury and dysfunction. Curr Issues Mol Biol. (2024) 46:9707–20. doi: 10.3390/cimb46090577 PMC11429546 39329929

[306] Haick JM Brueggemann LI Cribbs LL Denning MF Schwartz J Byron KL. Pkc-dependent regulation of kv7.5 channels by the bronchoconstrictor histamine in human airway smooth muscle cells. Am J Physiol Lung Cell Mol Physiol. (2017) 312:L822–L34. doi: 10.1152/ajplung.00567.2016 28283479

[307] Ruiz A Matute C Alberdi E. Endoplasmic reticulum ca(2+) release through ryanodine and ip(3) receptors contributes to neuronal excitotoxicity. Cell Calcium. (2009) 46:273–81. doi: 10.1016/j.ceca.2009.08.005 19747726

[308] Bacsa B Tiapko O Stockner T Groschner K. Mechanisms and significance of ca(2+) entry through trpc channels. Curr Opin Physiol. (2020) 17:25–33. doi: 10.1016/j.cophys.2020.06.005 33210055 PMC7116371

[309] Jia L Delmotte P Aravamudan B Pabelick CM Prakash YS Sieck GC. Effects of the inflammatory cytokines tnf-alpha and il-13 on stromal interaction molecule-1 aggregation in human airway smooth muscle intracellular ca(2+) regulation. Am J Respir Cell Mol Biol. (2013) 49:601–8. doi: 10.1165/rcmb.2013-0040OC PMC3824046 23713409

[310] Werner LE Wagner U. Calcium-sensing receptor-mediated nlrp3 inflammasome activation in rheumatoid arthritis and autoinflammation. Front Physiol. (2023) 13:1078569. doi: 10.3389/fphys.2022.1078569 36685206 PMC9854345

[311] Pahl HL Baeuerle PA. Activation of nf-kappa B by er stress requires both ca2+ and reactive oxygen intermediates as messengers. FEBS Lett. (1996) 392:129–36. doi: 10.1016/0014-5793(96)00800-9 8772190

[312] Cui T Wang X Hu J Lin T Hu Z Guo H. Molybdenum and cadmium co-exposure induces camkkbeta/ampk/mtor pathway mediated-autophagy by subcellular calcium redistribution in duck renal tubular epithelial cells. J Inorg Biochem. (2022) 236:111974. doi: 10.1016/j.jinorgbio.2022.111974 36027844

[313] Gu Y Qi B Zhou Y Jiang X Zhang X Li X. Porcine circovirus type 2 activates cammkbeta to initiate autophagy in pk-15 cells by increasing cytosolic calcium. Viruses. (2016) 8:135. doi: 10.3390/v8050135 27213427 PMC4885090

[314] Liu X Wang N Zhu Y Yang Y Chen X Chen Q. Extracellular calcium influx promotes antibacterial autophagy in escherichia coli infected murine macrophages via camkkbeta dependent activation of erk1/2, ampk and foxo1. Biochem Biophys Res Commun. (2016) 469:639–45. doi: 10.1016/j.bbrc.2015.12.052 26703209

[315] Yin H Zhao L Wang Y Li S Huo H Chen H. Duck enteritis virus activates camkkbeta-ampk to trigger autophagy in duck embryo fibroblast cells via increased cytosolic calcium. Virol J. (2018) 15:120. doi: 10.1186/s12985-018-1029-0 30081955 PMC6090797

[316] Saikia R Joseph J. Ampk: A key regulator of energy stress and calcium-induced autophagy. J Mol Med (Berl). (2021) 99:1539–51. doi: 10.1007/s00109-021-02125-8 34398293

[317] Hong JM Moon JH Oh YM Park SY. Calcineurin, calcium-dependent serine-threonine phosphatase activation by prion peptide 106-126 enhances nuclear factor-kappab-linked proinflammatory response through autophagy pathway. ACS Chem Neurosci. (2021) 12:3277–83. doi: 10.1021/acschemneuro.1c00453 34424663

[318] Medina DL Di Paola S Peluso I Armani A De Stefani D Venditti R. Lysosomal calcium signalling regulates autophagy through calcineurin and ​Tfeb. Nat Cell Biol. (2015) 17:288–99. doi: 10.1038/ncb3114 PMC4801004 25720963

[319] Zhu ZD Yu T Liu HJ Jin J He J. Soce induced calcium overload regulates autophagy in acute pancreatitis via calcineurin activation. Cell Death Dis. (2018) 9:50. doi: 10.1038/s41419-017-0073-9 29352220 PMC5833430

[320] Marchi S Patergnani S Pinton P. The endoplasmic reticulum-mitochondria connection: one touch, multiple functions. Biochim Biophys Acta. (2014) 1837:461–9. doi: 10.1016/j.bbabio.2013.10.015 24211533

[321] Rizzuto R De Stefani D Raffaello A Mammucari C. Mitochondria as sensors and regulators of calcium signalling. Nat Rev Mol Cell Biol. (2012) 13:566–78. doi: 10.1038/nrm3412 22850819

[322] Szabadkai G Duchen MR. Mitochondria: the hub of cellular ca2+ Signaling. Physiol (Bethesda). (2008) 23:84–94. doi: 10.1152/physiol.00046.2007 18400691

[323] Friedman JR Nunnari J. Mitochondrial form and function. Nature. (2014) 505:335–43. doi: 10.1038/nature12985 PMC4075653 24429632

[324] Decuypere JP Bultynck G Parys JB. A dual role for ca(2+) in autophagy regulation. Cell Calcium. (2011) 50:242–50. doi: 10.1016/j.ceca.2011.04.001 21571367

[325] Kroemer G Marino G Levine B. Autophagy and the integrated stress response. Mol Cell. (2010) 40:280–93. doi: 10.1016/j.molcel.2010.09.023 PMC3127250 20965422

[326] Gao J Feng W Lv W Liu W Fu C. Hif-1/akt signaling-activated pfkfb2 alleviates cardiac dysfunction and cardiomyocyte apoptosis in response to hypoxia. Int Heart J. (2021) 62:350–8. doi: 10.1536/ihj.20-315 33678793

[327] Colgan SP Curtis VF Lanis JM Glover LE. Metabolic regulation of intestinal epithelial barrier during inflammation. Tissue Barriers. (2015) 3:e970936. doi: 10.4161/21688362.2014.970936 25838978 PMC4372015

[328] Lando D Peet DJ Whelan DA Gorman JJ Whitelaw ML. Asparagine hydroxylation of the hif transactivation domain: A hypoxic switch. Science. (2002) 295:858–61. doi: 10.1126/science.1068592 11823643

[329] Dames SA Martinez-Yamout M De Guzman RN Dyson HJ Wright PE. Structural basis for hif-1α/cbp recognition in the cellular hypoxic response. Proc Natl Acad Sci. (2002) 99:5271–6. doi: 10.1073/pnas.082121399 PMC122759 11959977

[330] Freedman SJ Sun Z-YJ Poy F Kung AL Livingston DM Wagner G. Structural basis for recruitment of cbp/P300 by hypoxia-inducible factor-1α. Proc Natl Acad Sci. (2002) 99:5367–72. doi: 10.1073/pnas.082117899 PMC122775 11959990

[331] Weber A Klocker H Oberacher H Gnaiger E Neuwirt H Sampson N. Succinate accumulation is associated with a shift of mitochondrial respiratory control and hif-1alpha upregulation in pten negative prostate cancer cells. Int J Mol Sci. (2018) 19:2129. doi: 10.3390/ijms19072129 30037119 PMC6073160

[332] Li Y Liu Y Wang C Xia WR Zheng JY Yang J. Succinate induces synovial angiogenesis in rheumatoid arthritis through metabolic remodeling and hif-1alpha/vegf axis. Free Radic Biol Med. (2018) 126:1–14. doi: 10.1016/j.freeradbiomed.2018.07.009 30030103

[333] Lukyanova LD Kirova YI Germanova EL. The role of succinate in regulation of immediate hif-1alpha expression in hypoxia. Bull Exp Biol Med. (2018) 164:298–303. doi: 10.1007/s10517-018-3976-2 29308570

[334] Tannahill GM Curtis AM Adamik J Palsson-McDermott EM McGettrick AF Goel G. Succinate is an inflammatory signal that induces il-1beta through hif-1alpha. Nature. (2013) 496:238–42. doi: 10.1038/nature11986 PMC4031686 23535595

[335] Selak MA Armour SM MacKenzie ED Boulahbel H Watson DG Mansfield KD. Succinate links tca cycle dysfunction to oncogenesis by inhibiting hif-alpha prolyl hydroxylase. Cancer Cell. (2005) 7:77–85. doi: 10.1016/j.ccr.2004.11.022 15652751

[336] Korbecki J Simińska D Gąssowska-Dobrowolska M Listos J Gutowska I Chlubek D. Chronic and cycling hypoxia: drivers of cancer chronic inflammation through hif-1 and nf-κb activation: A review of the molecular mechanisms. Int J Mol Sci. (2021) 22:10701. doi: 10.3390/ijms221910701 34639040 PMC8509318

[337] Minisini M Cricchi E Brancolini C. Acetylation and phosphorylation in the regulation of hypoxia-inducible factor activities: additional options to modulate adaptations to changes in oxygen levels. Life. (2024) 14:20. doi: 10.3390/life14010020 PMC10821055 38276269

[338] Korbecki J Siminska D Gassowska-Dobrowolska M Listos J Gutowska I Chlubek D. Chronic and cycling hypoxia: drivers of cancer chronic inflammation through hif-1 and nf-kappab activation: A review of the molecular mechanisms. Int J Mol Sci. (2021) 22:10701. doi: 10.3390/ijms221910701 34639040 PMC8509318

[339] Bonello S Zähringer C BelAiba RS Djordjevic T Hess J Michiels C. Reactive oxygen species activate the hif-1α Promoter via a functional nfκb site. Arteriosclerosis Thrombosis Vasc Biol. (2007) 27:755–61. doi: 10.1161/01.ATV.0000258979.92828.bc 17272744

[340] André-Lévigne D Modarressi A Pepper MS Pittet-Cuénod B. Reactive oxygen species and nox enzymes are emerging as key players in cutaneous wound repair. Int J Mol Sci. (2017) 18:2149. doi: 10.3390/ijms18102149 29036938 PMC5666831

[341] Diebold I Petry A Sabrane K Djordjevic T Hess J Görlach A. The hif1 target gene nox2 promotes angiogenesis through urotensin-ii. J Cell Sci. (2012) 125:956–64. doi: 10.1242/jcs.094060 22399808

[342] Diebold I Petry A Hess J Gorlach A. The nadph oxidase subunit nox4 is a new target gene of the hypoxia-inducible factor-1. Mol Biol Cell. (2010) 21:2087–96. doi: 10.1091/mbc.e09-12-1003 PMC2883952 20427574

[343] Rahman A Tabassum T Araf Y Al Nahid A Ullah MA Hosen MJ. Silent hypoxia in covid-19: pathomechanism and possible management strategy. Mol Biol Rep. (2021) 48:3863–9. doi: 10.1007/s11033-021-06358-1 PMC8062941 33891272

[344] Belaiba RS Bonello S Zähringer C Schmidt S Hess J Kietzmann T. Hypoxia up-regulates hypoxia-inducible factor-1alpha transcription by involving phosphatidylinositol 3-kinase and nuclear factor kappab in pulmonary artery smooth muscle cells. Mol Biol Cell. (2007) 18:4691–7. doi: 10.1091/mbc.e07-04-0391 PMC2096613 17898080

[345] Frede S Stockmann C Freitag P Fandrey J. Bacterial lipopolysaccharide induces hif-1 activation in human monocytes via P44/42 mapk and nf-kappab. Biochem J. (2006) 396:517–27. doi: 10.1042/bj20051839 PMC1482811 16533170

[346] Malkov MI Lee CT Taylor CT. Regulation of the hypoxia-inducible factor (Hif) by pro-inflammatory cytokines. Cells. (2021) 10:2340. doi: 10.3390/cells10092340 34571989 PMC8466990

[347] Hellwig-B̈rgel T Rutkowski K Metzen E Fandrey J Jelkmann W. Interleukin-1β and tumor necrosis factor- Stimulate DNA binding of hypoxia-inducible factor-1. Blood. (1999) 94:1561–7. doi: 10.1182/blood.V94.5.1561 10477681

[348] Zhou J Schmid T Brüne B. Tumor necrosis factor-α Causes accumulation of a ubiquitinated form of hypoxia inducible factor-1α through a nuclear factor-κb-dependent pathway. Mol Biol Cell. (2003) 14:2216–25. doi: 10.1091/mbc.e02-09-0598 PMC194872 12808024

[349] Kim KW Lee SJ Kim JC. Tnf-α Upregulates hif-1α Expression in pterygium fibroblasts and enhances their susceptibility to vegf independent of hypoxia. Exp Eye Res. (2017) 164:74–81. doi: 10.1016/j.exer.2017.08.008 28803935

[350] Qian D Lin H-Y Wang H-M Zhang X Liu D-L Li Q-L. Normoxic induction of the hypoxic-inducible factor-1α by interleukin-1β Involves the extracellular signal-regulated kinase 1/2 pathway in normal human cytotrophoblast cells1. Biol Reprod. (2004) 70:1822–7. doi: 10.1095/biolreprod.103.025031 14960485

[351] Berra E Pagès G Pouysségur J. Map kinases and hypoxia in the control of vegf expression. Cancer Metastasis Rev. (2000) 19:139–45. doi: 10.1023/A:1026506011458 11191053

[352] Michiels C Minet E Michel G Mottet D Piret J-P Raes M. Hif-1 and ap-1 cooperate to increase gene expression in hypoxia: role of map kinases. IUBMB Life. (2001) 52:49–53. doi: 10.1080/15216540252774766 11795593

[353] Zhang J Zhang Q Lou Y Fu Q Chen Q Wei T. Hypoxia-inducible factor-1α/interleukin-1β Signaling enhances hepatoma epithelial-mesenchymal transition through macrophages in a hypoxic-inflammatory microenvironment. Hepatology. (2018) 67:1872–89. doi: 10.1002/hep.29681 29171040

[354] Nizet V Johnson RS. Interdependence of hypoxic and innate immune responses. Nat Rev Immunol. (2009) 9:609–17. doi: 10.1038/nri2607 PMC4343208 19704417

[355] Hui AS Bauer AL Striet JB Schnell PO Czyzyk-Krzeska MF. Calcium signaling stimulates translation of hif-alpha during hypoxia. FASEB J. (2006) 20:466–75. doi: 10.1096/fj.05-5086com 16507764

[356] Yuan G Nanduri J Khan S Semenza GL Prabhakar NR. Induction of hif-1alpha expression by intermittent hypoxia: involvement of nadph oxidase, ca2+ Signaling, prolyl hydroxylases, and mtor. J Cell Physiol. (2008) 217:674–85. doi: 10.1002/jcp.21537 PMC2696817 18651560

[357] Azimi I. The interplay between hif-1 and calcium signalling in cancer. Int J Biochem Cell Biol. (2018) 97:73–7. doi: 10.1016/j.biocel.2018.02.001 29407528

[358] Azimi I Milevskiy MJG Kaemmerer E Turner D Yapa KTDS Brown MA. Trpc1 is a differential regulator of hypoxia-mediated events and akt signalling in pten-deficient breast cancer cells. J Cell Sci. (2017) 130:2292–305. doi: 10.1242/jcs.196659 28559303

[359] Li Y Guo B Xie Q Ye D Zhang D Zhu Y. Stim1 mediates hypoxia-driven hepatocarcinogenesis via interaction with hif-1. Cell Rep. (2015) 12:388–95. doi: 10.1016/j.celrep.2015.06.033 26166565

[360] Zhu Y Pan Q Meng H Jiang Y Mao A Wang T. Enhancement of vascular endothelial growth factor release in long-term drug-treated breast cancer via transient receptor potential channel 5-ca2+-hypoxia-inducible factor 1α Pathway. Pharmacol Res. (2015) 93:36–42. doi: 10.1016/j.phrs.2014.12.006 25579062

[361] Li S Wang J Wei Y Liu Y Ding X Dong B. Crucial role of trpc6 in maintaining the stability of hif-1α in glioma cells under hypoxia. J Cell Sci. (2015) 128:3317–29. doi: 10.1242/jcs.173161 26187851

[362] Yu S Xu Z Zou C Wu D Wang Y Yao X. Ion channel trpm8 promotes hypoxic growth of prostate cancer cells via an O2-independent and rack1-mediated mechanism of hif-1α Stabilization. J Pathol. (2014) 234:514–25. doi: 10.1002/path.4413 25065497

[363] Chen S-J Hoffman NE Shanmughapriya S Bao L Keefer K Conrad K. A splice variant of the human ion channel trpm2 modulates neuroblastoma tumor growth through hypoxia-inducible factor (Hif)-1/2α*. J Biol Chem. (2014) 289:36284–302. doi: 10.1074/jbc.M114.620922 PMC4276889 25391657

[364] Westra J Brouwer E van Roosmalen IAM Doornbos-van-der-Meer B van Leeuwen MA Posthumus MD. Expression and regulation of hif-1alpha in macrophages under inflammatory conditions; significant reduction of vegf by camkii inhibitor. BMC Musculoskeletal Disord. (2010) 11:61. doi: 10.1186/1471-2474-11-61 PMC2851671 20353560

[365] Semba H Takeda N Isagawa T Sugiura Y Honda K Wake M. Hif-1α-pdk1 axis-induced active glycolysis plays an essential role in macrophage migratory capacity. Nat Commun. (2016) 7:11635. doi: 10.1038/ncomms11635 27189088 PMC4873978

[366] Ullah MS Davies AJ Halestrap AP. The Plasma Membrane Lactate Transporter Mct4, but Not Mct1, Is up-Regulated by Hypoxia through a Hif-1alpha-Dependent Mechanism. J Biol Chem. (2006) 281:9030–7. doi: 10.1074/jbc.M511397200 16452478

[367] Slot IGM Schols AMWJ Vosse BAH Kelders MCJM Gosker HR. Hypoxia differentially regulates muscle oxidative fiber type and metabolism in a hif-1α-dependent manner. Cell Signalling. (2014) 26:1837–45. doi: 10.1016/j.cellsig.2014.04.016 24794533

[368] Lu Y Mao J Han X Zhang W Li Y Liu Y. Downregulated hypoxia-inducible factor 1α Improves myoblast differentiation under hypoxic condition in mouse genioglossus. Mol Cell Biochem. (2021) 476:1351–64. doi: 10.1007/s11010-020-03995-1 33389500

[369] Zhi X Feng W Rong Y Liu R. Anatomy of autophagy: from the beginning to the end. Cell Mol Life Sci. (2018) 75:815–31. doi: 10.1007/s00018-017-2657-z PMC11105611 28939950

[370] Zhang H Bosch-Marce M Shimoda LA Tan YS Baek JH Wesley JB. Mitochondrial autophagy is an hif-1-dependent adaptive metabolic response to hypoxia. J Biol Chem. (2008) 283:10892–903. doi: 10.1074/jbc.M800102200 PMC2447655 18281291

[371] Marsboom G Toth PT Ryan JJ Hong Z Wu X Fang YH. Dynamin-related protein 1-mediated mitochondrial mitotic fission permits hyperproliferation of vascular smooth muscle cells and offers a novel therapeutic target in pulmonary hypertension. Circ Res. (2012) 110:1484–97. doi: 10.1161/circresaha.111.263848 PMC3539779 22511751

[372] Agani FH Pichiule P Chavez JC LaManna JC. The role of mitochondria in the regulation of hypoxia-inducible factor 1 expression during hypoxia. J Biol Chem. (2000) 275:35863–7. doi: 10.1074/jbc.M005643200 10961998

[373] Guzy RD Sharma B Bell E Chandel NS Schumacker PT. Loss of the sdhb, but not the sdha, subunit of complex ii triggers reactive oxygen species-dependent hypoxia-inducible factor activation and tumorigenesis. Mol Cell Biol. (2008) 28:718–31. doi: 10.1128/MCB.01338-07 PMC2223429 17967865

[374] Chandel NS McClintock DS Feliciano CE Wood TM Melendez JA Rodriguez AM. Reactive oxygen species generated at mitochondrial complex iii stabilize hypoxia-inducible factor-1alpha during hypoxia: A mechanism of O2 sensing. J Biol Chem. (2000) 275:25130–8. doi: 10.1074/jbc.M001914200 10833514

[375] Selak MA Armour SM MacKenzie ED Boulahbel H Watson DG Mansfield KD. Succinate links tca cycle dysfunction to oncogenesis by inhibiting hif-&X3b1; prolyl hydroxylase. Cancer Cell. (2005) 7:77–85. doi: 10.1016/j.ccr.2004.11.022 15652751

[376] Lee M Wang C Jin SW Labrecque MP Beischlag TV Brockman MA. Expression of human inducible nitric oxide synthase in response to cytokines is regulated by hypoxia-inducible factor-1. Free Radic Biol Med. (2019) 130:278–87. doi: 10.1016/j.freeradbiomed.2018.10.441 30391674

[377] Wang H Niu F Fan W Shi J Zhang J Li B. Modulating effects of preconditioning exercise in the expression of et-1 and bnp via hif-1α in ischemically injured brain. Metab Brain Dis. (2019) 34:1299–311. doi: 10.1007/s11011-019-00450-z 31222402

[378] Hou E Sun N Zhang F Zhao C Usa K Liang M. Malate and aspartate increase L-arginine and nitric oxide and attenuate hypertension. Cell Rep. (2017) 19:1631–9. doi: 10.1016/j.celrep.2017.04.071 28538181

[379] Edosuyi O Igbe I Oyekan A. Fumarate and its downstream signalling pathways in the cardiorenal system: recent insights and novel expositions in the etiology of hypertension. Eur J Pharmacol. (2023) 961:176186. doi: 10.1016/j.ejphar.2023.176186 37944846 PMC10843741

[380] Ge Y Huang M Yao YM. Autophagy and proinflammatory cytokines: interactions and clinical implications. Cytokine Growth Factor Rev. (2018) 43:38–46. doi: 10.1016/j.cytogfr.2018.07.001 30031632

[381] Al-Zeer MA Al-Younes HM Braun PR Zerrahn J Meyer TF. Ifn-Γ-inducible irga6 mediates host resistance against chlamydia trachomatis via autophagy. PloS One. (2009) 4:e4588. doi: 10.1371/journal.pone.0004588 19242543 PMC2643846

[382] Chang Y-P Tsai C-C Huang W-C Wang C-Y Chen C-L Lin Y-S. Autophagy facilitates ifn-Γ-induced jak2-stat1 activation and cellular inflammation*. J Biol Chem. (2010) 285:28715–22. doi: 10.1074/jbc.M110.133355 PMC2937899 20592027

[383] Wang L Du F Wang H Xie C. Cooperation of cd4+ T cells and cd8+ T cells andrelease of ifn-Γ Are critical for antileukemia responsesof recipient mice treated by microtransplantation. Exp Ther Med. (2018) 15:1532–7. doi: 10.3892/etm.2017.5541 PMC5774513 29399128

[384] Brasseit J Chung CKCK Noti M Zysset D Hoheisel-Dickgreber N Genitsch V. Divergent roles of interferon-Γ and innate lymphoid cells in innate and adaptive immune cell-mediated intestinal inflammation. Front Immunol. (2018) 9:23. doi: 10.3389/fimmu.2018.00023 29416538 PMC5787534

[385] Chen Y-D Fang Y-T Chang C-P Lin C-F Hsu L-J Wu S-R. S100a10 regulates ulk1 localization to er–mitochondria contact sites in ifn-Γ-triggered autophagy. J Mol Biol. (2017) 429:142–57. doi: 10.1016/j.jmb.2016.11.009 27871932

[386] Baregamian N Song J Bailey CE Papaconstantinou J Evers BM Chung DH. Tumor necrosis factor-α and apoptosis signal-regulating kinase 1 control reactive oxygen species release, mitochondrial autophagy and C-jun N-terminal kinase/P38 phosphorylation during necrotizing enterocolitis. Oxid Med Cell Longevity. (2009) 2:893614. doi: 10.4161/oxim.2.5.9541 PMC2835918 20716917

[387] Sivaprasad U Basu A. Inhibition of erk attenuates autophagy and potentiates tumour necrosis factor-α-induced cell death in mcf-7 cells. J Cell Mol Med. (2008) 12:1265–71. doi: 10.1111/j.1582-4934.2008.00282.x PMC3865671 18266953

[388] Jia G Cheng G Gangahar DM Agrawal DK. Insulin-like growth factor-1 and tnf-α Regulate autophagy through C-jun N-terminal kinase and akt pathways in human atherosclerotic vascular smooth cells. Immunol Cell Biol. (2006) 84:448–54. doi: 10.1111/j.1440-1711.2006.01454.x 16942488

[389] Cowan KJ Storey KB. Mitogen-activated protein kinases: new signaling pathways functioning in cellular responses to environmental stress. J Exp Biol. (2003) 206:1107–15. doi: 10.1242/jeb.00220 12604570

[390] Ashwell JD. The many paths to P38 mitogen-activated protein kinase activation in the immune system. Nat Rev Immunol. (2006) 6:532–40. doi: 10.1038/nri1865 16799472

[391] Cuenda A Rousseau S. P38 map-kinases pathway regulation, function and role in human diseases. Biochim Biophys Acta. (2007) 1773:1358–75. doi: 10.1016/j.bbamcr.2007.03.010 17481747

[392] Wuyts WA Vanaudenaerde BM Dupont LJ Demedts MG Verleden GM. Involvement of P38 mapk, jnk, P42/P44 erk and nf-κb in il-1β-induced chemokine release in human airway smooth muscle cells. Respir Med. (2003) 97:811–7. doi: 10.1016/S0954-6111(03)00036-2 12854631

[393] Yi W Wen Y Tan F Liu X Lan H Ye H. Impact of nf-κb pathway on the apoptosis-inflammation-autophagy crosstalk in human degenerative nucleus pulposus cells. Aging (Albany NY). (2019) 11:7294–306. doi: 10.18632/aging.102266 PMC6756901 31518335

[394] Zhu BS Xing CG Lin F Fan XQ Zhao K Qin ZH. Blocking nf-κb nuclear translocation leads to P53-related autophagy activation and cell apoptosis. World J Gastroenterol. (2011) 17:478–87. doi: 10.3748/wjg.v17.i4.478 PMC3027014 21274377

[395] Salminen A Hyttinen JMT Kauppinen A Kaarniranta K. Context-dependent regulation of autophagy by ikk-nf-<B><I>κ</I></B>B signaling: impact on the aging process. Int J Cell Biol. (2012) 2012:849541. doi: 10.1155/2012/849541 22899934 PMC3412117

[396] Trocoli A Djavaheri-Mergny M. The complex interplay between autophagy and nf-κb signaling pathways in cancer cells. Am J Cancer Res. (2011) 1:629–49. PMC3189824 21994903

[397] Pan H Zhang Y Luo Z Li P Liu L Wang C. Autophagy mediates avian influenza H5n1 pseudotyped particle-induced lung inflammation through nf-κb and P38 mapk signaling pathways. Am J Physiology-Lung Cell Mol Physiol. (2013) 306:L183–L95. doi: 10.1152/ajplung.00147.2013 24242010

[398] Ko JH Yoon SO Lee HJ Oh JY. Rapamycin regulates macrophage activation by inhibiting nlrp3 inflammasome-P38 mapk-nfκb pathways in autophagy- and P62-dependent manners. Oncotarget. (2017) 8:40817–31. doi: 10.18632/oncotarget.17256 PMC5522223 28489580

[399] Xiao G. Autophagy and nf-κb: fight for fate. Cytokine Growth Factor Rev. (2007) 18:233–43. doi: 10.1016/j.cytogfr.2007.04.006 PMC2810660 17485237

[400] Xiao J Zhao Z Zhou F Xiong J Yang Z Gong B. Tm9sf1 expression correlates with autoimmune disease activity and regulates antibody production through mtor-dependent autophagy. BMC Med. (2024) 22:502. doi: 10.1186/s12916-024-03729-w 39482663 PMC11526568

[401] Le Tallec E Bellamri N Lelong M Morzadec C Frenger Q Ballerie A. Efferocytosis dysfunction in cxcl4-induced M4 macrophages: phenotypic insights in systemic sclerosis *in vitro* and *in vivo* . Front Immunol. (2024) 15. doi: 10.3389/fimmu.2024.1468821 PMC11512447 39464886

[402] Shen P Deng X Chen Z Chen M Han L Chen X. Demethylzeylasteral ameliorates podocyte damage in murine lupus by inhibiting inflammation and enhancing autophagy. Phytomedicine. (2024) 134:155966. doi: 10.1016/j.phymed.2024.155966 39241387

[403] Ahn SH Lee Y-J Lim DS Cho W Gwon HJ Abd-El-Aty AM. Upadacitinib counteracts hepatic lipid deposition via the repression of jak1/stat3 signaling and ampk/autophagy-mediated suppression of er stress. Biochem Biophys Res Commun. (2024) 735:150829. doi: 10.1016/j.bbrc.2024.150829 39406018

[404] Liu Y Deng S Sun L He H Zhou Q Fan H. Compound sophorae decoction mitigates dss-induced ulcerative colitis by activating autophagy through pi3k-akt pathway: A integrative research combining network pharmacology and *in vivo* animal model validation. J Ethnopharmacology. (2025) 337:118885. doi: 10.1016/j.jep.2024.118885 39369920

[405] Schulze-Osthoff K Ferrari D Riehemann K Wesselborg S. Regulation of nf-κb activation by map kinase cascades. Immunobiology. (1997) 198:35–49. doi: 10.1016/S0171-2985(97)80025-3 9442376

[406] Shang YY Yao M Zhou ZW Jian C Li X Hu RY. Alisertib promotes apoptosis and autophagy in melanoma through P38 mapk-mediated aurora a signaling. Oncotarget. (2017) 8:107076–88. doi: 10.18632/oncotarget.22328 PMC5739797 29291012

[407] Liu J Chang F Li F Fu H Wang J Zhang S. Palmitate promotes autophagy and apoptosis through ros-dependent jnk and P38 mapk. Biochem Biophys Res Commun. (2015) 463:262–7. doi: 10.1016/j.bbrc.2015.05.042 26002468

[408] Wu DJ Adamopoulos IE. Autophagy and autoimmunity. Clin Immunol. (2017) 176:55–62. doi: 10.1016/j.clim.2017.01.007 28095319 PMC5346336

[409] Herrington FD Carmody RJ Goodyear CS. Modulation of nf-κb signaling as a therapeutic target in autoimmunity. J Biomolecular Screening. (2015) 21:223–42. doi: 10.1177/1087057115617456 26597958

[410] Shi CS Kehrl JH. Traf6 and A20 regulate lysine 63-linked ubiquitination of beclin-1 to control tlr4-induced autophagy. Sci Signaling. (2010) 3:ra42. doi: 10.1126/scisignal.2000751 PMC6335036 20501938

[411] Tu SP Quante M Bhagat G Takaishi S Cui G Yang XD. Ifn-Γ Inhibits gastric carcinogenesis by inducing epithelial cell autophagy and T-cell apoptosis. Cancer Res. (2011) 71:4247–59. doi: 10.1158/0008-5472.CAN-10-4009 PMC3139967 21512143

[412] Pun NT Subedi A Kim MJ Park PH. Globular adiponectin causes tolerance to lps-induced tnf-α Expression via autophagy induction in raw 264.7 macrophages: involvement of sirt1/foxo3a axis. PloS One. (2015) 10. doi: 10.1371/journal.pone.0124636 PMC4427353 25961287

[413] Zhang M Kenny SJ Ge L Xu K Schekman R. Translocation of Interleukin-1β into a Vesicle Intermediate in Autophagy- Mediated Secretion. eLife. (2015) 4:e0124636. doi: 10.7554/eLife.11205.001 PMC4728131 26523392

[414] Harris J. Autophagy and il-1 family cytokines. Front Immunol. (2013) 4:83. doi: 10.3389/fimmu.2013.00083 23577011 PMC3617358

[415] Lapaquette P Guzzo J Bretillon L Bringer M-A. Cellular and molecular connections between autophagy and inflammation. Mediat Inflammation. (2015) 2015:398483. doi: 10.1155/2015/398483 PMC4499609 26221063

[416] Scherz-Shouval R Elazar Z. Regulation of autophagy by ros: physiology and pathology. Trends Biochem Sci. (2011) 36:30–8. doi: 10.1016/j.tibs.2010.07.007 20728362

[417] Yun HR Jo YH Kim J Shin Y Kim SS Choi TG. Roles of autophagy in oxidative stress. Int J Mol Sci. (2020) 21:3289. doi: 10.3390/ijms21093289 32384691 PMC7246723

[418] Filomeni G Desideri E Cardaci S Rotilio G Ciriolo MR. Under the ros: thiol network is the principal suspect for autophagy commitment. Autophagy. (2010) 6:999–1005. doi: 10.4161/auto.6.7.12754 20639698

[419] Bensaad K Cheung EC Vousden KH. Modulation of intracellular ros levels by tigar controls autophagy. EMBO J. (2009) 28:3015–26-26. doi: 10.1038/emboj.2009.242 19713938 PMC2736014

[420] Pu W Chu X Guo H Huang G Cui T Huang B. The activated atm/ampk/mtor axis promotes autophagy in response to oxidative stress-mediated DNA damage co-induced by molybdenum and cadmium in duck testes. Environ pollut. (2023) 316:120574. doi: 10.1016/j.envpol.2022.120574 36351481

[421] Li R Luo X Zhu Y Zhao L Li L Peng Q. Atm signals to ampk to promote autophagy and positively regulate DNA damage in response to cadmium-induced ros in mouse spermatocytes. Environ pollut. (2017) 231:1560–8. doi: 10.1016/j.envpol.2017.09.044 28964605

[422] Zhou K Bellenguez C Sutherland C Hardie G Palmer C Donnelly P. The role of atm in response to metformin treatment and activation of ampk. Nat Genet. (2012) 44:361–2. doi: 10.1038/ng.2234 22456734

[423] Glick D Barth S Macleod KF. Autophagy: cellular and molecular mechanisms. J Pathol. (2010) 221:3–12. doi: 10.1002/path.2697 20225336 PMC2990190

[424] Hung CM Garcia-Haro L Sparks CA Guertin DA. Mtor-dependent cell survival mechanisms. Cold Spring Harb Perspect Biol. (2012) 4:a008771. doi: 10.1101/cshperspect.a008771 23124837 PMC3504431

[425] Burgoyne JR. Oxidative stress impairs autophagy through oxidation of atg3 and atg7. Autophagy. (2018) 14:1092–3. doi: 10.1080/15548627.2018.1444311 PMC6103406 29746182

[426] Li L Chen Y Gibson SB. Starvation-induced autophagy is regulated by mitochondrial reactive oxygen species leading to ampk activation. Cell Signal. (2013) 25:50–65. doi: 10.1016/j.cellsig.2012.09.020 23000343

[427] Ney PA. Mitochondrial autophagy: origins, significance, and role of bnip3 and nix. Biochim Biophys Acta (BBA) - Mol Cell Res. (2015) 1853:2775–83. doi: 10.1016/j.bbamcr.2015.02.022 25753537

[428] Saito T Sadoshima J. Molecular mechanisms of mitochondrial autophagy/mitophagy in the heart. Circ Res. (2015) 116:1477–90. doi: 10.1161/CIRCRESAHA.116.303790 PMC4419704 25858070

[429] Wu W Tian W Hu Z Chen G Huang L Li W. Ulk1 translocates to mitochondria and phosphorylates fundc1 to regulate mitophagy. EMBO Rep. (2014) 15:566–75. doi: 10.1002/embr.201438501 PMC4210082 24671035

[430] Lee J Giordano S Zhang J. Autophagy, mitochondria and oxidative stress: cross-talk and redox signalling. Biochem J. (2011) 441:523–40. doi: 10.1042/bj20111451 PMC3258656 22187934

[431] Levonen A-L Hill BG Kansanen E Zhang J Darley-Usmar VM. Redox regulation of antioxidants, autophagy, and the response to stress: implications for electrophile therapeutics. Free Radical Bio Med. (2014) 71:196–207. doi: 10.1016/j.freeradbiomed.2014.03.025 24681256 PMC4042208

[432] Wu J Lei Z Yu J. Hypoxia induces autophagy in human vascular endothelial cells in a hypoxia-inducible factor 1−Dependent manner. Mol Med Rep. (2015) 11:2677–82. doi: 10.3892/mmr.2014.3093 25514934

[433] Liang R Liu N Cao J Liu T Sun P Cai X. Hif-1α/foxo1 axis regulated autophagy is protective for β Cell survival under hypoxia in human islets. Biochim Biophys Acta (BBA) - Mol Basis Dis. (2022) 1868:166356. doi: 10.1016/j.bbadis.2022.166356 35124169

[434] Hubbi ME Hu H Kshitiz Ahmed I Levchenko A Semenza GL. Chaperone-mediated autophagy targets hypoxia-inducible factor-1α (Hif-1α) for lysosomal degradation. J Biol Chem. (2013) 288:10703–14. doi: 10.1074/jbc.M112.414771 PMC3624450 23457305

[435] Bellot G Garcia-Medina R Gounon P Chiche J Roux D Pouysségur J. Hypoxia-induced autophagy is mediated through hypoxia-inducible factor induction of bnip3 and bnip3l via their bh3 domains. Mol Cell Biol. (2009) 29:2570–81. doi: 10.1128/mcb.00166-09 PMC2682037 19273585

[436] Wei J Zhu K Yang Z Zhou Y Xia Z Ren J. Hypoxia-induced autophagy is involved in radioresistance via hif1a-associated beclin-1 in glioblastoma multiforme. Heliyon. (2023) 9:e12820. doi: 10.1016/j.heliyon.2023.e12820 36691538 PMC9860297

[437] Levine B Kroemer G. Biological functions of autophagy genes: A disease perspective. Cell. (2019) 176:11–42. doi: 10.1016/j.cell.2018.09.048 30633901 PMC6347410

[438] Li W He P Huang Y Li YF Lu J Li M. Selective autophagy of intracellular organelles: recent research advances. Theranostics. (2021) 11:222–56. doi: 10.7150/thno.49860 PMC7681076 33391472

[439] Tao S Drexler I. Targeting autophagy in innate immune cells: angel or demon during infection and vaccination? Front Immunol. (2020) 11:460. doi: 10.3389/fimmu.2020.00460 32265919 PMC7096474

[440] Choi Y Bowman JW Jung JU. Autophagy during viral infection — a double-edged sword. Nat Rev Microbiol. (2018) 16:341–54. doi: 10.1038/s41579-018-0003-6 PMC6907743 29556036

[441] Wang K Chen Y Zhang P Lin P Xie N Wu M. Protective features of autophagy in pulmonary infection and inflammatory diseases. Cells. (2019) 8:123. doi: 10.3390/cells8020123 30717487 PMC6406971

[442] Khandia R Dadar M Munjal A Dhama K Karthik K Tiwari R. A comprehensive review of autophagy and its various roles in infectious, non-infectious, and lifestyle diseases: current knowledge and prospects for disease prevention, novel drug design, and therapy. Cells. (2019) 8:674. doi: 10.3390/cells8070674 31277291 PMC6678135

[443] Chen T Tu S Ding L Jin M Chen H Zhou H. The role of autophagy in viral infections. J Biomed Sci. (2023) 30:5. doi: 10.1186/s12929-023-00899-2 36653801 PMC9846652

[444] Morris G Berk M Walder K Maes M. Central pathways causing fatigue in neuro-inflammatory and autoimmune illnesses. BMC Med. (2015) 13:28. doi: 10.1186/s12916-014-0259-2 25856766 PMC4320458

[445] Klimas NG Koneru AO. Chronic fatigue syndrome: inflammation, immune function, and neuroendocrine interactions. Curr Rheumatol Rep. (2007) 9:482–7. doi: 10.1007/s11926-007-0078-y 18177602

[446] Diomedi M Leone G Renna A. The role of chronic infection and inflammation in the pathogenesis of cardiovascular and cerebrovascular disease. Drugs Today (Barc). (2005) 41:745–53. doi: 10.1358/dot.2005.41.11.917342 16395414

[447] Di Rosa F Barnaba V. Persisting viruses and chronic inflammation: understanding their relation to autoimmunity. Immunol Rev. (1998) 164:17–27. doi: 10.1111/j.1600-065x.1998.tb01204.x 9795760

[448] Rasa-Dzelzkaleja S Krumina A Capenko S Nora-Krukle Z Gravelsina S Vilmane A. The persistent viral infections in the development and severity of myalgic encephalomyelitis/chronic fatigue syndrome. J Transl Med. (2023) 21:33. doi: 10.1186/s12967-023-03887-0 36653846 PMC9847171

[449] Gottschalk G Peterson D Knox K Maynard M Whelan RJ Roy A. Elevated atg13 in serum of patients with me/cfs stimulates oxidative stress response in microglial cells via activation of receptor for advanced glycation end products (Rage). Mol Cell Neurosci. (2022) 120:103731. doi: 10.1016/j.mcn.2022.103731 35487443

[450] Khomich OA Kochetkov SN Bartosch B Ivanov AV. Redox biology of respiratory viral infections. Viruses. (2018) 10:392. doi: 10.3390/v10080392 30049972 PMC6115776

[451] Ma Q. Role of nrf2 in oxidative stress and toxicity. Annu Rev Pharmacol Toxicol. (2013) 53:401–26. doi: 10.1146/annurev-pharmtox-011112-140320 PMC4680839 23294312

[452] Gao W Guo L Yang Y Wang Y Xia S Gong H. Dissecting the crosstalk between nrf2 and nf-κb response pathways in drug-induced toxicity. Front Cell Dev Biol. (2022) 9. doi: 10.3389/fcell.2021.809952 PMC8847224 35186957

[453] Soares MP Seldon MP Gregoire IP Vassilevskaia T Berberat PO Yu J. Heme oxygenase-1 modulates the expression of adhesion molecules associated with endothelial cell activation. J Immunol. (2004) 172:3553–63. doi: 10.4049/jimmunol.172.6.3553 15004156

[454] Ganesh Yerra V Negi G Sharma SS Kumar A. Potential therapeutic effects of the simultaneous targeting of the nrf2 and nf-κb pathways in diabetic neuropathy. Redox Biol. (2013) 1:394–7. doi: 10.1016/j.redox.2013.07.005 PMC3757712 24024177

[455] Chen L-G Zhang Y-Q Wu Z-Z Hsieh C-W Chu C-S Wung B-S. Peanut arachidin-1 enhances nrf2-mediated protective mechanisms against tnf-α-induced icam-1 expression and nf-κb activation in endothelial cells. Int J Mol Med. (2018) 41:541–7. 10.3892/ijmm.2017.3238 29115410

[456] Jiang JX. Macrophage nrf2, an anti-inflammatory signal in hepatic ischemia/reperfusion injury. Cell Mol Immunol. (2023) 20:427–8. doi: 10.1038/s41423-022-00964-0 PMC10203325 36600051

[457] Kobayashi EH Suzuki T Funayama R Nagashima T Hayashi M Sekine H. Nrf2 suppresses macrophage inflammatory response by blocking proinflammatory cytokine transcription. Nat Commun. (2016) 7:11624. doi: 10.1038/ncomms11624 27211851 PMC4879264

[458] Liu GH Qu J Shen X. Nf-kappab/P65 antagonizes nrf2-are pathway by depriving cbp from nrf2 and facilitating recruitment of hdac3 to mafk. Biochim Biophys Acta. (2008) 1783:713–27. doi: 10.1016/j.bbamcr.2008.01.002 18241676

[459] Saha S Buttari B Panieri E Profumo E Saso L. An overview of nrf2 signaling pathway and its role in inflammation. Molecules. (2020) 25:5474. doi: 10.3390/molecules25225474 33238435 PMC7700122

[460] Zipper LM Mulcahy RT. Inhibition of erk and P38 map kinases inhibits binding of nrf2 and induction of gcs genes. Biochem Biophys Res Commun. (2000) 278:484–92. doi: 10.1006/bbrc.2000.3830 11097862

[461] Wang H Liu K Chi Z Zhou X Ren G Zhou R. Interplay of mkp-1 and nrf2 drives tumor growth and drug resistance in non-small cell lung cancer. Aging (Albany NY). (2019) 11:11329–46. doi: 10.18632/aging.102531 PMC6932920 31811110

[462] Mylroie H Dumont O Bauer A Thornton C Mackey J Calay D. Pkc -creb-nrf2 signalling induces ho-1 in the vascular endothelium and enhances resistance to inflammation and apoptosis. Cardiovasc Res. (2015) 106:509–19. doi: 10.1093/cvr/cvv131 PMC4431664 25883219

[463] Bloom DA Jaiswal AK. Phosphorylation of Nrf2 at Ser40 by Protein Kinase C in Response to Antioxidants Leads to the Release of Nrf2 from Inrf2, but Is Not Required for Nrf2 Stabilization/Accumulation in the Nucleus and Transcriptional Activation of Antioxidant Response Element-Mediated Nad(P)H:Quinone Oxidoreductase-1 Gene Expression. J Biol Chem. (2003) 278:44675–82. doi: 10.1074/jbc.M307633200 12947090

[464] Granatiero V Konrad C Bredvik K Manfredi G Kawamata H. Nrf2 signaling links er oxidative protein folding and calcium homeostasis in health and disease. Life Sci Alliance. (2019) 2:e201900563. doi: 10.26508/lsa.201900563 31658977 PMC6819749

[465] Cullinan SB Diehl JA. Perk-dependent activation of nrf2 contributes to redox homeostasis and cell survival following endoplasmic reticulum stress. J Biol Chem. (2004) 279:20108–17. doi: 10.1074/jbc.M314219200 14978030

[466] Kidokoro K Kadoya H Cherney DZI Kondo M Wada Y Umeno R. Insights into the regulation of gfr by the keap1-nrf2 pathway. Kidney360. (2023) 4:1454–66. doi: 10.34067/KID.0000000000000171 PMC10615375 37265366

[467] Huang Y Li F. Effects of nrf2 on sarcopplasmic retiulum calcium regulation in C2c12 cells and its mechanism. Acta Med Mediterr. (2019) 35:2917. doi: 10.19193/0393-6384_2019_6_455

[468] Cyran AM Zhitkovich A. Hif1, hsf1, and nrf2: oxidant-responsive trio raising cellular defenses and engaging immune system. Chem Res Toxicol. (2022) 35:1690–700. doi: 10.1021/acs.chemrestox.2c00131 PMC9580020 35948068

[469] Jang J-W Park S Moon E-Y. Spleen tyrosine kinase regulates crosstalk of hypoxia-inducible factor-1α and nuclear factor (Erythroid-derived2)-like 2 for B cell survival. Int Immunopharmacol. (2021) 95:107509. doi: 10.1016/j.intimp.2021.107509 33761438

[470] Wang P Li T Niu C Sun S Liu D. Ros-activated mapk/erk pathway regulates crosstalk between nrf2 and hif-1alpha to promote il-17d expression protecting the intestinal epithelial barrier under hyperoxia. Int Immunopharmacol. (2023) 116:109763. doi: 10.1016/j.intimp.2023.109763 36736221

[471] Lacher SE Skon-Hegg C Ruis BL Krznarich J Slattery M. An antioxidant response element regulates the hif1alpha axis in breast cancer cells. Free Radic Biol Med. (2023) 204:243–51. doi: 10.1016/j.freeradbiomed.2023.05.003 PMC10321210 37179033

[472] Melrose J Smith MM. Natural and semi-synthetic flavonoid anti-sars-cov-2 agents for the treatment of long covid-19 disease and neurodegenerative disorders of cognitive decline. Front Biosci (Elite Ed). (2022) 14:27. doi: 10.31083/j.fbe1404027 36575843

[473] Zinovkin RA Grebenchikov OA. Transcription factor nrf2 as a potential therapeutic target for prevention of cytokine storm in covid-19 patients. Biochem (Mosc). (2020) 85:833–7. doi: 10.1134/S0006297920070111 PMC7356136 33040727

[474] Ujjan ID Khan S Nigar R Ahmed H Ahmad S Khan A. The possible therapeutic role of curcumin and quercetin in the early-stage of covid-19-results from a pragmatic randomized clinical trial. Front Nutr. (2022) 9:1023997. doi: 10.3389/fnut.2022.1023997 36742008 PMC9889936

[475] Imran M Thabet HK Alaqel SI Alzahrani AR Abida A Alshammari MK. The therapeutic and prophylactic potential of quercetin against covid-19: an outlook on the clinical studies, inventive compositions, and patent literature. Antioxidants (Basel). (2022) 11:876. doi: 10.3390/antiox11050876 35624740 PMC9137692

[476] Catalano A Iacopetta D Ceramella J Maio AC Basile G Giuzio F. Are nutraceuticals effective in covid-19 and post-covid prevention and treatment? Foods. (2022) 11:2884. doi: 10.3390/foods11182884 36141012 PMC9498392

[477] Malekmohammad K Rafieian-Kopaei M. Mechanistic aspects of medicinal plants and secondary metabolites against severe acute respiratory syndrome coronavirus 2 (Sars-cov-2). Curr Pharm Des. (2021) 27:3996–4007. doi: 10.2174/1381612827666210705160130 34225607

[478] Rahban M Habibi-Rezaei M Mazaheri M Saso L Moosavi-Movahedi AA. Anti-viral potential and modulation of nrf2 by curcumin: pharmacological implications. Antioxidants (Basel). (2020) 9:1228. doi: 10.3390/antiox9121228 33291560 PMC7761780

[479] Gomaa AA Abdel-Wadood YA Gomaa MA. Glycyrrhizin and boswellic acids, the golden nutraceuticals: multitargeting for treatment of mild-moderate covid-19 and prevention of post-covid cognitive impairment. Inflammopharmacology. (2022) 30:1977–92. doi: 10.1007/s10787-022-01062-3 PMC9493173 36136251

[480] Chrzanowski J Chrzanowska A Grabon W. Glycyrrhizin: an old weapon against a novel coronavirus. Phytother Res. (2021) 35:629–36. doi: 10.1002/ptr.6852 32902005

[481] Bibi S Hasan MM Wang YB Papadakos SP Yu H. Cordycepin as a promising inhibitor of sars-cov-2 rna dependent rna polymerase (Rdrp). Curr Medicinal Chem. (2022) 29:152–62. doi: 10.2174/0929867328666210820114025 34420502

[482] Hetland G Johnson E Bernardshaw SV Grinde B. Can medicinal mushrooms have prophylactic or therapeutic effect against covid-19 and its pneumonic superinfection and complicating inflammation? Scand J Immunol. (2021) 93:e12937. doi: 10.1111/sji.12937 32657436 PMC7404338

[483] Rowaiye AB Ogugua AJ Bur D Wood T Labbo Z Chukwu C. The lingzhi or reishi medicinal mushroom ganoderma lucidum (Agaricomycetes) can combat cytokine storm and other covid-19 related pathologies: A review. Int J Med Mushrooms. (2023) 25:1–15. doi: 10.1615/IntJMedMushrooms.2023048109 37183915

[484] Ho HPT Vo DNK Lin TY Hung JN Chiu YH Tsai MH. Ganoderma microsporum immunomodulatory protein acts as a multifunctional broad-spectrum antiviral against sars-cov-2 by interfering virus binding to the host cells and spike-mediated cell fusion. BioMed Pharmacother. (2022) 155:113766. doi: 10.1016/j.biopha.2022.113766 36271550 PMC9515347

[485] Mueller JK Muller WE. Multi-target drugs for the treatment of cognitive impairment and fatigue in post-covid syndrome: focus on ginkgo biloba and rhodiola rosea. J Neural Transm (Vienna). (2024) 131:203–12. doi: 10.1007/s00702-024-02749-3 PMC10874325 38347175

[486] Akanchise T Angelova A. Ginkgo biloba and long covid: *in vivo* and *in vitro* models for the evaluation of nanotherapeutic efficacy. Pharmaceutics. (2023) 15:1562. doi: 10.3390/pharmaceutics15051562 37242804 PMC10224264

[487] Jamshidi Z Hashemi M Yazdian-Robati R Etemad L Salmasi Z Kesharwani P. Effects of boswellia species on viral infections with particular attention to sars-cov-2. Inflammopharmacology. (2022) 30:1541–53. doi: 10.1007/s10787-022-01037-4 PMC9321285 35882701

[488] Fatima SW Alam S Khare SK. Molecular and structural insights of beta-boswellic acid and glycyrrhizic acid as potent sars-cov-2 envelope protein inhibitors. Phytomed Plus. (2022) 2:100241. doi: 10.1016/j.phyplu.2022.100241 35403092 PMC8840829

[489] Roy A Menon T. Evaluation of bioactive compounds from boswellia serrata against sars-cov-2. Vegetos. (2022) 35:404–14. doi: 10.1007/s42535-021-00318-7 PMC8595075 34803247

[490] Liu L Wang Y Wang W Ying W Sun B Wang X. Increased expression of the tlr7/9 signaling pathways in chronic active ebv infection. Front Pediatr. (2022) 10:1091571. doi: 10.3389/fped.2022.1091571 36619523 PMC9811674

[491] Akazawa R Otsuka S Kato I Imadome KI Takita J. Transient remission of chronic active ebv infection after chemotherapy alone. Pediatr Int. (2022) 64:e14836. doi: 10.1111/ped.14836 34897890

[492] Wei A Ma H Zhang L Li Z Guan Y Zhang Q. Clinical analysis of chronic active ebv infection with coronary artery dilatation and a matched case-control study. Orphanet J Rare Dis. (2021) 16:50. doi: 10.1186/s13023-021-01689-5 33509232 PMC7845094

[493] Aihara Y Moriya K Shimozato N Nagamatsu S Kobayashi S Uejima M. Chronic active ebv infection in refractory enteritis with longitudinal ulcers with a cobblestone appearance: an autopsied case report. BMC Gastroenterol. (2021) 21:6. doi: 10.1186/s12876-020-01589-1 33407170 PMC7789587

[494] Yonese I Sakashita C Imadome KI Kobayashi T Yamamoto M Sawada A. Nationwide survey of systemic chronic active ebv infection in Japan in accordance with the new who classification. Blood Adv. (2020) 4:2918–26. doi: 10.1182/bloodadvances.2020001451 PMC7362364 32598475

[495] Sakaki S Imadome KI Kawano F Nakadate H Ishiguro A. Shift in epstein-barr virus (Ebv)-infected cells in chronic active ebv disease. Pediatr Int. (2019) 61:825–6. doi: 10.1111/ped.13935 31436003

[496] Schmaltz HN Fried LP Xue QL Walston J Leng SX Semba RD. Chronic cytomegalovirus infection and inflammation are associated with prevalent frailty in community-dwelling older women. J Am Geriatr Soc. (2005) 53:747–54. doi: 10.1111/j.1532-5415.2005.53250.x 15877548

[497] Keita AK Vidal N Toure A Diallo MSK Magassouba Nf Baize S. A 40-month follow-up of ebola virus disease survivors in Guinea (Postebogui) reveals long-term detection of ebola viral ribonucleic acid in semen and breast milk. Open Forum Infect Dis. (2019) 6:ofz482. doi: 10.1093/ofid/ofz482 32128327 PMC7047953

[498] Paz-Bailey G Rosenberg ES Doyle K Munoz-Jordan J Santiago GA Klein L. Persistence of zika virus in body fluids - final report. N Engl J Med. (2018) 379:1234–43. doi: 10.1056/NEJMoa1613108 PMC5831142 28195756

[499] Chia JK Chia AY. Chronic fatigue syndrome is associated with chronic enterovirus infection of the stomach. J Clin Pathol. (2008) 61:43–8. doi: 10.1136/jcp.2007.050054 17872383

[500] Riddell MA Moss WJ Hauer D Monze M Griffin DE. Slow clearance of measles virus rna after acute infection. J Clin Virol. (2007) 39:312–7. doi: 10.1016/j.jcv.2007.05.006 17625962

[501] Yao Q Doyle Máire E Liu Q-R Appleton A O’Connell Jennifer F N-p W. Long-term dysfunction of taste papillae in sars-cov-2. NEJM Evidence. (2023) 2:EVIDoa2300046. doi: 10.1056/EVIDoa2300046 PMC10745124 38145006

[502] André FR Hiranmayi R Junbum K Alain CB Olivier E Robert ES. Persistent alveolar type 2 dysfunction and lung structural derangement in post-acute covid-19. medRxiv. (2022):2022.11.28.22282811. doi: 10.1101/2022.11.28.22282811

[503] de Melo GD Lazarini F Levallois S Hautefort C Michel V Larrous F. Covid-19–related anosmia is associated with viral persistence and inflammation in human olfactory epithelium and brain infection in hamsters. Sci Trans Med. (2021) 13:eabf8396. doi: 10.1126/scitranslmed.abf8396 PMC8158965 33941622

[504] Chun Chau Lawrence C Denise G Xinru L Tracy Zhijun T Jeffrey Chun Tatt L Justina Nadia L. Residual sars-cov-2 viral antigens detected in gi and hepatic tissues from five recovered patients with covid-19. Gut. (2022) 71:226. doi: 10.1136/gutjnl-2021-324280 34083386

[505] Hany M Zidan A Gaballa M Ibrahim M Agayby ASS Abouelnasr AA. Lingering sars-cov-2 in gastric and gallbladder tissues of patients with previous covid-19 infection undergoing bariatric surgery. Obes Surg. (2023) 33:139–48. doi: 10.1007/s11695-022-06338-9 PMC9628579 36316598

[506] Miura CS Lima TM Martins RB Jorge DMM Tamashiro E Anselmo-Lima WT. Asymptomatic Sars-Cov-2 Infection in Children's Tonsils. Braz J Otorhinolaryngol (2022) 88:9.32456874

[507] Lan L Xu D Ye G Xia C Wang S Li Y. Positive rt-pcr test results in patients recovered from covid-19. JAMA. (2020) 323:1502–3. doi: 10.1001/jama.2020.2783 PMC7047852 32105304

[508] Zuo T Liu Q Zhang F Lui GC-Y Tso EY Yeoh YK. Depicting sars-cov-2 faecal viral activity in association with gut microbiota composition in patients with covid-19. Gut. (2021) 70:276–84. doi: 10.1136/gutjnl-2020-322294 PMC7385744 32690600

[509] Tejerina F Catalan P Rodriguez-Grande C Adan J Rodriguez-Gonzalez C Munoz P. Post-covid-19 syndrome. Sars-cov-2 rna detection in plasma, stool, and urine in patients with persistent symptoms after covid-19. BMC Infect Dis. (2022) 22:211. doi: 10.1186/s12879-022-07153-4 35240997 PMC8892394

[510] Proal AD VanElzakker MB Aleman S Bach K Boribong BP Buggert M. Sars-cov-2 reservoir in post-acute sequelae of covid-19 (Pasc). Nat Immunol. (2023) 24:1616–27. doi: 10.1038/s41590-023-01601-2 37667052

[511] van Steijn JH van Tol KM van Essen LH Gans RO. Disseminated intravascular coagulation as an unusual presentation of an epstein-barr virus infection. Neth J Med. (2000) 57:169–71. doi: 10.1016/s0300-2977(00)00047-4 11006494

[512] Muller NF Schampera M Jahn G Malek NP Berg CP Hamprecht K. Case report: severe cytomegalovirus primary infection in an immunocompetent adult with disseminated intravascular coagulation treated with valganciclovir. BMC Infect Dis. (2016) 16:19. doi: 10.1186/s12879-016-1343-3 26787617 PMC4719720

[513] Niewold TB Bundrick JB. Disseminated intravascular coagulation due to cytomegalovirus infection in an immunocompetent adult treated with plasma exchange. Am J Hematol. (2006) 81:454–7. doi: 10.1002/ajh.20602 16680750

[514] Hiemstra S Fehling-Kaschek M Kuijper IA Bischoff LJM Wijaya LS Rosenblatt M. Dynamic modeling of nrf2 pathway activation in liver cells after toxicant exposure. Sci Rep. (2022) 12:7336. doi: 10.1038/s41598-022-10857-x 35513409 PMC9072554

[515] Lupu A Stoleriu G Nedelcu AH Perju SN Gavrilovici C Baciu G. Overview of oxidative stress in systemic lupus erythematosus. Antioxidants-Basel. (2025) 14:303. doi: 10.3390/antiox14030303 40227251 PMC11939823

[516] Kaushal GP Chandrashekar K Juncos LA. Molecular interactions between reactive oxygen species and autophagy in kidney disease. Int J Mol Sci. (2019) 20:3791. doi: 10.3390/ijms20153791 31382550 PMC6696055

[517] Czaja AJ. Nature and implications of oxidative and nitrosative stresses in autoimmune hepatitis. Digestive Dis Sci. (2016) 61:2784–803. doi: 10.1007/s10620-016-4247-6 27411555

[518] Pennisi M Crupi R Di Paola R Ontario ML Bella R Calabrese EJ. Inflammasomes, hormesis, and antioxidants in neuroinflammation: role of nrlp3 in alzheimer disease. J Neurosci Res. (2017) 95:1360–72. doi: 10.1002/jnr.23986 27862176

[519] Misrani A Tabassum S Yang L. Mitochondrial dysfunction and oxidative stress in alzheimer’s disease. Front Aging Neurosci. (2021) 13:617588. doi: 10.3389/fnagi.2021.617588 33679375 PMC7930231

[520] Dinkova-Kostova AT Kostov RV Kazantsev AG. The role of nrf2 signaling in counteracting neurodegenerative diseases. FEBS J. (2018) 285:3576–90. doi: 10.1111/febs.14379 PMC6221096 29323772

[521] Donia T Khamis A. Management of oxidative stress and inflammation in cardiovascular diseases: mechanisms and challenges. Environ Sci pollut Res. (2021) 28:34121–53. doi: 10.1007/s11356-021-14109-9 33963999

[522] da Costa RM Rodrigues D Pereira CA Silva JF Alves JV Lobato NS. Nrf2 as a potential mediator of cardiovascular risk in metabolic diseases. Front Pharmacol. (2019) 10:382. doi: 10.3389/fphar.2019.00382 31031630 PMC6473049

[523] Zhou Y Murugan DD Khan H Huang Y Cheang WS. Roles and therapeutic implications of endoplasmic reticulum stress and oxidative stress in cardiovascular diseases. Antioxidants-Basel. (2021) 10:1167. doi: 10.3390/antiox10081167 34439415 PMC8388996

[524] Varga ZV Giricz Z Liaudet L Haskó G Ferdinandy P Pacher P. Interplay of oxidative, nitrosative/nitrative stress, inflammation, cell death and autophagy in diabetic cardiomyopathy. Biochim Biophys Acta (BBA) - Mol Basis Dis. (2015) 1852:232–42. doi: 10.1016/j.bbadis.2014.06.030 PMC4277896 24997452

[525] David JA Rifkin WJ Rabbani PS Ceradini DJ. The nrf2/keap1/are pathway and oxidative stress as a therapeutic target in type ii diabetes mellitus. J Diabetes Res. (2017) 2017:4826724. doi: 10.1155/2017/4826724 28913364 PMC5585663

[526] Rodrigo R Libuy M Feliú F Hasson D. Oxidative stress-related biomarkers in essential hypertension and ischemia-reperfusion myocardial damage. Dis Markers. (2013) 35:974358. doi: 10.1155/2013/974358 PMC3856219 24347798

[527] Niemann B Rohrbach S Miller Mark R Newby David E Fuster V Kovacic Jason C. Oxidative stress and cardiovascular risk: obesity, diabetes, smoking, and pollution. JACC. (2017) 70:230–51. doi: 10.1016/j.jacc.2017.05.043 PMC5568826 28683970

[528] Masenga SK Kabwe LS Chakulya M Kirabo A. Mechanisms of oxidative stress in metabolic syndrome. Int J Mol Sci. (2023) 24:7898. doi: 10.3390/ijms24097898 37175603 PMC10178199

[529] Ramos-Tovar E Muriel P. Molecular mechanisms that link oxidative stress, inflammation, and fibrosis in the liver. Antioxidants-Basel. (2020) 9:1279. doi: 10.3390/antiox9121279 33333846 PMC7765317

[530] Conde de la Rosa L Goicoechea L Torres S Garcia-Ruiz C Fernandez-Checa JC. Role of oxidative stress in liver disorders. Livers. (2022) 2:283–314. doi: 10.3390/livers2040023

[531] Allameh A Niayesh-Mehr R Aliarab A Sebastiani G Pantopoulos K. Oxidative stress in liver pathophysiology and disease. Antioxidants-Basel. (2023) 12:1653. doi: 10.3390/antiox12091653 37759956 PMC10525124

[532] Białas AJ Sitarek P Miłkowska-Dymanowska J Piotrowski WJ Górski P. The role of mitochondria and oxidative/antioxidative imbalance in pathobiology of chronic obstructive pulmonary disease. Oxid Med Cell Longevity. (2016) 2016:7808576. doi: 10.1155/2016/7808576 PMC5220474 28105251

[533] Cipollina C Bruno A Fasola S Cristaldi M Patella B Inguanta R. Cellular and molecular signatures of oxidative stress in bronchial epithelial cell models injured by cigarette smoke extract. Int J Mol Sci. (2022) 23:1770. doi: 10.3390/ijms23031770 35163691 PMC8836577

[534] Di Stefano A Maniscalco M Balbi B Ricciardolo FLM. Oxidative and nitrosative stress in the pathogenesis of obstructive lung diseases of increasing severity. Curr Medicinal Chem. (2020) 27:7149–58. doi: 10.2174/0929867327666200604165451 32496983

[535] Juszczyk G Mikulska J Kasperek K Pietrzak D Mrozek W Herbet M. Chronic stress and oxidative stress as common factors of the pathogenesis of depression and alzheimer’s disease: the role of antioxidants in prevention and treatment. Antioxidants-Basel. (2021) 10:1439. doi: 10.3390/antiox10091439 34573069 PMC8470444

[536] Gan H Ma Q Hao W Yang N Chen Z-S Deng L. Targeting autophagy to counteract neuroinflammation: A novel antidepressant strategy. Pharmacol Res. (2024) 202:107112. doi: 10.1016/j.phrs.2024.107112 38403256

[537] Sani G Margoni S Brugnami A Ferrara OM Bernardi E Simonetti A. The nrf2 pathway in depressive disorders: A systematic review of animal and human studies. Antioxidants-Basel. (2023) 12:817. doi: 10.3390/antiox12040817 37107192 PMC10135298

[538] Bryll A Skrzypek J Krzyściak W Szelągowska M Śmierciak N Kozicz T. Oxidative-antioxidant imbalance and impaired glucose metabolism in schizophrenia. Biomolecules. (2020) 10:384. doi: 10.3390/biom10030384 32121669 PMC7175146

[539] De Simone G Mazza B Vellucci L Barone A Ciccarelli M de Bartolomeis A. Schizophrenia synaptic pathology and antipsychotic treatment in the framework of oxidative and mitochondrial dysfunction: translational highlights for the clinics and treatment. Antioxidants-Basel. (2023) 12:975. doi: 10.3390/antiox12040975 37107350 PMC10135787

[540] Morris G Puri BK Walder K Berk M Stubbs B Maes M. The endoplasmic reticulum stress response in neuroprogressive diseases: emerging pathophysiological role and translational implications. Mol Neurobiol. (2018) 55:8765–87. doi: 10.1007/s12035-018-1028-6 PMC6208857 29594942

[541] You J Li Y Chong W. The role and therapeutic potential of sirts in sepsis. Front Immunol. (2024) 15:1394925. doi: 10.3389/fimmu.2024.1394925 38690282 PMC11058839

